# Supplementary material for: Transcriptome-Wide Identification of Neuropeptides and Neuropeptide Receptors in the Twenty-Eight-Spotted Ladybird Henosepilachna vigintioctopunctata
Source: Insects. 2025 Jun 13;16(6):624. doi: 10.3390/insects16060624 (PMC12194362; doi:10.3390/insects16060624)
Supplement: Supplementary file 1 [file insects-16-00624-s001.zip › insects-3627983-supplementary.pdf]

Supplementary Materials

Article

Transcriptome-Wide Identification of Neuropeptides and Neuropeptide Receptors in the Twenty-Eight-Spotted Ladybird *Henosepilachna vigintioctopunctata*

Quanxing Lei [1], Ziming Wang [1], Shuangyan Yao [1], Aili Lin [2], Yunhui Zhang [1], Chengxian Sun [1], Xiaoguang Liu [1], Mengfang Du [1], Xiaoming Liu [1,*] and Shiheng An [1]

[1] Henan International Laboratory for Green Pest Control, College of Plant Protection, Henan Agricultural University, Zhengzhou 450046, China; leiquanxing526@163.com (Q.L.); zimingwang2024@163.com (Z.W.); yaoshuangyan@outlook.com (S.Y.); 13403721591@163.com (Y.Z.); wsyenan@163.com (C.S.); xgliu2000@aliyun.com (X.L.); dumengfang@163.com (M.D.); anshiheng@aliyun.com (S.A.)

[2] Henan International Joint Laboratory of Taxonomy and Systematic Evolution of Insecta, Henan Institute of Science and Technology, Xinxiang 453003, China; linaili2023@126.com

* Correspondence: liuxiaoming0318@126.com

Table S1. Summary of RNA-Seq data in *Henosepilachna vigintioctopunctata* central nervous system transcriptome.
Table S2. Summary of assembled unigenes in *H. vigintioctopunctata* central nervous system transcriptome.
Table S3. Sequences for putative transcriptions of annotated neuropeptide precursors genes deduced from *H. vigintioctopunctata* central nervous system transcriptome.
Table S4. Overview of the presence of neuropeptide precursors in *H. vigintioctopunctata* and other insects.
Table S5. G protein-coupled receptors for neuropeptide identified in *H. vigintioctopunctata.*
Table S6. Primers in this study.
Table S7. GenBank accession numbers used for the multiple sequence alignment.
Figure S1. Functional annotation of unigenes from transcriptome of *H. vigintioctopunctata* CNS.
Figure S2. GO annotations analysis of unigenes from transcriptome of *H. vigintioctopunctata* CNS.
Figure S3 The standard curves, melting curves, amplification plots of the primers for Quantitative Real-Time PCR (qRT-PCR) of 58 neuropeptide genes.
Figure S4. Sequence alignment of Adipokinetic hormone (AKH) precursors.
Figure S5. Sequence alignment of Agatoxin-like (ALP) precursors.
Figure S6. Sequence alignment of Allatostatin B (AstB) precursors.
Figure S7. Sequence alignment of Allatostatin CC (AstCC) precursors.
Figure S8. Sequence alignment of Allatostatin CCC (AstCCC) precursors.
Figure S9. Sequence alignment of Allatotropin (AT) precursors.
Figure S10. Sequence alignment of Antidiuretic factor b-1 (ADF-b1) precursors.
Figure S11. Sequence alignment of Antidiuretic factor b-4 (ADF-b4) precursors.
Figure S12. Sequence alignment of Antidiuretic factor b-5 (ADF-b5) precursors.
Figure S13. Sequence alignment of Arginine-vasopressin-like (AVPL) precursors.
Figure S14. Sequence alignment of Baratin (NVP-like) precursors.
Figure S15. Sequence alignment of bursicon alpha (bur α) precursors.
Figure S16. Sequence alignment of bursicon beta (bur β) precursors.
Figure S17. Sequence alignment of Capability (CAPA) precursors.
Figure S18. Sequence alignment of Crustacean cardioactive peptide (CCAP) precursors.
Figure S19. Sequence alignment of CCHamide 1 (CCH1) precursors.
Figure S20. Sequence alignment of CCHamide 2 (CCH2) precursors.
Figure S21. Sequence alignment of CNMamide (CNMa) precursors.
Figure S22. Sequence alignment of Calcitonin-like diuretic hormone 31 (CL-DH31) precursors.
Figure S23. Sequence alignment of Corticotropin-releasing factor-like-diuretic hormone 37 (CRF-DH 37) precursors.
Figure S24. Sequence alignment of Corticotropin-releasing factor-like-diuretic hormone 44 (CRF-DH 44) precursors.
Figure S25. Sequence alignment of Eclosion hormone (EH) precursors.
Figure S26. Sequence alignment of Ecdysis triggering hormone (ETH) precursors.
Figure S27. Sequence alignment of FMRFamide (FMRF) precursors.

Figure S28. Sequence alignment of Glycoprotein hormone alpha 2 (GPA2) precursors.
Figure S29. Sequence alignment of Glycoprotein hormone beta 5 (GPB5) precursors.
Figure S30. Sequence alignment of Hansolin precursors.
Figure S31. Sequence alignment of IDLSRF-like peptide (IDLSRF) precursors.
Figure S32. Sequence alignment of Insulin-like peptide (ILP) precursors.
Figure S33. Sequence alignment of ion transport peptide (ITP) precursors.
Figure S34. Sequence alignment of ITG-like (ITG) precursors.
Figure S35. Sequence alignment of Myosuppressin (MS) precursors.
Figure S36. Sequence alignment of Natalisin (NTL) precursors.
Figure S37. Sequence alignment of Neuroparsin A (NPA) precursors.
Figure S38. Sequence alignment of Neuropeptide F 1a (NPF1a) precursors.
Figure S39. Sequence alignment of Neuropeptide F 1b (NPF1b) precursors.
Figure S40. Sequence alignment of Neuropeptide-like precursor 1 (NPLP1) precursors.
Figure S41. Sequence alignment of Orcokinin A (OK A) precursors.
Figure S42. Sequence alignment of Orcokinin B (OK B) precursors.
Figure S43. Sequence alignment of Pigment-dispersing factor (PDF) precursors.
Figure S44. Sequence alignment of Prothoracicotropic hormone (PTTH) precursors.
Figure S45. Sequence alignment of Proctolin (Pro) precursors.
Figure S46. Sequence alignment of Pyrokinin (PK)/phermone biosynthesis activating neuropeptide like (PBAN-like) precursors.
Figure S47. Sequence alignment of RFLamide (RF) precursors.
Figure S48. Sequence alignment of Ryamide (RY) precursors.
Figure S49. Sequence alignment of short neuropeptide F (sNPF) precursors.
Figure S50. Sequence alignment of SIFamide (SIF) precursors.
Figure S51. Sequence alignment of Sulfakinin (SK) precursors.
Figure S52. Sequence alignment of Tachykinin (TK) precursors.
Figure S53. Sequence alignment of Trissin (TR) precursors.

Table S1. Summary of RNA-Seq data in *Henosepilachna vigintioctopunctata* central nervous system transcriptome.

Statistics project	Number
Total raw reads (bp)	11,784,220,898
Total clean reads (bp)	11,550,286,624
Error rate (%)	0.0117
Clean reads Q20 (%)	98.95
Clean reads Q30 (%)	96.62
GC content (%)	35.30

Table S2. Summary of assembled unigenes in *H. vigintioctopunctata* central nervous system transcriptome.

Statistics project	Number	Percent of Unigene
200 - 500 bp	9,860	43%
501 - 1000 bp	4,220	18%
1001 - 1500 bp	2,295	10%
1501 - 2000 bp	1,804	8%
> 2000 bp	4,861	22%
Total number	23,040	-
Total Length (bp)	29,607,877	-
Largest length (bp)	16,649	-
Smallest length (bp)	201	-
Average length (bp)	1285	-
N50 length (bp)	2476	-
E90N50 length (bp)	3060	-
Fragment mapped percent (%)	82.906	-
GC content (%)	34.14	-
TransRate score	0.45771	-
BUSCO score	C:96.1% [S:94.7%; D:1.4%]	-

Table S3. Sequences for putative transcriptions of annotated neuropeptide precursors genes deduced from *H. vigintioctopunctata* central nervous system transcriptome.

Underlined letters are the predicted signal peptides made by hidden markov models (http://www.cbs.dtu.dk/services/SignalP/, (Almagro Armenteros et al., 2019)). Red fonts are putative active peptides based on the homology. Gray N-terminal sequences are the putative translation initiation site in the largest open reading frame, but lacking significant signal peptide sequence predicted. Green highlights are for canonical dibasic cleavage and amidation sites.

>Adipokinetic hormone 2 (AKH2)
MHRSVLLVFAAICFWSMCTAQLNFTPNWGKRGSSIPENLDNCRSSMMDTIMVIYKVIENEAQKLIQCEKMAN
>Agatoxin-like peptide (ALP)
MKTSWLVLIALVMIVELTTQIGAESYDEDENYLPEDEYSENAIDRLLQPAQKRSSLIYLFRRACIRRGGNCDHRPNDCCYNSSCRCNLWGSNCRCQRMGLFQKWGK
>Allatostatin B (AstB)/Prothoracicostatic peptide (PTSP)/Myoinhibitory peptide (MIP)
MRHAFSPVAQKLIGAVILAFYLQINFCDAQPEDTPVYPKSEDDNLQVGSDLVKRNWDKNPKIWGKRAWSNLHGGWGVKRSVPDQFDQDSTIADKRSWENLRSGWGKRRISPEDLTPQELSLLENESQYPDLEGFYEFNDDEKRSWNQLHSGWGKRNKWSEFRGAWGKRGPDPAWNNLKGIWGKRSYPDRMFV
>Allatostatin CC (AstCC) X1
MLQLGLAAQIFMVVVLLNQEVFGYVIDKKSAAPERNAEDYADYQLGVKYDEYPMIVPKKRTAMLVDRLMVALKEAIDEEEAQMANSIIDNPSRTKPYALSPEDVRTMDLQRRGHGSLSTESKVRPYWRCYFNAVTCF
>Allatostatin CC (AstCC) X2
MKTMLQLGLAAQIFMVVVLLNREVFGYVIDKKSAAPERNAEDYADYQLGVKYDEYPMIVPKKRTAMLVDRLMVALKEAIDEEEAQMANSIIDNPSRTKPYALSPEDVRTMDLQRRGHGSLSTESKVRPYWRCYFNAVTCF
>Allatostatin CCC (AstCCC)
MSSELVKMQRFLVLCLLIAVTAAKPSHFTDNQVSPDAEKGDFMDPGLLRALERYDALRRLASDYENANILNQNPLEPLFRNTEMKRQGRSYRPCYFNPISCFKK
>Allatotropin (AT)
MELHHVIVLLVIILLWANLASVQGRKRDDMPRAFRRSADASERRTRKFDEMVLATARGYGKRANERFKDNSLLEWIGLERMRGLGYPTRVMSNSEMISE
>Antidiuretic factor b-1 (ADF-b1)
MNALSLVVLLVAVAAASASILAAPGALIGPSGAIIAGRGAIAAGPLGLGLANPLGLGLASPLGLGLGGHLGLAAPLGVRVTATNLRVAPAGAPVGVLVPAGNGLEGQWVPEISEKLHDDGSYKPEIYGA
>Antidiuretic factor b-4 (ADF-b4)
MNAFTILFAFGAFAAANAGLLPLVASRAVVSPYVAGAVVVPRGHGFEGQYIPDHLEHLYDDGSYRPQLRAQPLSPSPYGLVPSGSGLEGAYVHDLTEKLFDDGSYKPELRAQPLSPSPYGLVPSGSGLEGQYVHDFTEKLYDDGSYKPELSVGVKHLIV
>Antidiuretic factor b-5 (ADF-b5)

MNCLGAITFLAVVAVAAASGIHGIHGGAVIAGPSGSIAANGLGGAAVVGPTGPNLGHAG
VGIIAPGVIAARGLGLGLAGHGLGLGHGLGLGLGGLGLAAHLDVGHGGLVGSGIEGQWV
PDINEHLYDDGSYKPHVYGH

>Arginine-vasopressin-like (AVPL)

MLKNLMILSAVFLVYALLVDSCLITNCPRGGKRNGKNGRIEVKQCVSCGPGRTGQCFGP
GICCGPFGCLVGTPDTVRCQREGYFHESEPCIAGSSSCRRNTARCAAEGICCNQESCHMDS
SCTLDEKSRTQENIFPLDIYNFLSYQTEDVKK

>Baratin (NVP-like)

MEHQRSIRLLICIFCIYGALAIPSSMVEEIKARELKPNKVKRVSLPPQSPEENLKNGNHYYF
NPSAIKRGAAFRNPAIEDDVTNDSDWSPEEQNVYEPTDNDFQSSLYTDENSQDGKTLNEY
EKGFQYGTNKEKLDEALENAVLKSEIYGGSPSSINRYRYLENDKKKRRKRDSRKSNYGN
RATSDDLTPEEVLALLALYENERQPYQKPVEDLNDLEQDSDTWLDVPVRPHPELPTPSEL
GPAYLMDQNIPVFERSRWVENKDKRFMVSKRRNDPTRELRYLTGPSKHDYYTLSQLLSN
QREANVPVFHRYIL

>Bursicon alpha (Burα)

MFNRIFRITSVSWNILSRTLLCIVLGSMCLDPRYTKYEVSSASTTDECQVTPVIHVLQYPGC
VPKPIPSFACIGRCASYIQVSGSKIWQMERSCMCCQESGEREASVSLFCPKAKPGERKFIKV
TTKAPLECMCRPCTGVEESAIIPQEIAGYADEGPLSNHFLKSQPQ

>Bursicon beta (Burβ)

MAIVGRRLGQNILVLCIVISLGFCVSEIADETCETLPSEIHLVKEEYDELGRLQRTCNGEIA
VNKCEGSCKSQVQPSVITPTGFLKECYCCRESFLRERIITLNHCYDPDGMRLTNEGNNAM
DVKLREPAECKCYKCGDFSR

>Capability (CAPA)/Periviscerokinin (PVK)/Cardioacceleratory peptide 2b (CAP2b)

MNILFFLCVFMLPLASVFSNDASIPNRESKLGGDPKFHFFRIGRGKESANPVGSMSIFPRVG
RDPSRVYTANDFESENKRSSDSNTALWFGPRLGVHKRDGHETPYTYILLNDGNEYRPEYS
ILPGKNFQVDDEYQSMVFEPIN

>Crustacean cardioactive peptide (CCAP)

MLPTIVFSALIASMFVNEAQLFLLQKKGGMYAIEERVEKLTDPKKRPFCNAFTGCGRKRS
NIPALTNNGEELDDSISTLLELNAEPAVENLSRQIMSEAKLWEAIQEANVELNRRRQETKG
NVNGATQAKSPAPCVLPSCYI

>CCHamide 1 (CCH1)

MKKNCVAMDDFSHTAVFRLTVLLTFFLFAECAAGSCLSYGHSCWGAHGKRSGYIGNPK
YLDSSRLLMRLMQQNANHRSEMDKEVMRDYENVPSRAEVQQLINDVEMESNPMKTLSD
YEADSDVLFDDNVPVFEGITENHLKPSNTRRISQRILKKRSTKMN

>CCHamide 2(CCH2)

MGFWSTAALSLAFLVLLVVLNFGGAQGKRGCANFGHSCYGGMGKRALDIASSNDEFVA
DMPYAQDAGLVFTGPRSGGYNSYPRERMSPQQYEQLSRILKQWIQFHKSPQSYQRDENM
V

>CNMamide (CNMa)

MIILLGIILVSRVNCKSFLANLSENSDYRSLNPEMMENINEEFDTYLDLGGANGDSDEYDIL
SEQIFSNKLNTGRQFGLNKDRELIQTYILVKTMRQRNKRYVNYLTLCHFKICTIGRRNARH
ENVLKRERKAPFKFRSKKN

>Calcitonin-like diuretic hormone 31 (CL-DH31)

MNSQKKITAALLIVMVLSSLILLSTPTQIAKKNLDLGVGRGFSGCQAAKQRLGLALANLV
VGPGKRSKK

>Corticotropin releasing factor like-DH 37 X1 (CRF-DH37)

MRLPIYLICAAFVLAIEAEENAVDYYGSFLEPMKISSEREPLNNYMLPKVSAKYRPEWVR
LPEPSYYMPEEEVNADADRLGLFRKNFVKKQYESFVGNGYKPSLSIKAPIDVLRNKMYLQ
NLQRMVERNRNFLKSY

>Corticotropin releasing factor like-DH 37 X2 (CRF-DH37)

MRLPIYLICAAFVLAIEAEENAVDYYGSFLEPMKISSEREPLNNYMLPKVSAKYRPEWVR
LPEPSYYMPEEEVNADADRLNNIAHETRIKRAEFMRPRGSLSIVNSLDALRNKLVMEISRK
KTQQNAERNRQFLKSFGKRTFSSHRSMDRFDNNI

>Corticotropin-releasing factor-like-diuretic hormone 44 (CRF-DH 44)

MTQYRDVAKVFAACWLIMAVRGLPHSGGQFEMGLKDERKLPKINSIQESNRRLSINKIPT
YLSSRFKRSSQHIVTECIFMTSEEGEFYHKATTVDGSACGIYIVAEPNQKVEVRFNYFDVP
CSNGGLVAFVDGWELNGELFPTPRDYPLPSNTRFREFCDEKKIKQIFVSSSNVALIQYRMP
YRGSGFTITVRFIKNPTPCNTLLQGFEEVYTLKNYEKRSNCSVSTLFPSAVRIEAINVGVVR
ADLRSMEMEIGTIHKCQKRGLEDYVQIGGSTGLDNSNLIIADSVCGLNSRPGRFEEFIACG
TTTVRLVSSGAFDNSVTVHLRQLDGEEDMNSFMSVLCPIEEIRK

>Eclosion hormone (EH)

MPRHLLMSKLMYVVLPVIVVYFVSANYIGICIRNCAQCKKMFGPYFEGQLCADACLSFK
GRIIPDCEDIFSVGPFLNEIHE

>Ecdysis triggering hormone (ETH)

MWFSNSLVLIALFLLCSLRFIECQDTTLDEFFLKASKNIPRIGRSGGSKGSSNDDFENFFLK
ASKSVPRIGRRNEIQQGRLVEESPNSYIYTEKSDIVPNVRKYPTWSEIAKMYENDHQGMV
TDGTNSNKLSNGNYEEFQNEKEYGRSKRTTNYV

>FMRFamide (FMRF)

MFIQTLLVLVVVHFTWGYNEDNYPYPDGFDANMNIPEDYDEQFNDFEPVEKRSHNFIRFG
RAPSEQEPEPRPARRNDYFVRFGRSKSDYLRFGRDMAHQRYGRSKDNYVRFGRSVPEVA
SGTRSKREATSEAETKRNSNYLRFGRGNSDFLRYGRSRDEQRMTGDLQMKLTPEDIKLLK
FQQLYDSPLIRLLAQLMSSDREKCKNNL

>Glycoprotein hormone alpha 2 (GPA2)

MLACWLVLGLLSISHAFFVSSVVAKNVWQQAGCHKVGHSRKISIPDCVEFNMTTNACRG
FCESWAVPSHPITNPLQPVTSVGQCCNMMESEPVEARVLCVDGLRTLTFKSALSCACYHC
KKD

>Glycoprotein hormone beta 5 (GPB5)

MKQLISLQIYIIVISTTVRSQSIIDVESLIPQEPETLSCEEMPYTFSVVQTDANGKQCWGAVT
TNACKGRCDSNEISDWRFPFKKSNHPVCVHYGRNRNVVTLRHCEEGALPEAARYEYLEA
VGCKCQICSSSDTSCEGITYRPHRSYPVSWGY

>Hansolin

MSMWTTSIFLVFLNIIFISSKPTRPIEGPLWIENRVLDDFAYAFRNNQPENYELFLPNKESY
KNVNFDEFLLRTPSHKRALTMFGRWGPINDLGKERSSIRSIDRVHPSTGRKLGQPFRWG

>IDLSRF-like peptide (IDLSRF)

MIRMASSPHPVLGAALGAIVIFAVCAALPTAVMAIDLSKLYGHMNSKRNGDACHPYEPF
KCPGDENCISIQYLCDGAPDCPDGYDEDSRLCTAAKRPPVEETGSFLKSLLASHGPNYLEK

LFGNKARDALKPLGGVDKVAIALSESQTIEDFGAALHLMRSDLEHLRSVFMAVENGDLG
MLKSLGIKDSELGDVKFFLEKLVNTGFLD
>Insulin-like peptide 1(ILP1)
MNISRLFFYLIIMFVSLCKCQMDSSEQRKKKYCGPNLSQTLSTVCRGNYNTLTRKTDAIEK
LGYRNENYDSDKEAAIYPLIRRSRAVSMRTNHARSKRGVFNECCQKACSQRELSYYCGH
>Insulin-like peptide 2(ILP2)
MVAAKLFIILTVLCTLVANGFSGESRLKKIHFCGTHLVNSMSLVCNKHYHSRNTKKSGNY
NQEFLDDNSIPEYFGNENQNALLPLHFFDRSFEDDINSYVPVHSSRRVKKGIYEECCENPC
TIDDLRAYCY
>Insulin-like peptide 3(ILP3)
MRLKLLITLAVFCILVNHCSSSETYRHYKRRFCGNKLVDAISLVCNKRYNSPSMKKSVDF
NQEYLDDNSVPDYFANENQNALSSFRFLDRSFEDDNGGYFPMAYARRMKKGIYEECCLN
PCTEDDLKAYCYIPRI
>Insulin-like peptide 4(ILP4)
MDTKIFIVSTIFCLLVGESSCNAYPRYKYCGTNLINALQSICKSQPHLRSKKSVDYHQGYS
NENSAVDNSERYGEYMLQPMESFGRLLTAFNSKHRTKRGIHDDCCLNGCTRDELISYCY
>Insulin-like peptide 5(ILP5)
MGRRLLALFYLYILALQYSSAREKYCGTYLVDTMRSVCRELRRTQSIGKRSVNINQRELE
LDQFFISDSSEDNNPQDSVYSQAFQRKRKGIVEECCFQSCKRETLMAYC
>Insulin-like peptide 7 (ILP7) /relaxin
MWLPFSAYAAVLCVLIDVYESTQADNDLELIFKQRSHSDWRDAWHKEKYVRCRETLIKH
LYWACENDIYRITRRRPTKRFDYTLKNDLEYPWIDEKNAKLFLRSRRNFGKRTGSITSECC
KSTGCTWEEYAEYCPTNKRYTSYV
>Ion transport peptide (ITP)
MNYLSSKVFTTLLWVCLALILFVSKSLASPADRSPSLVSHRVAKRSFFDIQCKGVYDRSIF
ARLDRICEDCYNLFREPQVHSLCRKNCFTTDYFKGCLDTLQLSDELEQIQTWIKQLHGAE
V
>ITG-like (ITG)
MKVLLSLLAICLVGQHKVDAWGGLFNRFSPEMLANMGYGGHGGFIQRGGGEGDEGILE
EYTNDVEDEPCYGKRCTANEHCCPGSVCVDVDGVIGSCIFAYGRRVGELCRRDTDCESG
LVCAEAEPGTLTRVCRPPVHQDKQYSEVCNMSNECDISRGLCCQLQRRHRQAPRKVCSY
FKDPLVCIGPVATDQIKSSVQHTAGEKRLTGFSNFKRPMH
>Myosuppressin (MS)
MQFQTIVSVLLGVLAVFMSSTASASVISCPPNNLQETSPYLRQLCYAIEQAISENIPTQEQY
NLGGNAKRQDVDHVFLRFGRRLGL
>Natalisin (NTL)
MAISMKWLFLLSLTTSLHAQDPRKRILLSPNLVEEIEEPNLFEDEPYVCSIHGCVKRSQSEV
GPFWANRGKRGPRYLQSKLYAQEPAWVYITDDDSGYNEPFFVTRGKKNLETSKKHPLLL
KGLLQDKRADSAPDNPFFAARGKKTADMY
>Neuroparsin-A (NP-A)
MQFNLSLVSFLAFVILLSYVQRCSACPRCKTTEECEAPPPTPCPYGEYINYCGRRACLKGP
GEKCGGPSMNSFGSCAHGLYCHKEGRCYGCFLPIFECYPQ
>Neuropeptide F 1a (NPF1a)

MRWALGIWWIVLVSAMVILEYKWAEAAPRLMRREDMVKELMKLDQYYSSIARPRYGKRAETVGSNFSPLDLDGQYQSDDNIDYSAVRR

>Neuropeptide F 1b (NPF1b)

MRWALGIWWIVLVSAMVILEYKWAEAAPRLMRREDMVKELMKLDQYYSSIARPSVRSAAPEASMSPKIQRAINMLRLQNLDRLYADRSRPRYGKRAETVGSNFSPLDLDGQYQSDDNIDYSAVRR

>Neuropeptide-like precursor 1 (NPLP1) X1

MAFHHFRLRFAIGFFLAILLMVQSDESCNLDFEKTISTLFAPHEPPSLQILTLRRYLLKILQNMIVKAEEIEGWNGNTYPKRNIEALARAGYLHTLPEDEEDNQTEIHNKRSLESLVKNGQLPAHHYTEEEGIKRSIESLARNGELKRDIQRMLEELYNDMTYAQDKRNLASIARDGGFAGKRNAAALLKNDRYLNRMLGQERDDKRNIASLKANYKPKYKREITKRQADYSGNELEYPVYQSSGDYENLVQELLEEQRNQKRFLGSIAKTGWYDSPRSSSIALSGQRSPPGKRHIGSLARLGWLPTVRNVRRFSRSGRSYSGSCRSEWMETPTDGQGKNYYISDNSVPLDDKRFLPVPAMWKVPLQTFNY

>Neuropeptide-like precursor 1 (NPLP1) X2

MNSQTISMMVFRIFIFTMLSAVQSDESCNLDFEKTISTLFAPHEPPSLQILTLRRYLLKILQNMIVKAEEIEGWNGNTYPKRNIEALARAGYLHTLPEDEEDNQTEIHNKRSLESLVKNGQLPAHHYTEEEGIKRSIESLARNGELKRDIQRMLEELYNDMTVAQDKRNLASIARDGGFAGKRNAAALLKNDRYLNRMLGQERDDKRNIASLKANYKPKYKREITKRQADYSGNELEYPVYQSSGDYENLVQELLEEQRNQKRFLGSIAKTGWYDSPRSSSIALSGQRSPPGKRHIGSLARLGWLPTVRNVRRFSRSGRSYSGSCRSEWMETPTDGQGKNYYISDNSVPLDDKRFLPVPAMWKVPLQTFNY

>Orcokinin A (OK A)

MLPTFALTILAVSAVTSTAIPRKGEYLRDLALFLQPAMERETASSLKKRTYGSLSLGGSYGTAKRGDLSLLRLGSSFGRLARSDIRLDDYGILPHGYDPLINSYSGNKYSPPRILEDTSVEDYPIGKNGRSRNSGDSFMKQFPSEEFNKED

>Orcokinin B (OK B)

MLPTFALTILAVSAVTSTAIPRKGEYLRDLALFYQPSPLLCNSLSNDYEILLNDIDNENEDKIYRTADNSMDLSYPDMNNLFEKKSLDRIGGAGLLGKRALDGIGGGALLGKRALDRIGGGALLGKRALDRIGGGALLGKRALDGIGGGALLGKRALDRIGGGALLGKRALDGIGGGALLGKRALDGIGGGALLGKRALDGIGGGALLGKRSYDNEPTMVRRALDSIGGGALLGKRALDSIGGGGLIGKRRLDNFDENIFTEREFEEDDGSRISQENDKNTSN

>Pigment-dispersing factor (PDF)

MRSITAEHPISLIQILALGVILGCALAYPSPADDYLISEKEYSSPGAHQLASWIASQMRPKEITVIKELPIIPYRLPLQGKRNSEVTNAIIGSEETQKMYRDGRK

>Prothoracicotropic Hormone (PTTH)

MKFQILMVTISSFNFVISEDAWRQTEAEKIDFSDYNEFADDVVNDIAVKDVFSRNEKRRFSGYKETPNAIMDTKKSKKEIYQYGRSSTPCSCFVKYALLELGFMYSPRKLLTTVCDSSKCGNFYQCNEKKYGITVMKRKTRIEKISSHSSTSSRALEYPWIPEVKYVTVACECMEM

>Proctolin (Pro)

MIEMMSIKAPMLLLLLVTVTILTITEKSEARYLLARGSNDRIDKLRELLKELLENEIEKEDQGDAPPRWHPESKLFYKRETPETKH

>Pyrokinin (PK)/Phermone biosynthesis activating neuropeptide (PBAN)

MERIVLVNCVAVLLFAILGRVVSNHYGFLELRKASDQKQYSPLYPWKHRQKREPIADDY
FDYSDLERQQLSSWIVAMNDASRRIPNRINRESSNNKMSFANERWMDIDLASRSPPFAPR
LGRRNSSPFIPRLGRDSNRLFSF
>RFLamide (RF)
MDWLWKFLVTYIGHVCFTTVFITPAYASLYEFLDGDVHIQDIGDSAEDQNIDNSIKMLVSI
LRQPWPPGMSPVVYLEDNVEDLWTDQNQNSRVIPESFEQVDRNIQDKRSKYYRKYPLKR
QNRYSRYDAENRYMCLPSKNDVYRLLIALHETKQGIREKTVNFCNRRRPAKTVFTNIRFL
GK
>Ryamide (RY)
MILNGLRSPVQMNVRGFLLFLACILVFLLGYNEALLTTRYGKKNINNEEIMPRTGKSSGSF
FVGSRYGKRMAWSPGEEMESSPVPCSIFEGMSCDYTGISNYYRCSLRRNPDDEDFAESN
>Short neuropeptide F (sNPF)
MRSSHKMTYLCGIIWVILIVATITTSAPTYGEMDNNIQELLDALYQQDLLNDRFSGVHQV
VRKAGRSPSLRLRFGRRSDPALSPGAAYLMAQQAQADEN
>SIFamide (SIF)
MMSSKSATLFIGVLFISLLLFNVGASYRKPPFNGSIFGKRGNTLEYDTAGKTLSAMCEIASE
ACQAWFPNQEKK
>Sulfakinin (SK)
MTKILTGIFLVVSVYFLIIHQFRNISDAAAFRNTNVERMERANSEKVPLKRSNSKLNLLDV
FLDDEDFDFNEKRQFDDYGHMRFGKRGEDHLDDYGHLRFGRLI
>Tachykinin (TK)
MRFLQKITYPLTLIIFLHLVRSEDHHKRAPSGFTGVRGKKSIAEDVKAPPFYEDDADEEGS
NNGGSPQLPVPASELQFPSGVGGMPNKRVPSVGFVGMRGKKPWEGRIAAIDNSGMPKRA
PNGFFGMRGKKSGDLDALAYDLEKRVNSGFFGMRGKKDDFPDDMNYWFDKRAPSMGF
VGMRGRKSFMDPSMDFDKRTPSGFFGMRGKKDWGAFGLRGKKIPYQFRGKFVGVRGK
KNQVISDDGTLEADPNYELNMNQLMMQLVEGEALDKTGADGV
>Trissin (TR)
MGRRWFIEIFFLVLIFLGAHSCNSCGSECESSCGTRHFRTCCFNYLRKRSNLFYTMSPENE
AIIKVKLRTHHPSFSMNYQQKALHN

Table S4. Overview of the presence of neuropeptide precursors in *H. vigintioctopunctata* and other insects.

Sequences of other insects are retrieved from the published neuropeptidomes of *Harmonia axyridis* (Haxy) (Veenstra, 2019), *Coccinella septempunctata* (Csep) (Han et al., 2024; Veenstra, 2019), *Tribolium castaneum* (Tcas) (Li et al., 2008; Veenstra, 2019), *Tenebrio molitor* (Tmol) (Marciniak et al., 2022; Veenstra, 2019), *Drosophila melanogaster* (Dmel) (Nässel & Zandawala, 2019), *Nilaparvata lugens* (Nlug) (Tanaka et al., 2014), *Apis mellifera* (Amel) (Hummon et al., 2006), *Bombyx mori* (Bmor) (Roller et al., 2008). ●, identified; ○, not identified.

**Orders**	**Coleoptera**					**Diptera**	**Hemiptera**	**Hymenoptera**	**Lepidoptera:**
**Species / Neuropeptides**	***Hvp***	***Haxy***	***Csep***	***Tcas***	***Tmol***	***Dmel***	***Nlug***	***Amel***	***Bmor***
ACP	○	○	○	●	●	○	●	○	●
Agatoxin-like	●	●	●	●	●	○	●	●	●
AKH	●	●	●	●	●	●	●	●	●
ALP	●	●	●	●	●	○	●	○	●
AstA	○	○	○	○	○	●	●	●	●
AstB	●	●	●	●	●	●	●	○	●
AstC	○	○	○	●	●	●	●	○	●
AstCC	●	●	●	●	●	●	●	●	●
AstCCC	●	●	●	●	●	●	●	●	○
AT	●	●	●	●	●	○	●	●	●
ADF	●	○	○	●	●	○	○	○	○
AVPL	●	●	●	●	●	○	●	○	○

Baratin	●	●	●	●	●	○	○	○	○
Bur α	●	●	●	●	●	●	●	●	●
Bur β	●	●	●	●	●	●	●	●	●
Calcitonin	○	●	●	●	●	○	○	○	○
CAP2b/CAPA/PVK	●	●	●	●	●	●	●	●	●
CCAP	●	●	●	●	●	●	●	●	●
CCH1	●	●	●	●	●	●	●	●	●
CCH2	●	●	●	●	●	●	●	●	●
Crz	○	○	○	○	○	●	●	●	●
CNM	●	●	●	●	●	●	●	●	○
CL-DH31	●	●	●	●	●	●	●	●	●
CRF-DH 34	○	○	○	○	○	○	○	○	●
CRF-DH 37	●	○	○	●	●	●	●	●	●
CRF-DH 44	●	○	○	○	○	●	○	●	●
CRF-DH 45	○	○	○	○	○	○	○	○	●
CRF-DH 47	○	●	●	●	●	○	○	○	○
EH	●	●	●	●	●	●	●	●	●
Elevenin	○	○	○	●	●	○	○	○	○
ETH	●	●	●	●	●	●	●	●	●
FMRF	●	●	●	●	●	●	●	●	●
GPA2	●	●	●	●	●	●	●	○	●
GPB5	●	●	●	●	●	●	●	○	●

Hansolin	●	●	●	●	●	○	○	○	○
IMF	○	○	○	○	●	○	○	○	●
IDLSRF	●	●	●	●	●	●	●	●	●
ILP	●	●	●	●	●	●	●	●	●
ITP	●	●	●	●	●	●	●	●	●
ITG	●	●	●	●	●	●	●	●	●
IGFLP	○	○	○	○	●	●	○	○	●
LK	○	○	○	○	○	●	●	●	●
MS	●	●	●	●	●	●	●	●	●
NTL	●	●	●	●	●	●	●	●	●
NP	●	●	●	●	●	○	●	●	●
NPF	●	●	●	●	●	●	●	○	●
NPLP 1	●	●	●	●	●	●	●	●	●
NPLP 2	○	○	○	○	○	●	○	●	○
NPLP 3	○	○	○	○	○	●	●	●	○
NPLP 4	○	○	○	○	○	●	●	○	○
OK	●	●	●	●	●	●	●	●	●
PDF	●	●	●	●	●	●	●	●	●
PTTH	●	●	●	●	●	●	●	○	●
Pro	●	●	●	●	●	●	●	○	●
PK/PBAN/DH	●	●	●	●	●	●	●	●	●
RF	●	●	●	●	●	○	○	○	○

RY	●	●	●	●	●	●	●	●	●
sNPF	●	●	●	●	●	●	●	●	●
SIF	●	●	●	●	●	●	●	●	●
SK	●	●	●	●	●	●	●	●	●
TK	●	●	●	●	●	●	●	●	●
TR	●	●	●	●	●	●	○	○	●

Table S5. G protein-coupled receptors for neuropeptide identified in *H. vigintioctopunctata*

Family	Neuropeptide Receptor	Likely ligands	GenBank Accession No.	Receptor size (aa)	Transmembrane domains (NO.)	Homology search with known protein (Blastp)			
						Species	E-value	ID	Accession No.
A-family (or rhodopsin-like)	Neuropeptide receptor A1	Allostatin-like	PV645181	421, complete	7	*Bombyx mori*	1.00 E-129	61.20 %	NP_001127736.1
	Neuropeptide receptor A2	AKH	PV645192	296, complete	6	*Harmonia axyridis*	2.00 E-154	82.88 %	XP_045482662.1
	Neuropeptide receptor A3	Allatotropin/ Orexin	PV645199	446, complete	7	*Tribolium madens*	9.00 E-172	56.09 %	XP_044259620.1
	Neuropeptide receptor A4	Allatotropin/ Orexin	PV645200	389, complete	6	*Coccinella septempunctata*	0	69.11 %	XP_044759416.1
	Neuropeptide receptor A5	CAPA-PVK	PV645201	418, complete	7	*Coccinella septempunctata*	0	66.67 %	XP_044749380.1
	Neuropeptide receptor A6	CCH1	PV645202	420, complete	7	*Coccinella septempunctata*	0	82.34 %	XP_044759703.1
	Neuropeptide receptor A7	CCH2	PV645203	416, complete	7	*Coccinella septempunctata*	0	77.11 %	XP_044758875.1
	Neuropeptide receptor A8	CCAP	PV645204	384, complete	7	*Tribolium castaneum*	0	69.39 %	NP_001076796.1
	Neuropeptide receptor A9	CCAP	PV640599	401, complete	7	*Tribolium castaneum*	0	81.46 %	NP_001076795.1

	Neuropeptide receptor A10	ETH	PV645182	497, complete	7	*Leptinotarsa decemlineata*	0	69.25%	QBH70335.1
	Neuropeptide receptor A11	ETH	PV645183	466, complete	6	*Leptinotarsa decemlineata*	0	71.70%	QBH70336.1
	Neuropeptide receptor A12	FMRF	PV645184	373, complete	6	*Harmonia axyridis*	0	83.97%	XP_045467883.1
	Neuropeptide receptor A13	insulin	PV645185	1393, complete	1	*Coccinella septempunctata*	0	73.45%	XP_044766317.1
	Neuropeptide receptor A14	insulin	PV645186	1272, complete	1	*Harmonia axyridis*	0	53.55%	XP_045479042.1
	Neuropeptide receptor A15	MS	PV645187	344, complete	3	*Coccinella septempunctata*	6.00E-179	76.88%	XP_044754225.1
	Neuropeptide receptor A16	NPF	PV645188	429, complete	7	*Agasicles hygrophila*	0	64.66%	WDW32492.1
	Neuropeptide receptor A17	pyrokinin	PV645189	649, complete	6	*Coccinella septempunctata*	0	69.74%	XP_044762323.1
	Neuropeptide receptor A18	pyrokinin	PV645190	535, complete	7	*Coccinella septempunctata*	0	78.28%	XP_044762323.1
	Neuropeptide receptor A19	RY	PV645191	421, complete	7	*Coccinella septempunctata*	0	76.79%	XP_044756801.1
	Neuropeptide receptor A20	SP/AstB	PV645193	404, complete	7	*Harmonia axyridis*	0	86.15%	XP_045464657.1

	Neuropeptide receptor A21	TK/ITPL	PV645194	249, complete	5	*Coccinella septempunctata*	7.00E-142	84.43%	XP_044761513.1
	Neuropeptide receptor A22	TK/ITPL	PV645195	437, complete	7	*Coccinella septempunctata*	0	86.30%	XP_044762535.1
	Neuropeptide receptor A23	TR	PV645196	325, complete	5	*Anoplophora glabripennis*	2.00E-143	64.16%	XP_023311047.1
	Neuropeptide receptor A24	SK	PV645197	253, complete	3	*Harmonia axyridis*	2.00E-96	59.50%	XP_045479675.1
	Neuropeptide receptor A25	Orphan (Neuropeptide FF, NPFF)	PV645198	365, complete	7	*Coccinella septempunctata*	3.00E-170	65.71%	XP_044746322.1
B-family (or secretin-like)	Neuropeptide receptor B1	CL-DH31	PV645205	383, complete	7	*Harmonia axyridis*	0	75.98%	XP_045474633.1
	Neuropeptide receptor B2	CRF-DH44	PV645206	400, complete	7	*Harmonia axyridis*	0	82.04%	XP_045474413.1
	Neuropeptide receptor B3	PDF	PV645207	425, complete	7	*Coccinella septempunctata*	0	83.46%	XP_044752499.1
	Neuropeptide receptor B4	Orphan (Parathyroid hormone like, PTH-like)	PV645208	425, complete	8	*Harmonia axyridis*	0	58.67%	XP_045463972.1
Leucine-rich repeat-containing GPCRs (LGRs)	Leucine-rich repeat G protein-coupled receptor 1	GPA2/GPB5	PV645176	801, complete	7	*Harmonia axyridis*	0	72.54%	XP_045463486.1
	Leucine-rich repeat G protein-	Bursicon	PV645177	1204, complete	6	*Tribolium castaneum*	0	79.21%	ABA40401.1

	coupled receptor 2								

Table S6. Primers in this study.

Gene	Direction	Sequence (5′-3′)	Primer efficiency	Gene	Direction	Sequence (5′-3′)	Primer efficiency
AKH2	F	TGCGGCTATCTGTTTTTGGA	94.45%	*ALP*	F	AATAACAGCCGAACAACTC	93.02%
	R	GTATCGACGAACCTCGTTTGC			R	GTCGTAACTTTCAGCACCA	
AstB	F	CCCCAACAACGATACGAG	94.31%	*AstCCx1*	F	ATACCCCTGAAACAACCCCG	83.87%
	R	GACAAGGTGGCTGTGAGC			R	GAGCTGCTAGTCCCAACTGA	
AstCCx2	F	ATACCCCTGAAACAACCCCG	80.94%	*AstCCC*	F	GACGCTTTGAGAAGACTGGC	117.39%
	R	TGAGCTGCTAGTCCCAACTG			R	GGTAACTTCTTCCCTGGCGT	
AT	F	ACTTTCAGTAGTTCCGTCTA	110.47%	*ADF-b1*	F	CTATTGCCGCTGGACCTCTT	116.23%
	R	TCCGTATCCTCGTGCT			R	CTAGGTGTCCGCCTAAACCG	
ADF-b4	F	GCTGGATTGCTTCCCCTAGT	92.28%	*ADF-b5*	F	GGAATCATTGCCCCAGGAGT	94.55%
	R	ATCCATGACCTCTTGGCACC			R	CGAGACCACCATGACCAACA	
AVPL	F	CACACACCTCCCTCTGGTTG	111.14%	*Baratin*	F	GAAAGATGGAGCACCAACGC	107.46%
	R	TGCATTGGGTGGCCCTTAAA			R	CGGTTTGAGTTCTCTGGCCT	
Bur-α	F	GGCTGCGTACCTAAACCCAT	94.18%	*Bur-β*	F	CGAAACTTGCGAAACCCTCC	95.75%
	R	TCGCCAGGTTTTGCTTTTGG			R	CCTGTGGGCGTAATAACCGA	
CAPA	F	GGTCGTGGTAAGGAATCCGC	106.95%	*CCAP*	F	GAACTCAATGCCGAACCAGC	109.87%
	R	AGAGTGCGGTATTGGAGTCG			R	AGCTGGCGATTTAGCCTGAG	
CCH1	F	TGCGGCTGTACCCGTTATTT	80.50%	*CCH2*	F	TGGGCCTAGAAGTGGAGGTT	112.32%
	R	AGTCAACCTGAAGACAGCGG			R	ACGACTGGGGACTTTTGTGG	
CNMa	F	TGGACGAAGAAATGCACGAC	85.58%	*CL-DH31*	F	AGTCTCTCCGCTTCCAAGTC	88.62%
	R	AGGCATCAGTAGGGTGTTGA			R	TGAAAATCCACGTCCAACGC	
CRF-[illegible]	F	AACCTGCAACGGATGGTAGA	99.93%	*CRF-*[illegible]	F	ACCCAACAAAATGCCGAACG	100.28%
	R	GGTGTTGTGAATGCTCGTCTC			R	CACTCATCGCCCAACTCTGT	
CRF-DH44	F	GCAAAAGTTCCTGCCACCTG	93.66%	*EH*	F	TACAATTATGCCCCGCCACT	86.48%
	R	ATTTCGAATTGTCCCCCGCT			R	GGACCAAACATCTTCTTGCACT	
ETH	F	GCATGGTAACTGACGGAACG	91.23%	*FMRF*	F	TGAACCAAGACCTGCACGAA	110.61%
	R	TTTGTGGTTCGCTTGGAACG			R	GCACACTCCTCCCGAATCTC	
GPA2	F	GTTGGGCTGTACCGTCTCAT	114.76%	*GPB5*	F	CGATGTCGAGTCTCTGATTCCA	108.116%
	R	GTCCACACAAAGCACACGAG			R	GGAATCGCATCTTCCCTTGC	
Hansolin	F	CGCACAAACGAGCTTTGACA	95.86%	*IDLSRF*	F	GTACGGCCACATGAACTCCA	99.64%
	R	TTGAAGGATGGACCCTGTCG			R	GGGGCACCATCACACAAGTA	

ILP1	F	AGTTCCGAAGACCTATT	110.21%	*ILP2*	F	TTGATCACGCTGGCTGTTTTC	83.92%
	R	TATGCGTATCTACCATT			R	TGGCACGGAGTTATCATCCAA	
ILP3	F	CAAAACGCCTTGTTACCTTTGC	94.35%	*ILP4*	F	TGGCCTTACAGTATTCCTCAGC	93.02%
	R	ATCGTGCATGGGTTTTCACAG			R	TCTTGTGGGTTGTTATCTTCCGA	
ILP5	F	GCCTTACAGTATTCCTC	120.00%	*ILP7*	F	CTGCTTATGCTGCTGTACTTTGT	89.06%
	R	TTGTGGGTTGTTATCTT			R	AATCGTTTTGTAGGTCTTCGTCT	
ITP	F	GGTGAACGGGTGTTGGTAGA	111.24%	*ITG*	F	TTGTAGTTGTGGCGCGTAGT	87.05%
	R	GCTGGACTTGCCAGAGACTT			R	ACCCCATGTTCTGTCGCATT	
Ms	F	CTCTTGGGAGTGTTGGCTGT	99.21%	*NTL*	F	ATCTCAGTCAGAAGTAGGTCCAT	99.92%
	R	GCATAGCACAGTTGTCGCAG			R	TGCCCTCTTGTCTTGAAGTAATC	
NPA	F	AGGTCCAGACATTGCCGAAC	113.12%	*NPF1a*		GTGGGCGCTAGGAATTTGGT	92.04%
	R	TGGACATGCCGAACACCTTT				ACCGTTTCAGCTCGTTTTCC	
NPF1b	F	CACGACCTAGTGTGAGGAGC	92.41%	*NPLP1-*<br>---	F	TCGTCTATATGCGCCGCTTT	92.3%
	R	TACCTGGGCCTGGACCTATC			R	TCCGATAGCGAAGCGAAGTC	
NPLP1-X2	F	ACTGACGCAACTGGTGGTTA	86.66%	*OKA*	F	CAATGCTGCCTACTTTCGCC	109.85%
	R	ACCGCAGACAGCATAGTGAA			R	TCTCTTTCCATGGCCGGTTG	
OKB	F	AGTCACATCCACCGCAATCC	97.43%	*PDF*	F	TCCTGGCGTTGGGAGTTATT	97.28%
	R	CATCCAAAGCCCGTTTGCC			R	GGTCGCATTTGGGAAGCAAT	
PTTH		TATCAGAGGATGCTTGGCGG	94.45%	*Pro*	F	ACCAAGATGGCACCCTGAAA	91.73%
		GGTGTTTCTTTGTAACCGCTGA			R	TCGTTTCCGTTTCATAGCCG	
PBAN-like	F	TGCTTCAAGATCTCCACCGT	81.07%	*RF*	F	ATACGCTTCACTTTACGAG	108.40%
	R	GCTATCTCTTCCTAGGCGTGG			R	TTGAATAACGGTTCTGTCT	
RY	F	CCTTCTTTGTAGGGTCA	82.52%	*sNPF*	F	CGCTTTACCAGCAGGACCTA	90.41%
	R	AGCATCGGTAGTAGTTAG			R	CGTCCGAAACGCAATCTCAA	
SIF	F	ACGTTGGTGCATCCTACAGAA	99.34%	*SK*	F	ATGTTGAAAGAATGGAACGTGC<br>–	99.92%
	R	TTGATTCGGGAACCATGCCT			R	AAGTGGCCGTAATCGTCCAA	
TK	F	AAGACGACGCTGACGAAG	92.00%	*TR*	F	TCATCATGTGGAACACGCCA	118.22%
	R	AGAATCCCGAGTTGACCC			R	GGCTTCATTTTCAGGAGACATCG	
RPL13	F	AGCATCCTTCGCTCGTTTAG	91.10%	*RPS18*	F	CGCAATCAAAGGTGTTGGAAG	84.24%
	R	TTCGACAACCTGCCATTAGG			R	GCCTAGGGTTGGCCATAATAG	

Table S7. GenBank accession numbers used for the multiple sequence alignments and phylogenetic analysis

Insect	Name	Acc. number	Insect	Name	Acc. number	Insect	Name	Acc. number
Henosepilachna vigintioctopunctata	Hvp_AKH	PV645140	*Tribolium_castaneum*	ILP2	XP_001814181.1	*Bombyx mori*	CCAP receptor	NP_001127724.1
Coccinella_septempunctata	AKH	XP_044765111.1	*Henosepilachna vigintioctopunctata*	Hvp_ILP3_like	PV645170	*Harmonia axyridis*	CCAP receptor	XP_045475807.1
Asbolus_verrucosus	AKH	RZB41086.1	*Coccinella_axyridis*	ILP1_Like	XP_045479779.1	*Coccinella septempunctata*	CCAP receptor	XP_044759447.1
Tribolium_castaneum	AKH2_precursor	NP_001107818.1	*Asbolus_verrucosus*	ILP3_Like	RZC34625.1	*Tribolium castaneum*	CCAP receptor	NP_001076796.1
Tenebrio_molitor	AKH1	UXO98184.1	*Henosepilachna vigintioctopunctata*	Hvp_ILP4_like	PV645171	*Drosophila melanogaster*	CCAP receptor	NP_996297.3
Bombyx_mori	AKH2_precursor	NP_001124365.1	*Camponotus_floridanus*	ILP3_Like	XP_011265971.1	*Henosepilachna vigintioctopunctata*	CCAP receptor (Hvp_A9)	PV640599
Henosepilachna vigintioctopunctata	Agatoxin-like (Agl)	PV645141	*Henosepilachna vigintioctopunctata*	Hvp_ILP5_like	PV645172	*Bombyx mori*	CCAP receptor	NP_001127724.1
Henosepilachna vigintioctopunctata	Hvp_AstB	PV645142	*Anoplophora_glabripennis*	ILP5_Like	XP_018572613.1	*Coccinella septempunctata*	CCAP receptor	XP_044752355.1
Coccinella_axyridis	AstB	XP_045479904.1	*Henosepilachna vigintioctopunctata*	Hvp_ILP7	PV645173	*Harmonia axyridis*	CCAP receptor	XP_045472538.1
Coccinella_septempunctata	AstB	XP_044748648.1	*Coccinella_septempunctata*	ILP7	XP_044762237.1	*Tribolium madens*	CCAP receptor	XP_044267776.1
Asbolus_verrucosus	AstB	RZC34805.1	*Coccinella_axyridis*	ILP7	XP_045480274.1	*Drosophila melanogaster*	CCAP receptor	AAO66429.1
Tribolium_castaneum	AstB	NP_001137202.1	*Tribolium_castaneum*	ILP7	XP_064212535.1	*Henosepilachna vigintioctopunctata*	ETH receptor (Hvp_A10)	PV645182
Henosepilachna vigintioctopunctata	Hvp_AstCC_X1	PV645143	*Drosophila_melanogaster*	ILP7	NP_570070.1	*Bombyx mori*	ETH receptor	NP_001127741.1
Henosepilachna vigintioctopunctata	Hvp_AstCC_X2	PV645144	*Henosepilachna vigintioctopunctata*	Hvp_ITP	PV645174	*Harmonia axyridis*	ETH receptor	XP_045473564.1
Coccinella_septempunctata	AstCC	XP_044759479.1	*Coccinella_septempunctata*	ITP	XP_044746445.1	*Coccinella septempunctata*	ETH receptor	XP_044753664.1
Coccinella_axyridis	AstCC	XP_045476886.1	*Coccinella_axyridis*	ITP	XP_045478547.1	*Tribolium castaneum*	ETH receptor	NP_001076792.1
Tribolium_castaneum	AstCC	XP_001810067.1	*Asbolus_verrucosus*	ITP	RZC33292.1	*Drosophila melanogaster*	ETH receptor	NP_001287439.1
Drosophila_melanogaster	AstCC	NP_609483.2	*Tribolium_castaneum*	ITP	NP_001076808.1	*Henosepilachna vigintioctopunctata*	ETH receptor (Hvp_A11)	PV645183
Henosepilachna vigintioctopunctata	Hvp_AstCCC	PV645145	*Drosophila_melanogaster*	ITP	NP_001163293.1	*Bombyx mori*	ETH receptor	NP_001165737.1
Coccinella_axyridis	AstCCC	XP_045476394.1	*Henosepilachna vigintioctopunctata*	Hvp_ITG	PV645175	*Harmonia axyridis*	ETH receptor	XP_045473566.1
Coccinella_septempunctata	AstCCC	XP_044759342.1	*Coccinella_axyridis*	ITG	XP_045481008.1	*Coccinella septempunctata*	ETH receptor	XP_044753665.1
Henosepilachna vigintioctopunctata	Hvp_AT	PV645146	*Coccinella_septempunctata*	ITG	XP_044744945.1	*Leptinotarsa decemlineata*	ETH receptor	QBH70336.1
Coccinella_septempunctata	AT	XP_044762303.1	*Zophobas_morio*	ITG	XP_063918923.1	*Tribolium castaneum*	ETH receptor	NP_001076793.1
Coccinella_axyridis	AT	XP_045463036.1	*Bombyx_mori*	ITG	NP_001124360.1	*Drosophila melanogaster*	ETH receptor	NP_001287439.1
Tribolium_castaneum	AT	NP_001137204.1	*Henosepilachna vigintioctopunctata*	Hvp_MS	PV645178	*Henosepilachna vigintioctopunctata*	FMRF receptor (Hvp_A12)	PV645184
Tenebrio_molitor	AT	KAJ3635763.1	*Anoplophora_glabripennis*	MS	XP_018573133.1	*Bombyx mori*	FMRF receptor	NP_001037007.1
Henosepilachna vigintioctopunctata	Hvp_ADF_b1_like	PV645147	*Diorhabda_carinulata*	MS	XP_057664360.1	*Harmonia axyridis*	FMRF receptor	XP_045467883.1
Coccinella_axyridis	ADF_B1_Like	XP_045474048.1	*Sitophilus_oryzae*	MS	XP_030748540.1	*Coccinella septempunctata*	FMRF receptor	XP_044757037.1
Tribolium_castaneum	ADFb1_Like	NP_001164303.1	*Henosepilachna vigintioctopunctata*	Hvp_NTL	PV645179	*Tribolium castaneum*	FMRF receptor	NP_001280540.1
Tribolium_madens	ADF_B1_Like	XP_044264818.1	*Rhynchophorus_ferrugineus*	NTL	QGA72564.1	*Drosophila melanogaster*	FMRF receptor	NP_001261347.1
Henosepilachna vigintioctopunctata	Hvp_ADF_b4_like	PV645148	*Coccinella_axyridis*	NTL	XP_045482586.1	*Henosepilachna vigintioctopunctata*	insulin-like receptor (Hvp_A13)	PV645185
Coccinella_septempunctata	ADF_B4_Like	XP_044747255.1	*Henosepilachna vigintioctopunctata*	Hvp_Neuroparsin_A	PV645180	*Bombyx mori*	insulin-like receptor	XP_062524422.1
Coccinella_axyridis	ADF_B4_Like	XP_045473967.1	*Coccinella_axyridis*	Neuroparsin_A	XP_045460883.1	*Coccinella septempunctata*	insulin-like receptor	XP_044766317.1
Tribolium_castaneum	ADFb4_Like_protein	EFA07533.2	*Coccinella_septempunctata*	Neuroparsin_A	XP_044745620.1	*Harmonia axyridis*	insulin-like receptor	XP_045482514.1
Diabrotica_virgifera_virgifera	ADF_B4_Like	XP_028148543.1	*Tenebrio_molitor*	Neuroparsin_A	UXO98153.1	*Tribolium castaneum*	insulin-like receptor	XP_008199415.2
Henosepilachna vigintioctopunctata	Hvp_ADF_b5_like	PV645149	*Tribolium_castaneum*	neuroparsin_A	XP_015836074.1	*Drosophila melanogaster*	insulin-like receptor	CAY93028.1
Coccinella_axyridis	ADF_B5_Like	XP_045474033.1	*Henosepilachna vigintioctopunctata*	Hvp_NPF1a	PV645211	*Henosepilachna vigintioctopunctata*	insulin receptor (Hvp_A14)	PV645186
Anthonomus_grandis_grandis	ADF_B5_Like	XP_050294704.1	*Coccinella_septempunctata*	NPF1a	XP_044761647.1	*Bombyx mori*	insulin receptor	XP_062524422.1
Henosepilachna vigintioctopunctata	Hvp_AVPL	PV645150	*Tenebrio_molitor*	NPF1a	UXO98177.1	*Harmonia axyridis*	insulin receptor	XP_045479042.1
Coccinella_septempunctata	AVPL	XP_044759105.1	*Bombyx_mori*	NPF1a	NP_001124355.1	*Coccinella septempunctata*	insulin receptor	XP_044749880.1
Coccinella_axyridis	AVPL	XP_045478516.1	*Henosepilachna vigintioctopunctata*	Hvp_NPF1b	PV645212	*Lasioderma serricorne*	insulin receptor	UVJ47537.1
Tribolium_castaneum	AVPL	NP_001078831.1	*Coccinella_septempunctata*	NPF1b	XP_044761646.1	*Drosophila melanogaster*	insulin receptor	AAC47458.1
Onthophagus_taurus	AVPL_Like	XP_022916575.1	*Coccinella_axyridis*	NPF1b	XP_045460796.1	*Henosepilachna vigintioctopunctata*	MS receptor (Hvp_A15)	PV645187

Henosepilachna vigintioctopunctata	Hvp_Baratin(NVP_like)	PV645151	*Bombyx_mori*	NPF1b_precursor	NP_001166883.1	*Bombyx mori*	MS receptor	XP_012549364.2
Coccinella_septempunctata	NVP_Like	XP_044759112.1	*Henosepilachna vigintioctopunctata*	Hvp_NPLP1_X1	PV645209	*Coccinella septempunctata*	MS receptor	XP_044754225.1
Coccinella_axyridis	NVP_Like	XP_045476989.1	*Henosepilachna vigintioctopunctata*	Hvp_NPLP1_X2	PV645210	*Dendroctonus ponderosae*	MS receptor	XP_019756536.1
Tribolium_castaneum	NVP_Like_protein	EFA09163.1	*Coccinella_septempunctata*	NPLP1	XP_044759118.1	*Drosophila melanogaster*	MS receptor	NP_647713.2
Tenebrio_molitor	NVP_Like_protein	KAJ3624825.1	*Coccinella_axyridis*	NPLP1	XP_045477560.1	*Henosepilachna vigintioctopunctata*	NPF receptor (Hvp_A16)	PV645188
Henosepilachna vigintioctopunctata	Hvp_bursicon_alpha	PP430623	*Tribolium_madens*	NPLP1	XP_044252396.1	*Bombyx mori*	NPF receptor	NP_001127708.1
Coccinella_septempunctata	bursicon_alpha	XP_044751157.1	*Henosepilachna vigintioctopunctata*	Hvp_Orcokinin_A	PV645213	*Coccinella septempunctata*	NPF receptor	XP_044763002.1
Tribolium_castaneum	bursicon_alpha	ABA40402.1	*Coccinella_axyridis*	Orcokinin_A	XP_045477291.1	*Harmonia axyridis*	NPF receptor	XP_045464333.1
Aethina_tumida	bursicon_alpha	XP_019870760.1	*Coccinella_septempunctata*	Orcokinin_A	XP_044759013.1	*Drosophila melanogaster*	NPF receptor	NP_001262086.1
Bombyx_mori	bursicon_alpha_precursor	NP_001091845.1	*Henosepilachna vigintioctopunctata*	Hvp_Orcokinin_B	PV645214	*Henosepilachna vigintioctopunctata*	PK1 receptor (Hvp_A17)	PV645189
Drosophila_melanogaster	bursicon_alpha	NP_650983.1	*Coccinella_axyridis*	Orcokinin_B	XP_045477290.1	*Bombyx mori*	PK1 receptor	NP_001036913.1
Henosepilachna vigintioctopunctata	Hvp_bursicon_beta	PP430624	*Coccinella_septempunctata*	Orcokinin_B	XP_044759011.1	*Coccinella septempunctata*	PK1 receptor	XP_044762323.1
Beta_Dendroctonus_ponderosae	bursicon	XP_019755392.1	*Henosepilachna vigintioctopunctata*	Hvp_PDF	PV645215	*Harmonia axyridis*	PK1 receptor	XP_045483658.1
Leptinotarsa_decemlineata	bursicon_Beta	XP_023014169.1	*Coccinella_septempunctata*	PDF	XP_044754547.1	*Zophobas morio*	PK1 receptor	XP_063912848.1
Beta_Sitophilus_oryzae	bursicon	XP_030754854.1	*Coccinella_axyridis*	PDF	XP_045474172.1	*Drosophila melanogaster*	PK1 receptor	NP_001014620.1
Bombyx_mori	bursicon_Beta	NP_001037289.1	*Henosepilachna vigintioctopunctata*	Hvp_PTTH	PV645217	*Henosepilachna vigintioctopunctata*	PK1 receptor (Hvp_A18)	PV645190
Drosophila_melanogaster	bursicon_Beta	NP_609712.1	*Coccinella_septempunctata*	PTTH	XP_044757586.1	*Bombyx mori*	PK1 receptor	XP_037870196.1
Henosepilachna vigintioctopunctata	Hvp_CAPA	PV645153	*Henosepilachna vigintioctopunctata*	Hvp_Proctolin	PV645216	*Coccinella septempunctata*	PK1 receptor	XP_044762323.1
Coccinella_axyridis	CAPA	XP_045479553.1	*Coccinella_septempunctata*	Proctolin	XP_044763568.1	*Harmonia axyridis*	PK1 receptor	XP_045483658.1
Tenebrio_molitor	CAPA	UXO98159.1	*Tribolium_madens*	Proctolin	XP_044260703.1	*Aethina tumida*	PK1 receptor	XP_049821737.1
Tribolium_madens	CAPA	XP_044255847.1	*Tribolium_castaneum*	Proctolin	XP_008190594.1	*Aethina tumida*	PK1 receptor	XP_049821737.1
Henosepilachna vigintioctopunctata	Hvp_CCAP	PV645160	*Henosepilachna vigintioctopunctata*	Pyrokinin/PBAN_like	PV645218	*Henosepilachna vigintioctopunctata*	RY receptor (Hvp_A19)	PV645191
Coccinella_axyridis	CCAP	XP_045465746.1	*Coccinella_axyridis*	Pyrokinin/PBAN_Like	XP_045482011.1	*Bombyx mori*	RY receptor	NP_001127717.1
Coccinella_septempunctata	CCAP	XP_044757276.1	*Coccinella_septempunctata*	Pyrokinin/PBAN_Like	XP_044765783.1	*Coccinella septempunctata*	RY receptor	XP_044756801.1
Tribolium_castaneum	CCAP	XP_008201234.1	*Tribolium_castaneum*	Pyrokinin/PBAN_Like	XP_015835244.1	*Harmonia axyridis*	RY receptor	XP_045468643.1
Bombyx_mori	CCAP	NP_001124369.1	*Henosepilachna vigintioctopunctata*	Hvp_RFLamide	PV645219	*Tribolium madens*	RY receptor	XP_044259449.1
Drosophila_melanogaster	CCAP	NP_001262846.1	*Coccinella_septempunctata*	RFLamide	XP_044750966.1	*Drosophila melanogaster*	RY receptor	NP_524525.3
Henosepilachna vigintioctopunctata	HVP_CCH1	PV645154	*Coccinella_axyridis*	RFLamide	XP_045470659.1	*Henosepilachna vigintioctopunctata*	SEP receptor (Hvp_A20)	PV645193
Coccinella_septempunctata	CCH1	XP_044761667.1	*Tenebrio_molitor*	RFLamide	UXO98182.1	*Bombyx mori*	SEP receptor	NP_001108346.1
Coccinella_axyridis	CCH1	XP_045461422.1	*Henosepilachna vigintioctopunctata*	Hvp_RY	PV645220	*Harmonia axyridis*	SEP receptor	XP_045464657.1
Tenebrio_molitor	CCH1	UXO98161.1	*Coccinella_septempunctata*	RY	XP_044747600.1	*Zophobas morio*	SEP receptor	XP_063920165.1
Henosepilachna vigintioctopunctata	Hvp_CCH2	PV645155	*Coccinella_axyridis*	RY	XP_045483580.1	*Tribolium castaneum*	SEP receptor	NP_001106940.1
Tenebrio_molitor	CCH2	UXO98162.1	*Tribolium_castaneum*	RY	NP_001280530.1	*Drosophila melanogaster*	SEP receptor	NP_001284892.1
Tribolium_castaneum	CCH2	XP_008190391.1	*Henosepilachna vigintioctopunctata*	Hvp_sNPF	PV645221	*Henosepilachna vigintioctopunctata*	TK-86C receptor (Hvp_A21)	PV645194
Coccinella_axyridis	CCH2	XP_045479368.1	*Coccinella_axyridis*	sNPF	XP_045470701.1	*Bombyx mori*	TK-86C receptor	NP_001127748.1
Coccinella_septempunctata	CCH2	XP_044748740.1	*Coccinella_septempunctata*	sNPF	XP_044761630.1	*Harmonia axyridis*	TK-86C receptor	XP_045460022.1
Henosepilachna vigintioctopunctata	Hvp_CNMamide	PV645156	*Tribolium_castaneum*	sNPF	XP_008198705.1	*Coccinella septempunctata*	TK-86C receptor	XP_044761513.1
Coccinella_axyridis	CNMamide	XP_045461995.1	*Henosepilachna vigintioctopunctata*	Hvp_SIF	PV645222	*Aethina tumida*	TK-86C receptor	XP_049820597.1
Tenebrio_molitor	CNMamide	UXO98163.1	*Coccinella_septempunctata*	SIF	XP_044744559.1	*Drosophila melanogaster*	TK-86C receptor	NP_524304.2
Tribolium_castaneum	CNMamide	KYB28527.1	*Aethina_tumida*	SIF	XP_019881334.1	*Henosepilachna vigintioctopunctata*	TK-99D receptor (Hvp_A22)	PV645195
Henosepilachna vigintioctopunctata	Hvp_CL_DH31	PV645152	*Tribolium_castaneum*	SIF	XP_001814498.1	*Bombyx mori*	TK-99D receptor	XP_021206901.2
Harpegnathos_saltator	CL_DH31	XP_025160820.1	*Bombyx_mori*	SIF	NP_001124358.1	*Coccinella septempunctata*	TK-99D receptor	XP_044762535.1
Lygus_hesperus	CL_DH31	APB88057.1	*Drosophila_melanogaster*	SIF	NP_001246496.1	*Harmonia axyridis*	TK-99D receptor	XP_045475839.1
Rhodnius_prolixus	CL_DH31	ACX47068.1	*Henosepilachna vigintioctopunctata*	Hvp_SK	PV645223	*Tribolium madens*	TK-99D receptor	XP_044261789.1
Bombyx_mori	CL_DH31_isoform_X2	XP_062531097.1	*Coccinella_septempunctata*	SK	XP_044752147.1	*Drosophila melanogaster*	TK-99D receptor	NP_001163772.1
Drosophila_melanogaster	CL_DH31_isoform_C	NP_723401.1	*Tribolium_madens*	SK	XP_044268639.1	*Henosepilachna vigintioctopunctata*	TR receptor (Hvp_A23)	PV645196
Henosepilachna vigintioctopunctata	Hvp_CRF_DH44	PV645159	*Tribolium_castaneum*	SK	XP_008194373.1	*Bombyx mori*	TR receptor	NP_001127747.1
Coccinella_axyridis	CRF_DH44	XP_045483757.1	*Henosepilachna vigintioctopunctata*	Hvp_TK	PV645224	*Anoplophora glabripennis*	TR receptor	XP_023311047.1

Coccinella_septempunctata	CRF_DH44	XP_044762180.1	*Coccinella_septempunctata*	TK	XP_044759920.1	*Aethina tumida*	TR receptor	XP_049822579.1
Zophobas_morio	CRF_DH44	XP_063909679.1	*Coccinella_axyridis*	TK	XP_045478364.1	*Tribolium castaneum*	TR receptor	XP_015836095.1
Tribolium_castaneum	CRF_DH44	XP_008201043.1	*Tribolium_castaneum*	TK	XP_015838251.1	*Drosophila melanogaster*	TR receptor	NP_001285641.1
Bombyx_mori	CRF_DH44	XP_004927514.4	*Henosepilachna vigintioctopunctata*	Hvp_TR	PV645225	*Henosepilachna vigintioctopunctata*	SK receptor (Hvp_A24)	PV645197
Henosepilachna vigintioctopunctata	Hvp_CRF_DH37_X1	PV645157	*Anoplophora_glabripennis*	TR	XP_018571856.1	*Bombyx mori*	SK receptor	NP_001127744.1
Henosepilachna vigintioctopunctata	Hvp_CRF_DH37_X2	PV645158	*Dendroctonus_ponderosae*	TR	XP_019766470.2	*Harmonia axyridis*	SK receptor	XP_045479675.1
Coccinella_septempunctata	CRF_DH37	XP_044750145.1	*Drosophila_melanogaster*	TR	NP_650471.2	*Coccinella septempunctata*	SK receptor	XP_044749522.1
Coccinella_axyridis	CRF_DH37	XP_045469784.1	*Henosepilachna vigintioctopunctata*	Allostatin-like receptor (Hvp_A1)	PV645181	*Diorhabda carinulata*	SK receptor	XP_057662901.1
Zophobas_atratus	CRF_DH37	UXO98074.1	*Bombyx mori*	Allostatin-like receptor	NP_001127736.1	*Drosophila melanogaster*	SK receptor	NP_001097021.1
Tribolium_castaneum	CRF_DH37	NP_001164096.1	*Coccinella septempunctata*	Allostatin-like receptor	XP_044754324.1	*Henosepilachna vigintioctopunctata*	NPFF receptor (Hvp_A25)	PV645198
Henosepilachna vigintioctopunctata	Hvp_EH	PV645162	*Tribolium castaneum*	Allostatin-like receptor	NP_001280521.1	*Bombyx mori*	Neuropeptide FF, NPFF receptor	NP_001127751.1
Aethina_tumida	EH	XP_019872488.1	*Tribolium madens*	Allostatin-like receptor	XP_044272062.1	*Coccinella septempunctata*	Neuropeptide FF, NPFF receptor	XP_044746322.1
Halyomorpha_halys	EH	XP_024214295.1	*Drosophila melanogaster*	Allostatin-like receptor	AAL02125.1	*Harmonia axyridis*	Neuropeptide FF, NPFF receptor	XP_045483282.1
Tribolium_castaneum	EH	XP_015838085.1	*Henosepilachna vigintioctopunctata*	AKH receptor (Hvp_A2)	PV645192	*Tribolium castaneum*	Neuropeptide FF, NPFF receptor	XP_972230.2
Bombyx_mori	EH	NP_001037307.1	*Bombyx mori*	AKH receptor	XP_012546718.1	*Henosepilachna vigintioctopunctata*	CL-DH31 receptor (Hvp_B1)	PV645205
Drosophila_melanogaster	EH	NP_001262668.1	*Coccinella septempunctata*	AKH receptor	XP_044747125.1	*Bombyx mori*	CL-DH31 receptor	XP_062525503.1
Henosepilachna vigintioctopunctata	Hvp_ETH	PV645161	*Harmonia axyridis*	AKH receptor	XP_045482662.1	*Harmonia axyridis*	CL-DH31 receptor	XP_045474633.1
Coccinella_axyridis	ETH	XP_045481070.1	*Tribolium castaneum*	AKH receptor	NP_001076809.1	*Coccinella septempunctata*	CL-DH31 receptor	XP_044755229.1
Coccinella_septempunctata	ETH	XP_044746230.1	*Drosophila melanogaster*	AKH receptor	NP_001260149.1	*Zophobas morio*	CL-DH31 receptor	XP_063926057.1
Aethina_tumida	ETH	XP_019877401.1	*Henosepilachna vigintioctopunctata*	Allatotropin/Orexin receptor type 1 (Hvp_A3)	PV645199	*Drosophila melanogaster*	CL-DH31 receptor	NP_725278.1
Tribolium_castaneum	ETH	EFA07492.2	*Tribolium madens*	Allatotropin/Orexin receptor type 1	XP_044259620.1	*Henosepilachna vigintioctopunctata*	CRF-DH44 receptor (Hvp_B2)	PV645206
Henosepilachna vigintioctopunctata	Hvp_FMRF	PV645163	*Tribolium castaneum*	Allatotropin/Orexin receptor type 1	XP_967232.2	*Bombyx mori*	CRF-DH44 receptor	XP_021208420.1
Coccinella_axyridis	FMRF	XP_045478717.1	*Leptinotarsa decemlineata*	Allatotropin/Orexin receptor type 1	XP_023018238.1	*Harmonia axyridis*	CRF-DH44 receptor	XP_045474413.1
Coccinella_septempunctata	FMRF	XP_044749142.1	*Drosophila melanogaster*	Allatotropin/Orexin receptor type 1	AAP69822.1	*Coccinella septempunctata*	CRF-DH44 receptor	XP_044755210.1
Aethina_tumida	FMRF	XP_019869081.1	*Henosepilachna vigintioctopunctata*	Allatotropin/Orexin receptor type 2 (Hvp_A4)	PV645200	*Tribolium castaneum*	CRF-DH44 receptor	XP_008198347.1
Tribolium_castaneum	FMRF	EFA02863.1	*Bombyx mori*	Allatotropin/Orexin receptor type 2	NP_001127750.1	*Drosophila melanogaster*	CRF-DH44 receptor	NP_610960.1
Henosepilachna vigintioctopunctata	Hvp_GPA2	PV645164	*Coccinella septempunctata*	Allatotropin/Orexin receptor type 2	XP_044759416.1	*Drosophila melanogaster*	CRF-DH44 receptor	NP_725175.3
Tribolium_castaneum	GPA2_precursor	NP_001164244.1	*Harmonia axyridis*	Allatotropin/Orexin receptor type 2	XP_045476198.1	*Henosepilachna vigintioctopunctata*	PDF receptor (Hvp_B3)	PV645207
Tenebrio_molitor	GPA2	UXO98142.1	*Drosophila melanogaster*	Allatotropin/Orexin receptor type 2	ABE73274.1	*Bombyx mori*	PDF receptor	XP_062524737.1
Asbolus_verrucosus	GPA2_partial	RZC35091.1	*Henosepilachna vigintioctopunctata*	CAPA receptor (Hvp_A5)	PV645201	*Coccinella septempunctata*	PDF receptor	XP_044752499.1
Bombyx_mori	GPA2_precursor	NP_001124375.1	*Bombyx mori*	CAPA receptor	XP_037871108.2	*Harmonia axyridis*	PDF receptor	XP_045471391.1
Drosophila_melanogaster	GPA2	NP_001104054.3	*Harmonia axyridis*	CAPA receptor	XP_045480343.1	*Tribolium castaneum*	PDF receptor	XP_008193265.1
Henosepilachna vigintioctopunctata	Hvp_GPB5	PV645165	*Coccinella septempunctata*	CAPA receptor	XP_044749380.1	*Drosophila melanogaster*	PDF receptor	NP_570007.2
Coccinella_axyridis	GPB5	XP_045473976.1	*Zophobas morio*	CAPA receptor	XP_063912494.1	*Henosepilachna vigintioctopunctata*	PTH-like receptor (Hvp_B4)	PV645208
Coccinella_septempunctata	GPB5	XP_044754549.1	*Tribolium castaneum*	CAPA receptor	XP_064214691.1	*Harmonia axyridis*	PTH-like receptor	XP_045463972.1
Tribolium_castaneum	GPB5	NP_001280517.1	*Drosophila melanogaster*	CAPA receptor	NP_996140.1	*Coccinella septempunctata*	PTH-like receptor	XP_044760439.1
Asbolus_verrucosus	GPB5	RZB89874.1	*Henosepilachna vigintioctopunctata*	CCH1 receptor (Hvp_A6)	PV645202	*Tribolium madens*	PTH-like receptor	XP_044253435.1
Bombyx_mori	GPB5	NP_001124380.1	*Bombyx mori*	CCH1 receptor	NP_001127712.1	*Drosophila melanogaster*	PTH-like receptor	NP_725175.3
Drosophila_melanogaster	GPB5	NP_001015386.3	*Coccinella septempunctata*	CCH1 receptor	XP_044759703.1	*Henosepilachna vigintioctopunctata*	Hvp_LGR1 receptor	PV645176
Henosepilachna vigintioctopunctata	Hvp_Hansolin	PV645166	*Harmonia axyridis*	CCH1 receptor	XP_045477761.1	*Bombyx mori*	LGR1 receptor	XP_037868182.1
Coccinella_axyridis	Hansolin	XP_045482972.1	*Tribolium madens*	CCH1 receptor	XP_044272536.1	*Harmonia axyridis*	LGR1 receptor	XP_045463486.1
Coccinella_septempunctata	Hansolin	XP_044761764.1	*Drosophila melanogaster*	CCH1 receptor	NP_611241.2	*Coccinella septempunctata*	LGR1 receptor	XP_044744562.1
Henosepilachna vigintioctopunctata	Hvp_IDLSRF_like	PV645167	*Henosepilachna vigintioctopunctata*	CCH2 receptor (Hvp_A7)	PV645203	*Tribolium castaneum*	LGR1 receptor	XP_008195486.1
Coccinella_septempunctata	IDLSRF_Like	XP_044765879.1	*Bombyx mori*	CCH2 receptor	NP_001127711.1	*Drosophila melanogaster*	LGR1 receptor	NP_524393.2

Coccinella_axyridis	IDLSRF_Like	XP_04548 2509.1	*Harmonia axyridis*	CCH2 receptor	XP_04547 6918.1	*Henosepilachna vigintioctopunctata*	Hvp_LGR2 receptor	PV645177
Tribolium_castaneum	IDLSRF_Like	XP_00820 0700.1	*Coccinella septempunctata*	CCH2 receptor	XP_04475 8875.1	*Coccinella septempunctata*	LGR2 receptor	XP_04474 9517.1
Bombyx_mori	IDLSRF_Like	XP_00492 3292.1	*Tribolium madens*	CCH2 receptor	XP_04425 2528.1	*Harmonia axyridis*	LGR2 receptor	XP_04547 9242.1
Henosepilachna vigintioctopunctata	Hvp_ILP1_like	PV645168	*Drosophila melanogaster*	CCH2 receptor	NP_00135 6958.1	*Tribolium castaneum*	LGR2 receptor	XP_00819 2239.1
Tribolium_castaneum	ILP1	XP_06421 5045.1	*Henosepilachna vigintioctopunctata*	CCAP receptor (Hvp_A8)	PV645204	*Drosophila melanogaster*	LGR2 receptor	NP_47670 2.1
Henosepilachna vigintioctopunctata	Hvp_ILP2_like	PV645169				*Neocloeon triangulifer*	NtrGluR	XP_05949 1291

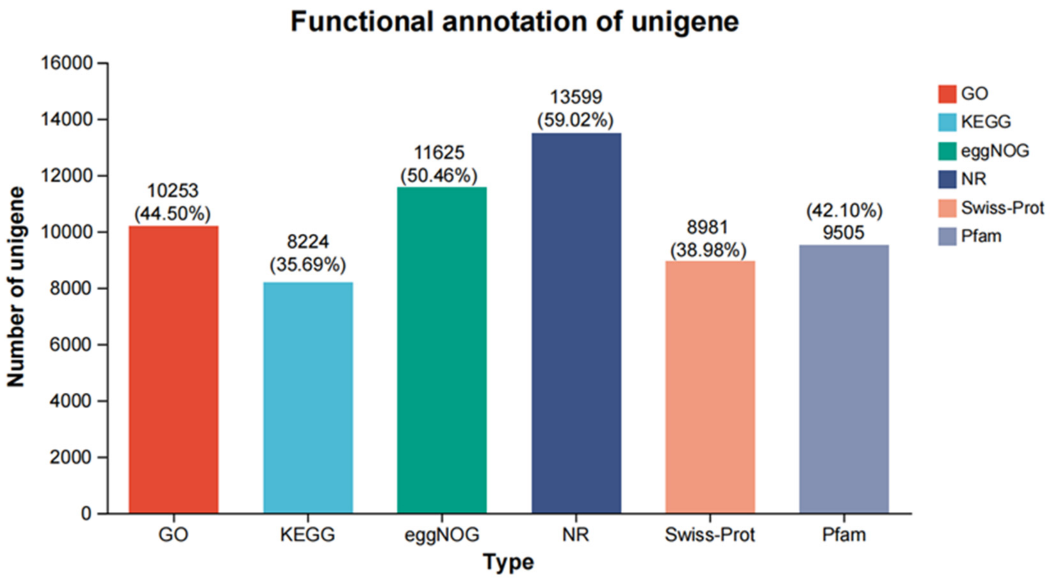

Figure S1. Functional annotation of unigenes from transcriptome of *H. vigintioctopunctata* CNS.

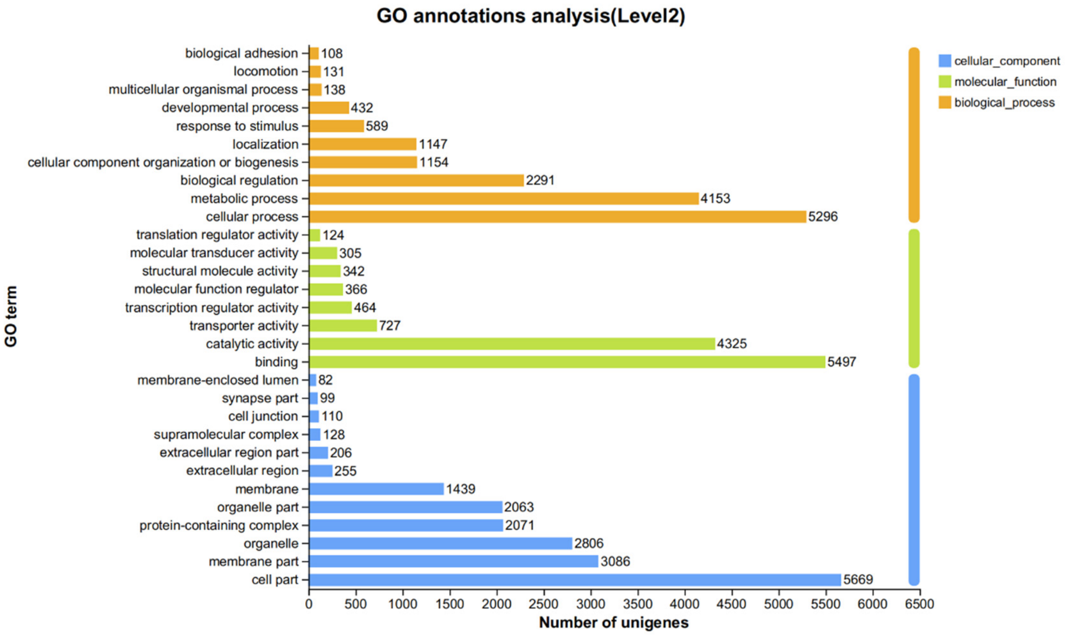

Figure S2. GO annotations analysis of unigenes from transcriptome of *H. vigintioctopunctata* CNS.

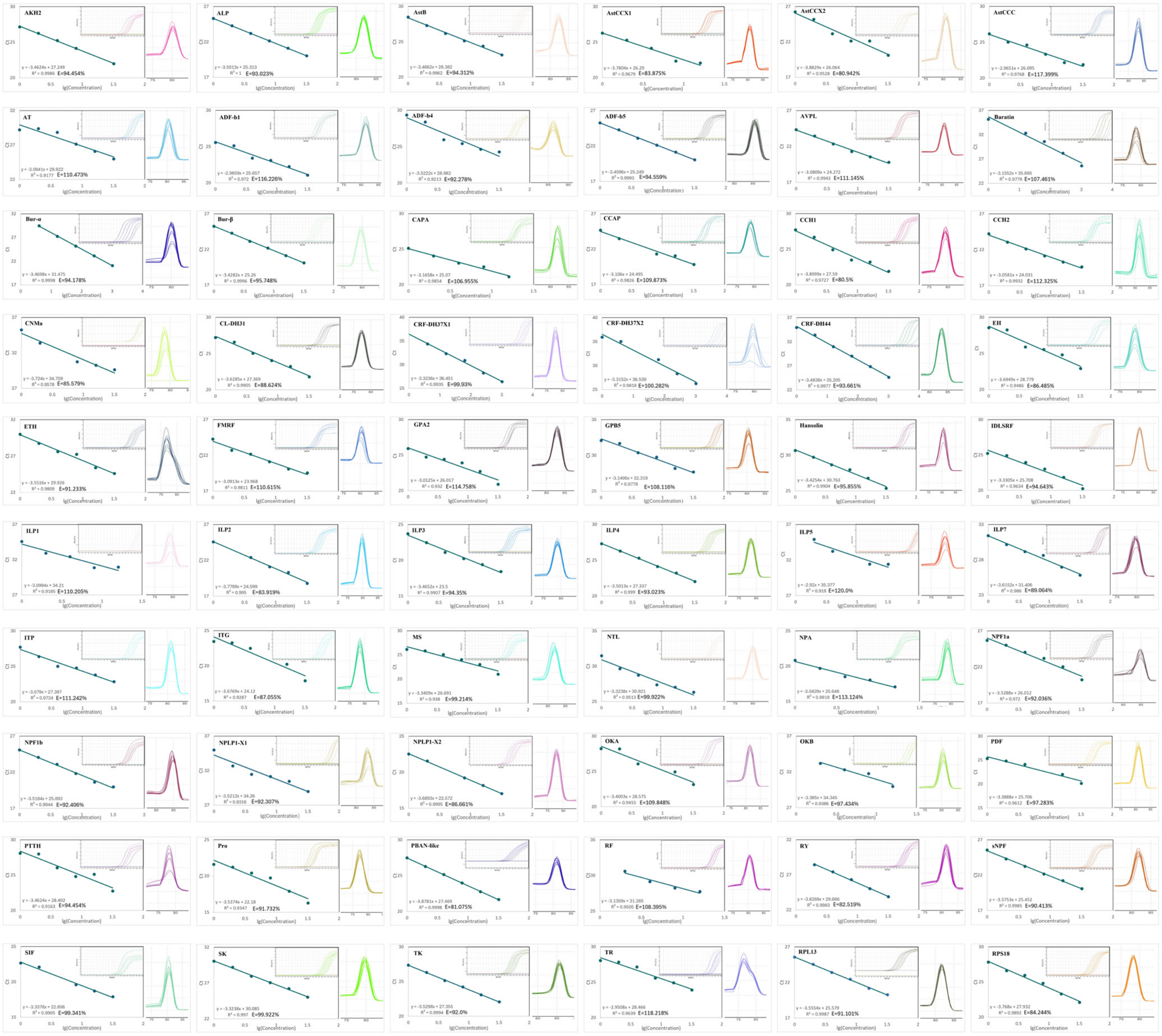

Figure S3. The standard curves, melting curves, amplification plots of the primers for quantitative Real-Time PCR (qRT-PCR) of 58 neuropeptide genes.

Full names of gene abbreviations in the figure: Adipokinetic hormone 2 (*AKH2*), Agatoxin-like (*ALP*), Allatostatin B (*AstB*), Allatostatin CC X1 (*AstCC X1*), Allatostatin CC X2 (*AstCC X2*), Allatostatin CCC (*AstCCC*), Allatotropin (*AT*), Antidiuretic factor b-1 (*ADF-b1*), Antidiuretic factor b-4 (*ADF-b4*), Antidiuretic factor b-5 (*ADF-b5*), Arginine-vasopressin-like (*AVPL*), *Baratin*, Bursicon alpha (*Bur α*), Bursicon beta (*Bur β*), Capability (*CAPA*), Crustacean cardioactive peptide (*CCAP*), CCHamide 1 (*CCH1*), CCHamide 2 (*CCH2*), CNMamide (*CNMa*), Calcitonin-like diuretic hormone 31 (*CL-DH31*), Corticotropin-releasing factor-like-diuretic hormone 37 X1 (*CRF-DH 37 X1*), Corticotropin-releasing factor-like-diuretic hormone 37 X2 (*CRF-DH 37 X2*), Corticotropin-releasing factor-like-diuretic hormone 44 (*CRF-DH 44*), Eclosion hormone (*EH*), Ecdysis triggering hormone (*ETH*), FMRFamide (*FMRF*), Glycoprotein hormone alpha 2 (*GPA2*), Glycoprotein hormone beta 5 (*GPB5*), *Hansolin*, IDLSRF-like peptide (*IDLSRF*), Insulin-like peptide 1 (*ILP1*), Insulin-like peptide 2 (*ILP2*), Insulin-like peptide 3 (*ILP3*), Insulin-like peptide 4

(*ILP4*), Insulin-like peptide 5 (*ILP5*), Insulin-like peptide 7 (*ILP7*), ion transport peptide (*ITP*), ITG-like (*ITG*), Myosuppressin (*MS*), Natalisin (*NTL*), Neuroparsin A (*NPA*), Neuropeptide F 1a (*NPF1a*), Neuropeptide F 1b (*NPF1b*), Neuropeptide-like precursor 1 X1 (*NPLP1 X1*), Neuropeptide-like precursor 1 X2 (*NPLP1 X2*), Orcokinin A (*OK A*), Orcokinin B (*OK B*), Pigment-dispersing factor (*PDF*), Prothoracicotropic hormone (*PTTH*), Proctolin (*Pro*), phermone biosynthesis activating neuropeptide like (*PBAN-like*), RFLamide (*RF*), Ryamide (*RY*), short neuropeptide F (*sNPF*), SIFamide (*SIF*), Sulfakinin (*SK*), Tachykinin (*TK*), Trissin (*TR*).

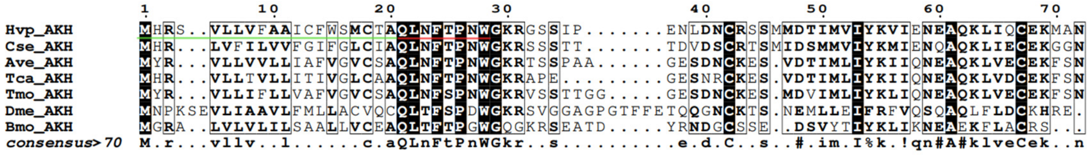

Figure S4. Sequence alignment of Adipokinetic hormone (AKH) precursors.

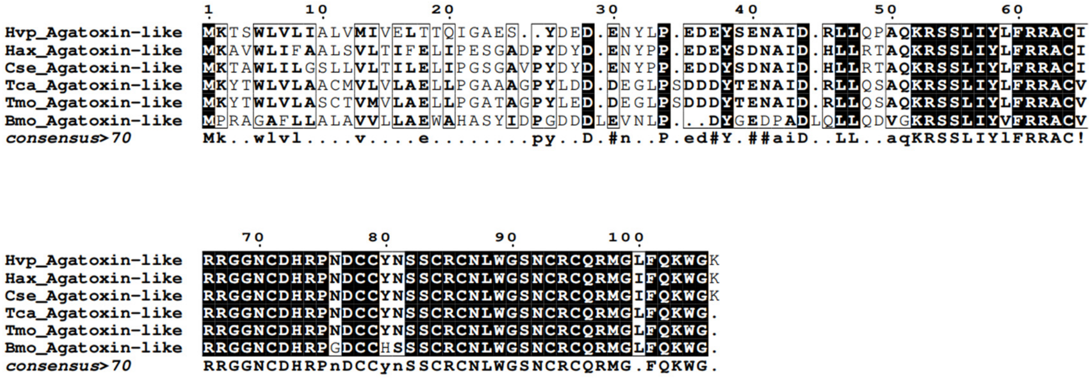

Figure S5. Sequence alignment of Agatoxin-like (ALP) precursors.

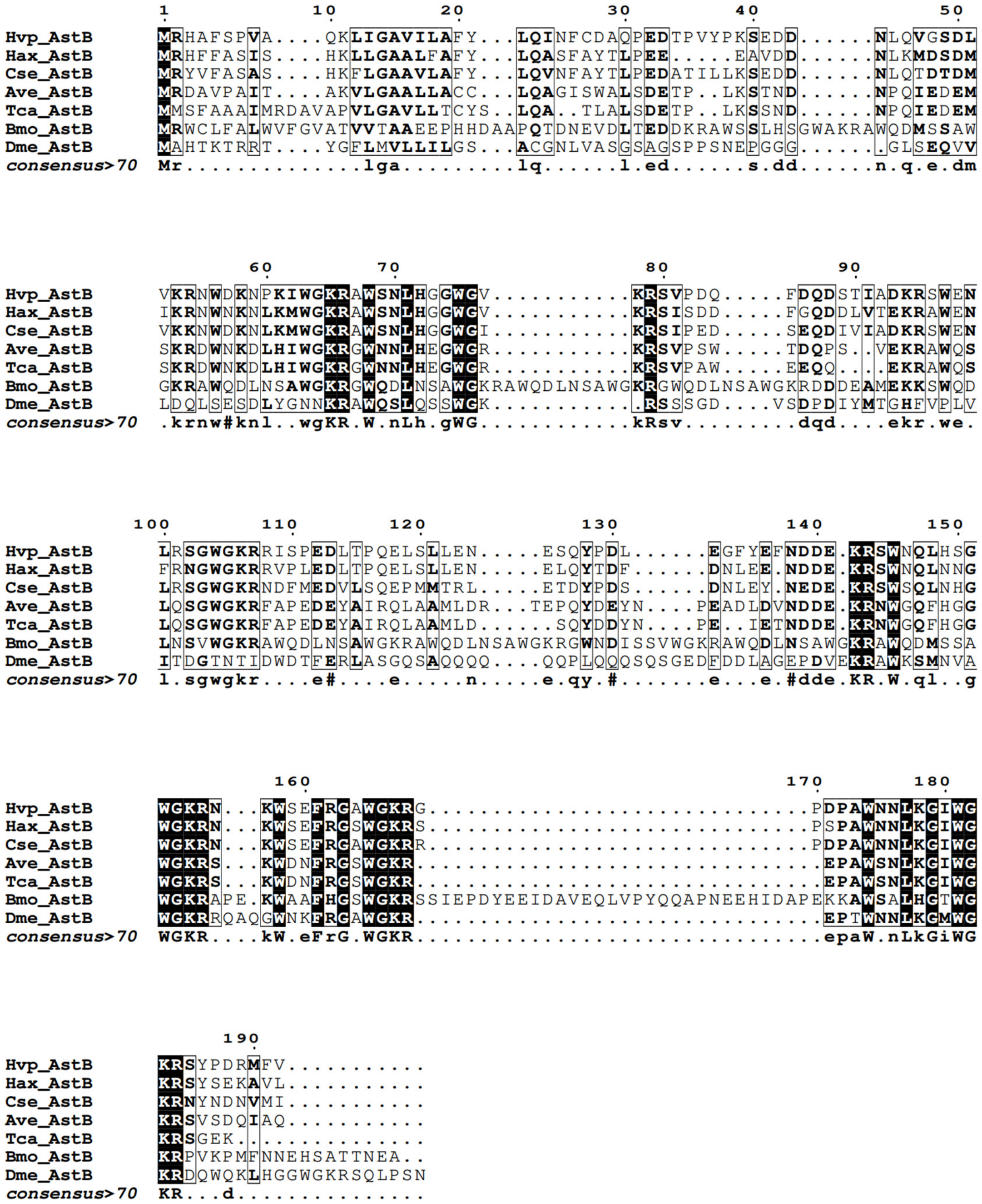

Figure S6. Sequence alignment of Allatostatin B (AstB) precursors.

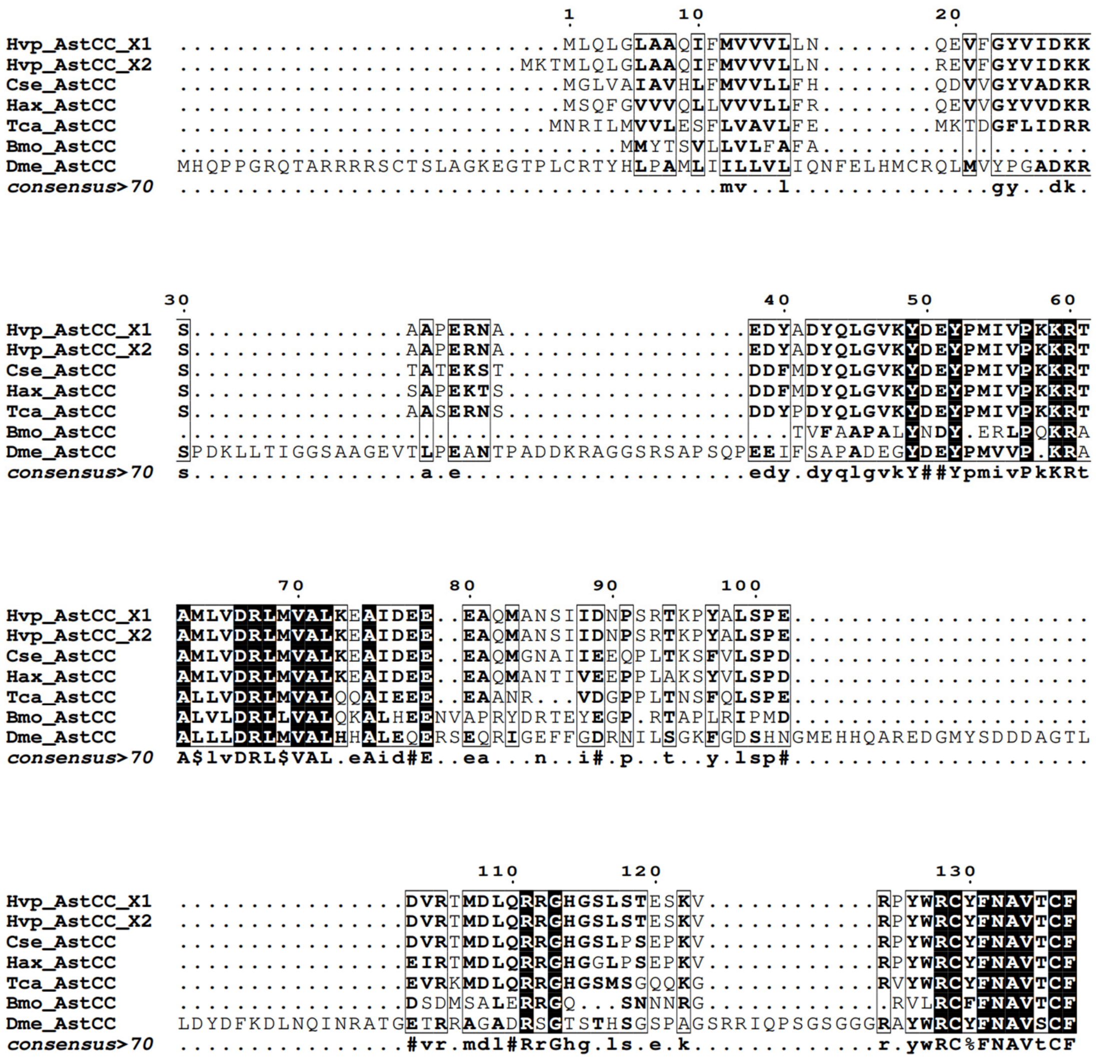

Figure S7. Sequence alignment of Allatostatin CC (AstCC) precursors.

```
                 1        10         20         30         40
                 .        .          .          .          .
Hvp_AstCCC       MSSELVKMQR...FLVLCLLIAVTAAKPS...........HFTDN.QVSPD.AEKGDFMDPG.L
Hax_AstCCC       MSSDLVRLTS...FLLLCLLAASIAAKPS...........HFPDN.QVTPD.GDRSDMIDPG.L
Cse_AstCCC       MNTDLVRIKS...FLLLLIVVGVVAAKPS...........HFSDN.QVTPDSGDRSDVMDPG.L
Tca_AstCCC       MAAQLPRYLTKTLFIFLIATLVVANARPN...........HFGDASQVVGEPADGNNLLDS...
Bmo_AstCCC       .MKFIRNNLGLAVLFGVFALLVVTNSAPMDPD........EDQSESSVVGHTDNEVDLSGSWDA
Dme_AstCCC       MMKFVQILLCYG..LLLTLFFALSEARPSGAETGPDSDGLDGQDAEDVRGAYGGGYDMPAQA.I
consensus>70     m...............l........a.P...............d..qV.....d..#.......
```

```
                    50        60         70         80         90
                    .         .          .          .          .
Hvp_AstCCC       LR..ALERYD.ALRRLASDYENANILNQNP..LEPLFRNTE.........MKRQGRSYRPCYFN
Hax_AstCCC       LN..AWRRYDLMMRRLTADYDNANMANENP..LERMFRNPE.........MKRQGRSYRQCYFN
Cse_AstCCC       IN..AWRRYDFLMRRLAADYDNGNMANDNP..LDRLFRNPE.........MKRQGRSYRQCYFN
Tca_AstCCC       ....RLKPWELEMLVQRLSEISSQTGGDFA..WDKSIRLPE.........AKRQSR.YRQCYFN
Bmo_AstCCC       VDAAALRKLLTDLGVEDRMNRVARSWPQATEPRGWAMRNVDGRLVRPWRADKRQVR.FRQCYFN
Dme_AstCCC       YPNIPMDRLQMLFAQYRPTSYSAYLRSPTYGNVNELYRLPE.........SKRQVR.YRQCYFN
consensus>70     .........d..m..............q.....e...R..#..........KRQ.R.%RqCYFN
```

```
                 100
                  .
Hvp_AstCCC       PISCFKK
Hax_AstCCC       PISCFKK
Cse_AstCCC       PISCFKK
Tca_AstCCC       PISCFRK
Bmo_AstCCC       PISCFRK
Dme_AstCCC       PISCFRK
consensus>70     PISCF.K
```

Figure S8. Sequence alignment of Allatostatin CCC (AstCCC) precursors.

```
                                                                        1
                                                                        .
Hvp_AT           .......................................................MELHHVIVL
Cse_AT           .......................................................MALHHVLLI
Hax_AT           MKEKKKLLSLQVWEVIVTPPDYMKDSLGACSSNCLPNTKVGHCCSSAVSCSINKSMALHHVLLV
Tca_AT           .......................................................MAFHHAALF
Tmo_AT           .......................................................MAFQHAALF
consensus>70     .......................................................Ma.hH..l.
```

```
                 10         20         30         40         50         60         70
                 .          .          .          .          .          .          .
Hvp_AT           LVIILLWANLASVQGRKRDDMPRAFRRSADASE.RRTRKFDEMVLATARGYGKRANERFKDN.S
Cse_AT           FIIIVLWTNIASVQARRGEEMAREFRRGDDMKE.RRTRKFDEMILSTARGYGKRTNGQLRKD.F
Hax_AT           FIIIV.WTNVASVQARRDEEMAREFRRGEDMKE.RRTRKFDEMVLSTARGYGKRTNGQLRKD.F
Tca_AT           FTLMFLWLLLANAQGRRDAKYPQVRTPQQRLTRGIEALKYHNMDLGTARGYGKRAVDMHNVN.F
Tmo_AT           LTLIFLWLMLSNVQGRR.EKVTQVRTPQQRLTRGIEHFKYHNMDLGTARGYGKRAVDMHNVNNF
consensus>70     ...i.lW...a.vQ.Rr.e.........q.m.......K%.#M.L.TARGYGKR.......#.f
```

```
                          80         90
                          .          .
Hvp_AT           LLEWIGLE.RMRGLGYPTRVMSNSEMISE
Cse_AT           LLEWIAME.RMRDLGYPRYVMNNAELSPE
Hax_AT           LLEWIAME.RMRDLGYPRFVMNNADLTPE
Tca_AT           LLEWIALETRMRNLGIPRNLLRDQETIPE
Tmo_AT           LLEWIALETRMRDLGIPRNLLRDQETIPE
consensus>70     LLEWIa$E.RMRdLG.Pr..$.#.#..pE
```

Figure S9. Sequence alignment of Allatotropin (AT) precursors.

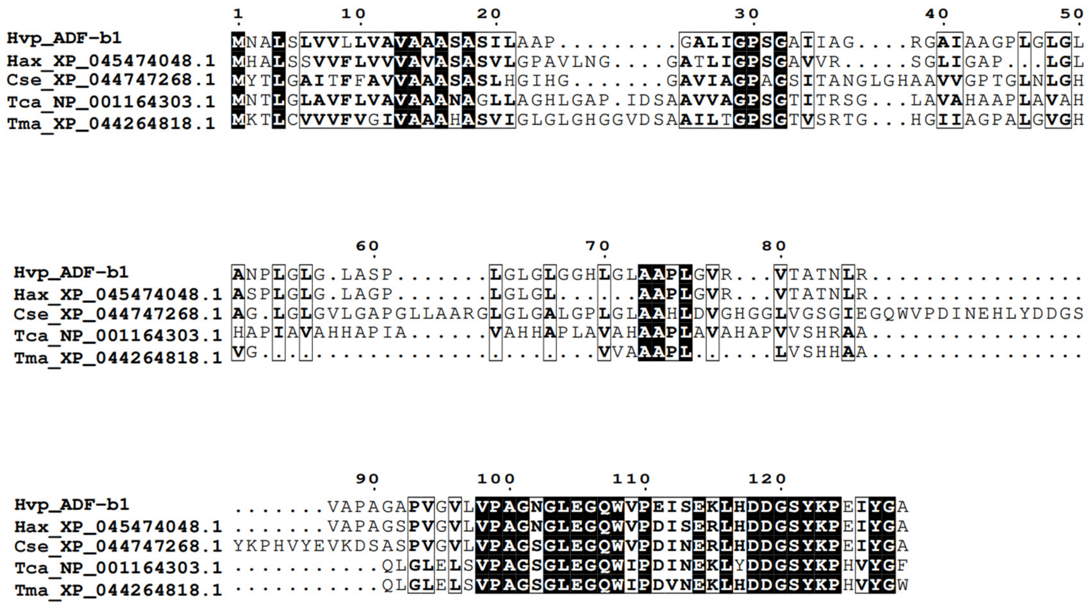

Figure S10. Sequence alignment of Antidiuretic factor b-1 (ADF-b1) precursors.

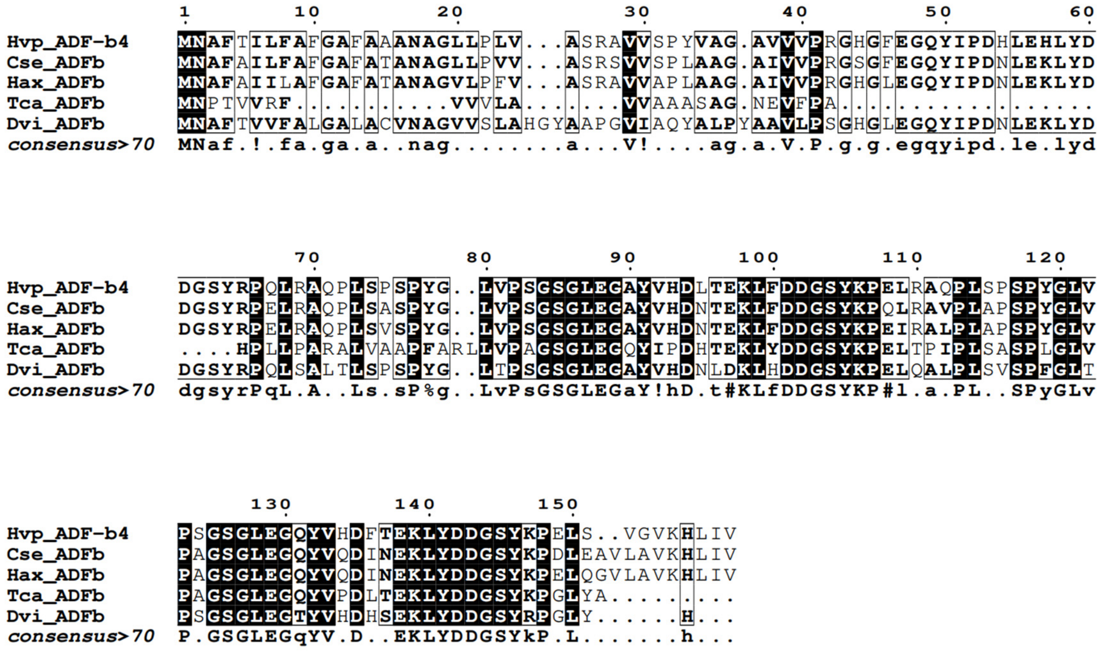

Figure S11. Sequence alignment of Antidiuretic factor b-4 (ADF-b4) precursors.

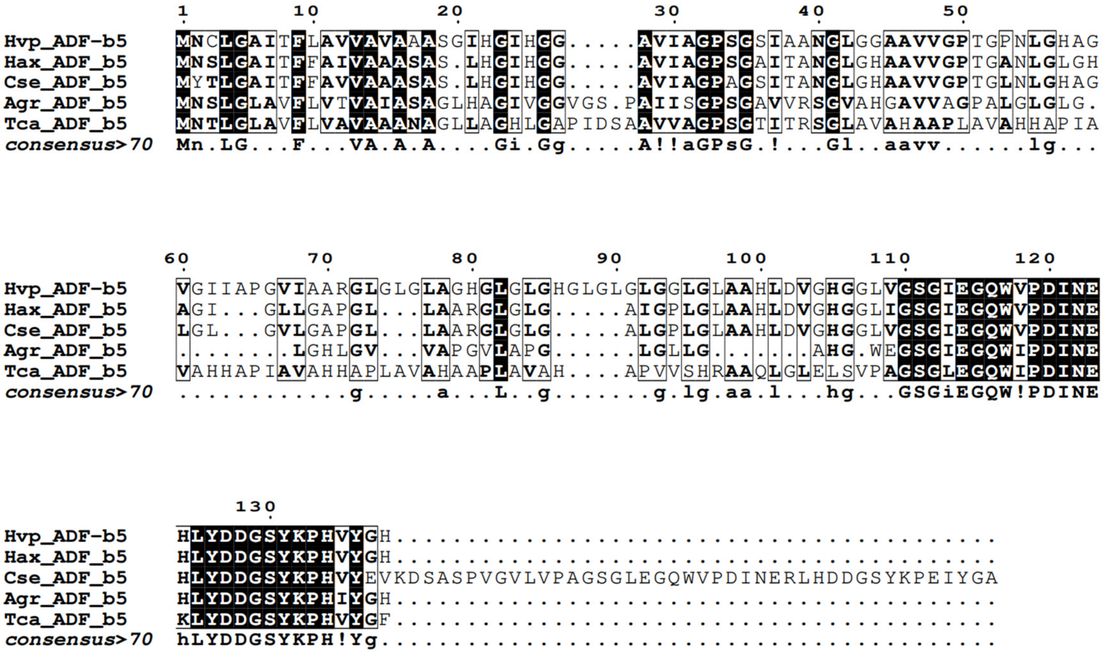

Figure S12. Sequence alignment of Antidiuretic factor b-5 (ADF-b5) precursors.

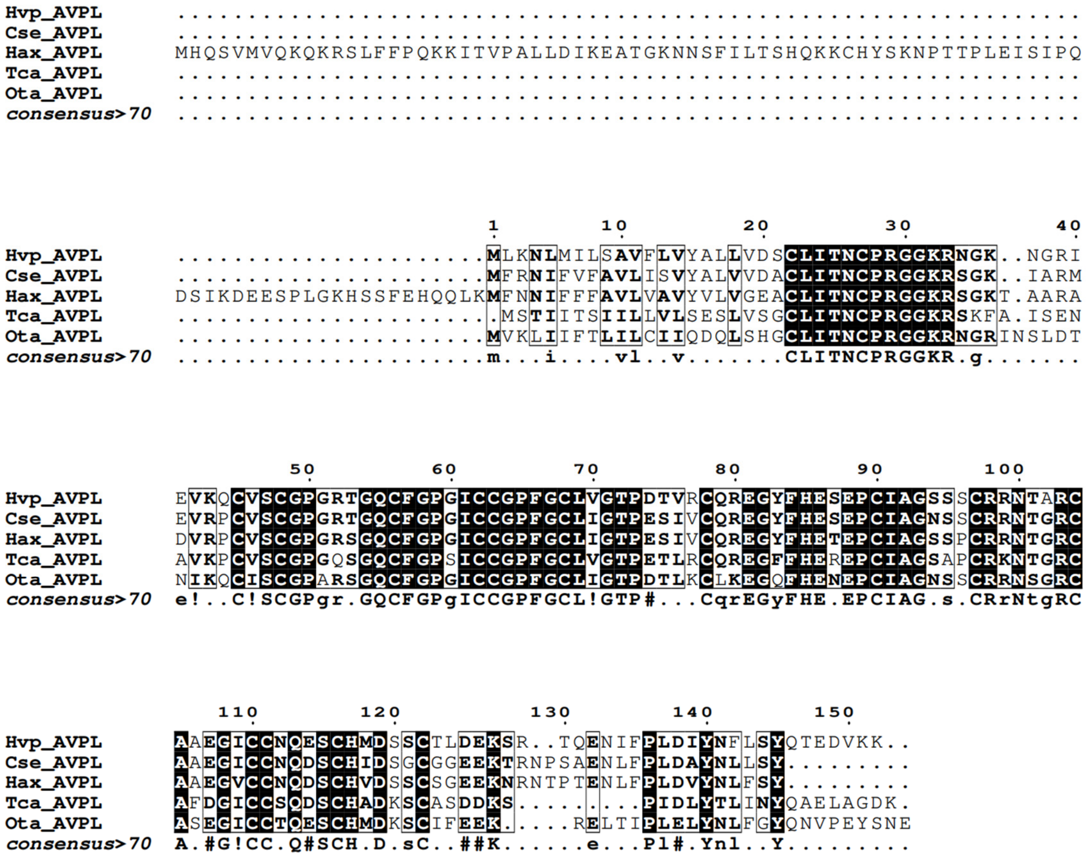

Figure S13. Sequence alignment of Arginine-vasopressin-like (AVPL) precursors.

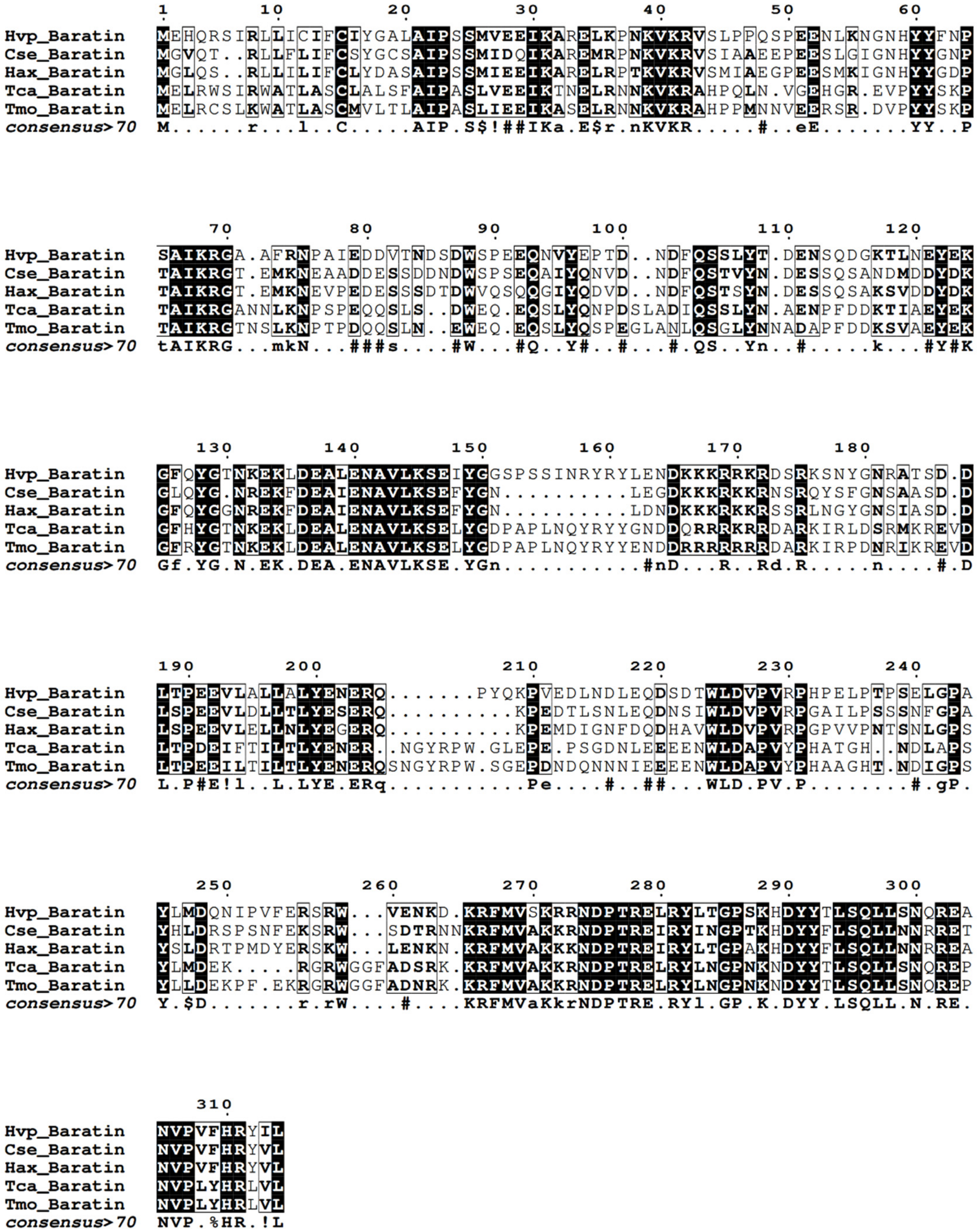

Figure S14. Sequence alignment of Baratin (NVP-like) precursors.

```
                        1        10        20        30        40        50
Hvp_bursicon_alpha      MFNRIFRITS..VSWNILSRTLLCIVLGSMCLDPRYT..KYEVSSASTTDECQVTPVIH
Cse_bursicon_alpha      MI..IAAIIH..LSCNILTKSIMFMIIGSMCIDPRYI..QYEVSAASKTDLCEVTPVIH
Tca_bursicon_alpha      .............................MCLDPRLN.SKIQVSGASTTDECQVTPVIH
Atu_bursicon_alpha      MLRQILDI....VPQRLWQITTIFIILGNMCMDPRLSRGTLEVSGTSTTDECQVTPVIH
Bom_bursiocn_alpha      .....MSVLN....TFLVIVALILCYVNDFPVTGHEV..QLPPGTKFFCQECQMTAVIH
Dme_bursicon_alpha      MLRHLLRHENNKVFVLILLYCVLVSILKLCTAQPDSSVAATDNDITHLGDDCQVTPVIH
consensus>70            ................................dp.......e.......#eC#vTpVIH

                            60        70        80        90       100       110
Hvp_bursicon_alpha      VLQYPGCVPKPIPSFACIGRCASYIQVSGSKIWQMERSCMCCQESGEREASVSLFCPKA
Cse_bursicon_alpha      VLQYPGCVPKPIPSFACIGRCGSYLQVSGSKIWQMERSCMCCQESGEREASVSLFCPKA
Tca_bursicon_alpha      VLQYPGCVPKPIPSFACIGRCASYIQVSGSKIWQMERSCMCCQESGEREASVSLFCPKA
Atu_bursicon_alpha      VLQYPGCVPKPIPSFACIGRCASYIQVSGSKIWQMERSCMCCQESGEREASVSLFCPKA
Bom_bursiocn_alpha      VLKHRGCKPKAIPSFACIGKCTSYVQVSGSKIWQMERTCNCCQESGEREATVVLFCPDA
Dme_bursicon_alpha      VLQYPGCVPKPIPSFACVGRCASYIQVSGSKIWQMERSCMCCQESGEREAAVSLFCPKV
consensus>70            VLqypGCvPKpIPSFAC!GrC.SYiQVSGSKIWQMERsCmCCQESGEREA.VsLFCPka

                            120       130       140       150       160
Hvp_bursicon_alpha      KPGERKFIKVTTKAPLECMCRPCTGVEESAIIPQEIAGYADEGPLSNHFLKSQPQ
Cse_bursicon_alpha      KPGERKFLKVTTKAPLNCMCRPCTSIEESAVIPQEIAGYADEGPLSDHFLKSQPQ
Tca_bursicon_alpha      KPGERKFIKVTTKAPLECMCRPCTGVEESAVIPQEIAGYADEGPLSNHFLKSHSQ
Atu_bursicon_alpha      KPGERKFIKVTTKAPLECMCRPCTGVEESAIIPQEIAGYADEGPLSNHFLKSHQQ
Bom_bursiocn_alpha      QNEEKRFRKVSTKAPLQCMCRPCGSIEESSIIPQEVAGYSEEGPLYNHFRKSL..
Dme_bursicon_alpha      KPGERKFKKVLTKAPLECMCRPCTSIEESGIIPQEIAGYSDEGPLNNHFRRIALQ
consensus>70            kpgErkF.KV.TKAPL#CMCRPCt.!EES.!IPQE!AGY.#EGPL.#HF.ks..q
```

Figure S15. Sequence alignment of bursicon alpha (bur α) precursors.

```
                        1        10        20        30        40        50
Hvp_bursicon_beta       MAIVGRRLGQNILVLCIVISLGFCVSEIAD....ETCETLPSEIHLVKEEYDELGRLQRT
Dpo_bursicon_beta       MFTKGL.....LLIFAIFASYGYCVSELPD....ESCETLPSDIHITKEEFDELGRLQRT
Lde_bursicon_beta       MLFYT......VFFGAMAVSFVYCLNDMSE....ETCETLPSEIHLTKEEYDELGRLQRT
Sor_bursicon_beta       .MSYFR.....FFILTLLISYVRGVTELAE....ETCETLPSEIHITKEEFDELGRLQRT
Bmo_bursicon_beta       MNIMIT......KIFFLVQLFYIVVSKSSA...EENCETVASEVHVTKEEYDEMGRLLRS
Dme_bursicon_beta       MHVQEL.....LFVAAILVPQCLRALRYSQGTGDENCETLKSEIHLIKEEFDELGRMQRT
consensus>70            m............i...............d....E.CETl.S#!H..KEE%DE$GR$qRt

                          60        70        80        90       100       110
Hvp_bursicon_beta       CNGEIAVNKCEGSCKSQVQPSVITPTGFLKECYCCRESFLRERIITLNHCYDPDGMRLTN
Dpo_bursicon_beta       CNGEVAVNKCEGSCKSQVQPSVITPTGFLKECYCCRESYLRERIVTLTHCYDPDGLRLTG
Lde_bursicon_beta       CNGEVAVNKCEGSCKSQVQPSVITPTGFLKECYCCRESFLRERIITLSHCYDPDGVRLTG
Sor_bursicon_beta       CNGEVAVNKCEGSCKSQVQPSVITPTGFLKECYCCRESYLRERVITLTHCYDPDGVRLTN
Bmo_bursicon_beta       CSGEVSVNKCEGMCNSQVHPSISSPTGFQKECFCCREKFLRERLVTLTHCYDPDGIRFED
Dme_bursicon_beta       CNADVIVNKCEGLCNSQVQPSVITPTGFLKECYCCRESFLKEKVITLTHCYDPDGTRLTS
consensus>70            Cng#!.VNKCEG.C.SQVqPS!itPTGFlKEC%CCREs%LrEri!TL.HCYDPDG.Rlt.

                          120       130       140
Hvp_bursicon_beta       EGNNAMDVKLREPAECKCYKCGDFSR
Dpo_bursicon_beta       EGENSMDIKLREPSECKCYKCGDFSR
Lde_bursicon_beta       EGDNAMDIKLREPSECKCFKCGDFSR
Sor_bursicon_beta       EGYNAMDIKLREPSECKCYKCGDFSR
Bmo_bursicon_beta       EENALMEVRLREPDECECYKCGDFSR
Dme_bursicon_beta       PEMGSMDIRLREPTECKCFKCGDFTR
consensus>70            e....M#!.LREP.ECkC%KCGDFsR
```

Figure S16. Sequence alignment of bursicon beta (bur β) precursors.

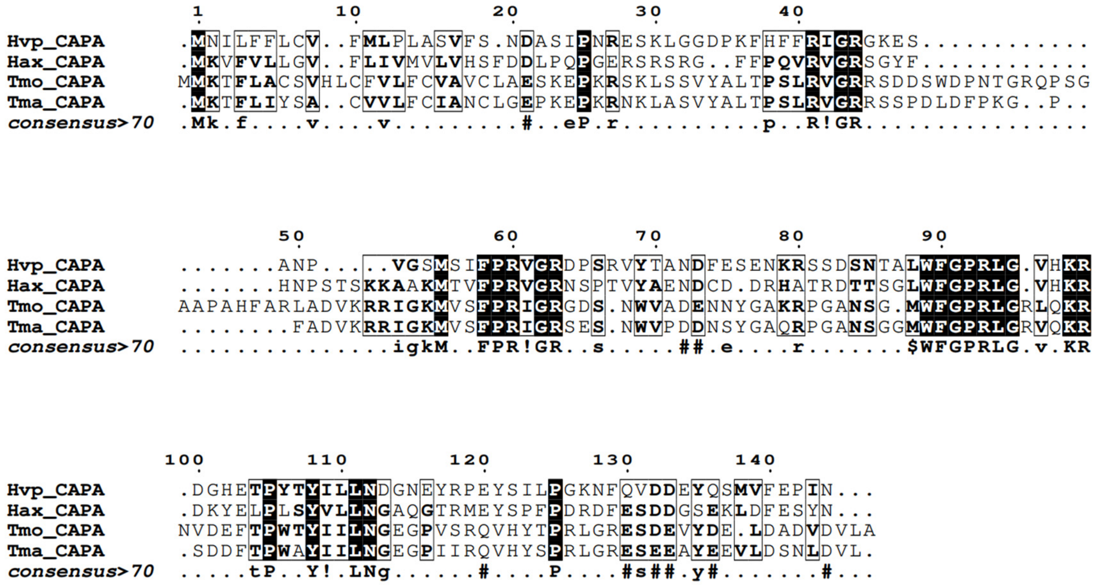

Figure S17. Sequence alignment of Capability (CAPA) precursors.

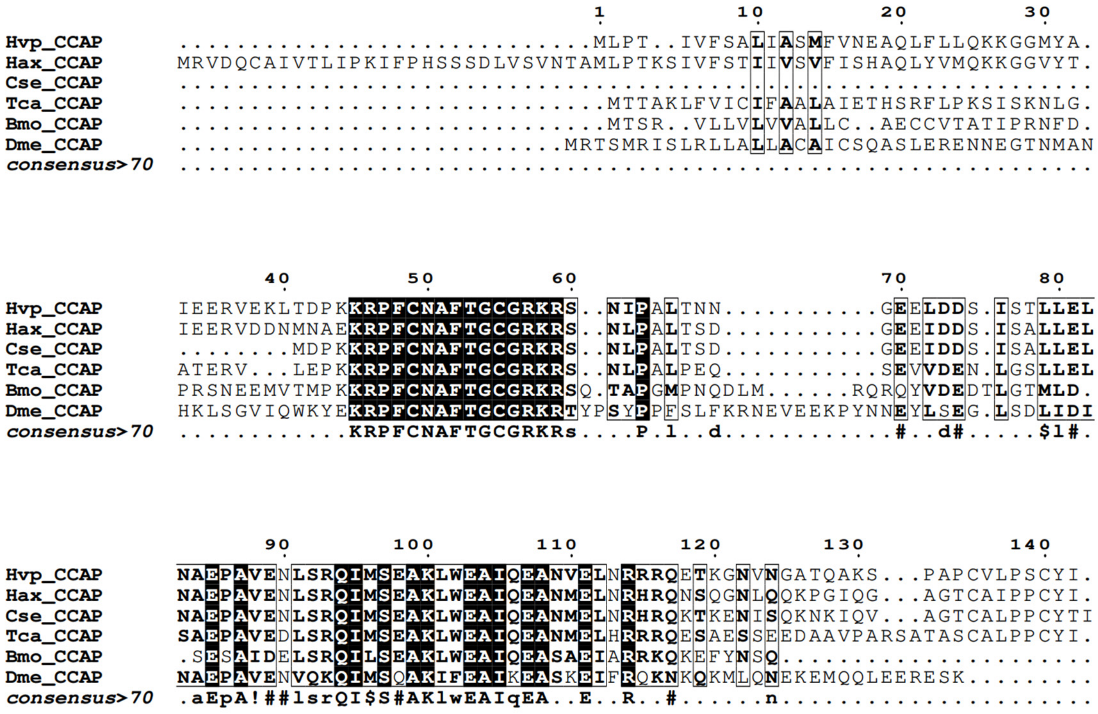

Figure S18. Sequence alignment of Crustacean cardioactive peptide (CCAP) precursors.

```
             1         10        20        30        40        50
Hvp_CCH1     .MKKNCVAMDDFSHTAVFRLTVLLTFFLFAECAAGSCLSYGHSCWGAHGKRSGY.......IGN
Cse_CCH1     ...........MSVACTVKLMVVLTFFLFAECAAGSCLSYGHSCWGAHGKRSE........ASS
Hax_CCH1     ...........MTVVCTVKLTVLLTFFLFAECAAGSCLSYGHSCWGAHGKRNGG.......AAM
Tmo_CCH1     .MCHKQTTMMSPLPVKLAKITVVVIFFCFAECAAGSCLSYGHACWGAHGKRNGAHNNNMPGRDA
Bmo_CCH1     MSFAMEKTYKRSGHRATIRILVVLLVICLAECATGSCLSYGHSCWGAHGKRSNK.........
Dme_CCH1     ..........MWYSKCSWTLVVLVALFALVT...GSCLEYGHSCWGAHGKRSGGKAVIDAKQHP
consensus>70 ....................V....f..aeca.GSCLsYGHsCWGAHGKR.............

             60        70        80        90        100       110
Hvp_CCH1     PKYLDSSRLLM.RLMQQNANHRSEMDKEVMRDYENVPSRAEVQQ........LINDVEMESNPM
Cse_CCH1     VPHLDGNRLLLARLLQPNE......NDKELKELDNAPTLLEIKQ........LLNEGGVESNPL
Hax_CCH1     NPQYQSNRLLLSRLMQPNS......MND..KELDNTPTMLEIKQ........LLNEGEVESNPL
Tmo_CCH1     PPVSRDSTWFLSKLVQSPL........DLRYVNDKDLDLPTSQQ........LFADAQIEADPL
Bmo_CCH1     .SPISAPDWYFNRMLRRFAAN....AEMNFQNQDQALNSNDDTN........AIYQNNVEDNAP
Dme_CCH1     LPNSYGLDSVVEQLYNNNNNNQNNQDDDNNDDDSNRNTNANSANNIPLAAPAIISRRESEDRRI
consensus>70 ............$.q..............d.dn.....e..#..........d.e.E.n..

             120       130       140       150       160
Hvp_CCH1     KTL......SDYEADSDVLFDDNVP.VFEGITENHLKPSNTRRISQRILKKRSTKMN.......
Cse_CCH1     KGL......NDYDSAVDVLFDESLP.SYEDISTRRFKSTSGRRIPSRILRKRSTKTT.......
Hax_CCH1     KGM......NDYDSAVDVLFDESLP.SYEDVPVRQFKTTNGRRIPSRIF.KRSTKTT.......
Tmo_CCH1     KGQ......EDYRGLPDAYSNEENV.LFDIYPNQRPRPN..KIKASKYLEKRSTRMI.......
Bmo_CCH1     GSD......RLFTVSDEGPVELPLSRSLEAKPMLDDSLLPKYKIWQLMTGTRDNK.........
Dme_CCH1     GGLKWAQLMRQHRYQLRQLQDQQQQGRGRGGQGQYDAAAESWRKLQQALQAQIDADNENYSGYE
consensus>70 ..........dy....d...#e......e.....................r............

Hvp_CCH1     ...
Cse_CCH1     ...
Hax_CCH1     ...
Tmo_CCH1     ...
Bmo_CCH1     ...
Dme_CCH1     LTK
consensus>70 ...
```

Figure S19. Sequence alignment of CCHamide 1 (CCH1) precursors.

```
                1          10             20            30            40            50            60
Hvp_CCH2        MGFWSTAALSLAFLVLLVVLNFGGAQGKRGCANFGHSCYGGMGKRALDIA...SSNDEFVADMP
Tmo_CCH2        MNCWSSVVVLLAVMAFVLAFHAEAAEAKRGCATFGHSCYGGMGKRASELM...ENNEEILQDVQ
Tca_CCH2        MNCWSTQVVLL...AFVMAFVLAAAEAKRGCATFGHSCYGGMGKRTEN......NNEELLQDVQ
Hax_CCH2        MG..CCWYNLLAFLFIFLVLNFGVSEGKRGCANFGHSCYGGMGKRGHK.........ELLPILD
Cse_CCH2        MG..YSRV.FPAVLFILLVLNVADSEGKRGCANFGHSCYGGMGKRAPK.........ELMQFTD
Bmo_CCH2        .....MAQICLAVSIAVLLMMSQGVSAKRGCSAFGHSCFGGHGKRSGEPAPMDMANQDMMVRHQ
Dme_CCH2        ...MKSTISLLLVVICTVVLAAQQSQAKKGCQAYGHVCYGGHGKRSLSPGSGSGTGMGGGMGEA
consensus>70    m.........la.....l.l.....e.KrGCa.%GHsC%GGmGKR...........e.l...q
```

```
                       70          80          90                    100
Hvp_CCH2        YAQDAGLVFTG.PRSGGYNSYPRERMSPQQ....YEQ..........LSRILKQWIQFHKSPQ
Tmo_CCH2        GEENPAFVFTG.PRSE.YKPERPPKLSPQQ....YDT..........ISRVIRQWIQSYRGAQ
Tca_CCH2        SEENPAFVFTG.PRSE.NQQ....KLTPEQ....YDN..........ISRVIRQWILSYRRAQ
Hax_CCH2        PTQEQGEILIG.SRFISDKPYVKLKLNPLQ....YQQ..........LSRFFKQWISSRSS.Q
Cse_CCH2        PRQNQGEILIG.SRFFNDKPYVKLKMNRLQ....YQQ..........LSRILRQWIHSQTMPQ
Bmo_CCH2        LGQEETPPHPGYPHSSYNVLQPGDDIIPIRDGGVYDHDAAARDVMKYKLRNIFKHWMDNYRRSQ
Dme_CCH2        ASGGQEPDYVR.PNGLLPMMAPNEQVPLEGDFNDYPAR........QVLYKIMKSWFNRPRRPA
consensus>70    ..qn...v..g.pr...........l.p.q....Yd...........lsri..qWi......q
```

```
               110
Hvp_CCH2        SYQRDENMV...........
Tmo_CCH2        EMREN...............
Tca_CCH2        EMRPDYN.............
Hax_CCH2        HYEDGQNEI...........
Cse_CCH2        NFEDDKNQI...........
Bmo_CCH2        NTNDEYFLETI.........
Dme_CCH2        SRLGELDYPLANSAELNGVN
consensus>70    ....d.n.............
```

Figure S20. Sequence alignment of CCHamide 2 (CCH2) precursors.

```
                                        1                 10        20
Hvp_CNMa        .......................MIILLG............IILVSRVNCKSFLAN...LSEN.
Hax_CNMa        .......................MIVLLG............IILVLRVDCRSFIQG...YPRV.
Tmo_CNMa        .......................MRIAFG............VIFVTGIFGGFFGDN...ASASP
Tca_CNMa        .......................MRIAFG............VIFVTGIFGGFFAEN...VFALP
Dme_CNMa        MSALSAPTTCGCSPVHWAIVIVLLSVAIGPGDAMARPARNTQLLFSELLGGGNDDNNYYGDQLK
consensus>70    .......................$.!..G............ii.v..v....f.dn.......
```

```
                     30         40         50                60          70
Hvp_CNMa        .SDYRSLNPEMMEN.INEEFDTYLDLG........GANGDSDEYD...ILSEQIFSNKLNTGRQ
Hax_CNMa        .HPGYIIRSDSIGK.IKEEYDAYIDLK........DNEDDSNEYD...ILSEE..ERFISNGHQ
Tmo_CNMa        VHHHHVISKDLDDTTADKMNKPYISIS........HDRADNSDKADVITRVQKVFVDSPKSNVS
Tca_CNMa        VARHHVITKDLD...LDEMYKSYTSIS........NDRDDYNDKV.....TSRVYLDSPQISVS
Dme_CNMa        YQQQQQQQEQKQQRVPAFARKWPSLRDLLLTVDYDDFGVTQESEEQVAPSSRLLARLHRLGDN
consensus>70    .........#............y.............d..d.n#....................
```

```
                   80            90            100       110        120
Hvp_CNMa        FGLNKDRE....LIQTYILVKTMR......QRNKRYVNYLTLCHFKICTIGR.RNARHENVLKR
Hax_CNMa        FGTDRDRE....LIQTYILVKTLR......QRNKRYSSYLTLCHFKICNMGKKRRTRFVHGMES
Tmo_CNMa        KNGKQQKT....KQTAYLLVKTMR......QRNKRYISYLTLCHFKICNMGRKRTTRYFHMIRR
Tca_CNMa        KNGKQQKT....KQTAYLLVNTMS......QRNKRYISYLTLCHFKICNMGRKRTSRYFHMIRQ
Dme_CNMa        GGGEELRYNVVNELTNMPSKKVMPGHPLKDHNTKKNVQYMSPCHFKICNMGRKRNAGFNSY...
consensus>70    .....d..........y.lvkt$.......qrnKryv.Y$tlCHFKICnmGrkR..rf......
```

```
               130        140
Hvp_CNMa        ERKAPFKFRSKKN
Hax_CNMa        SRENRI.......
Tmo_CNMa        LD.NES.......
Tca_CNMa        LDDNEA.......
Dme_CNMa        .............
consensus>70    .............
```

Figure S21. Sequence alignment of CNMamide (CNMa) precursors.

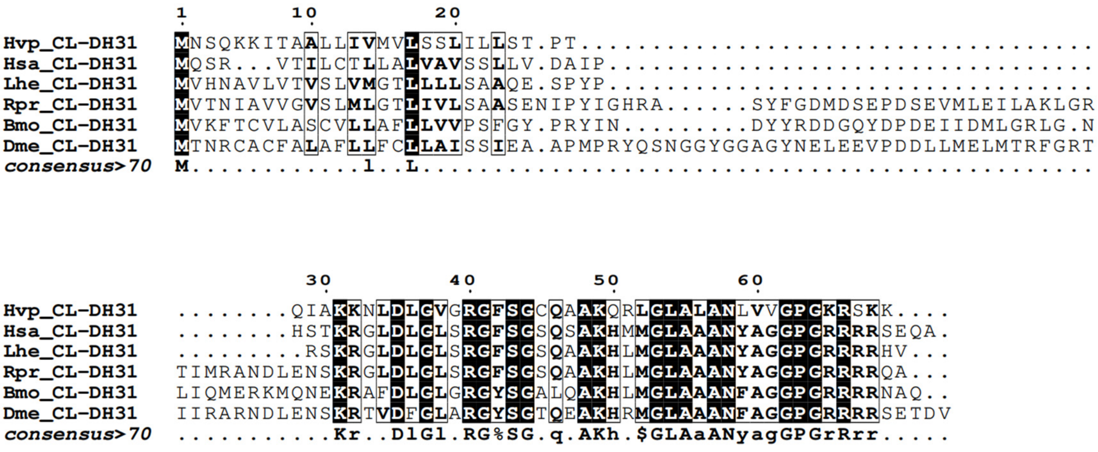

Figure S22. Sequence alignment of Calcitonin-like diuretic hormone 31 (CL-DH31) precursors.

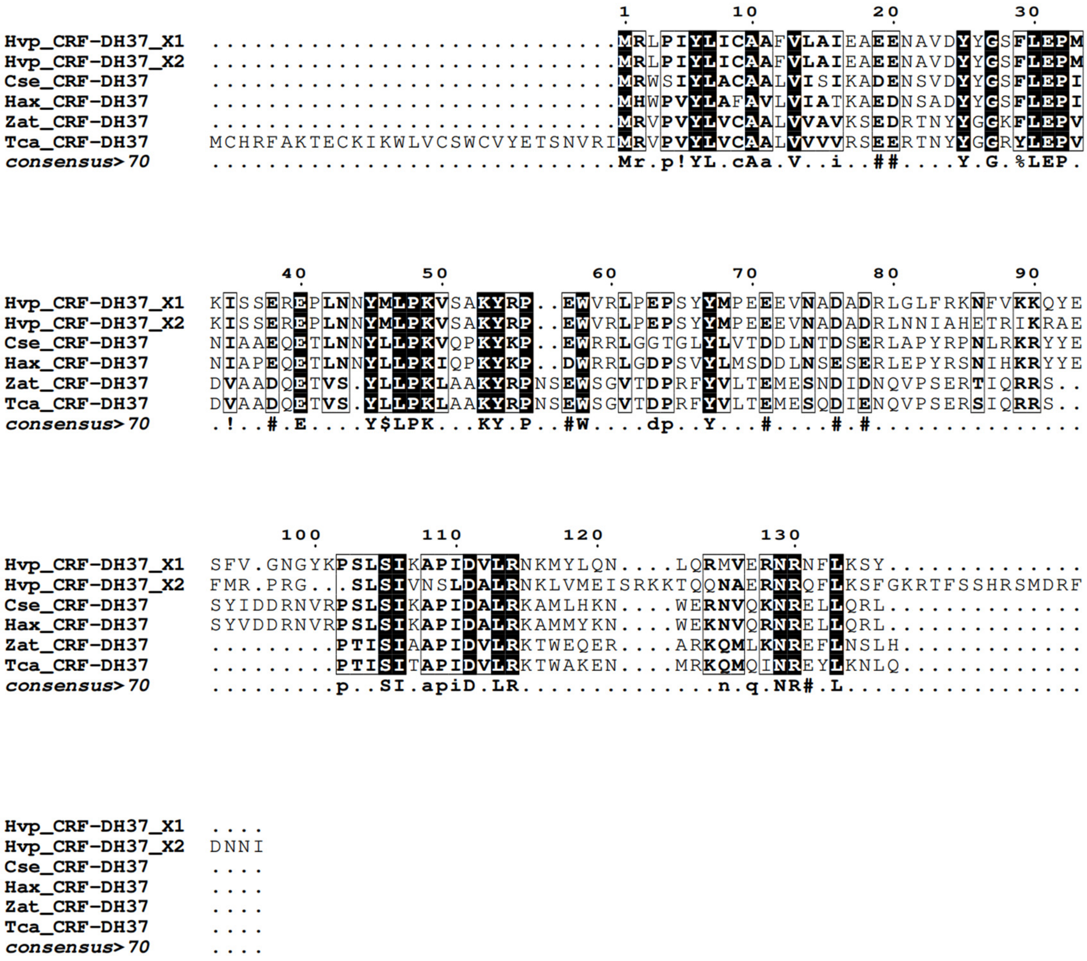

Figure S23. Sequence alignment of Corticotropin-releasing factor-like-diuretic hormone 37 (CRF-DH 37) precursors.

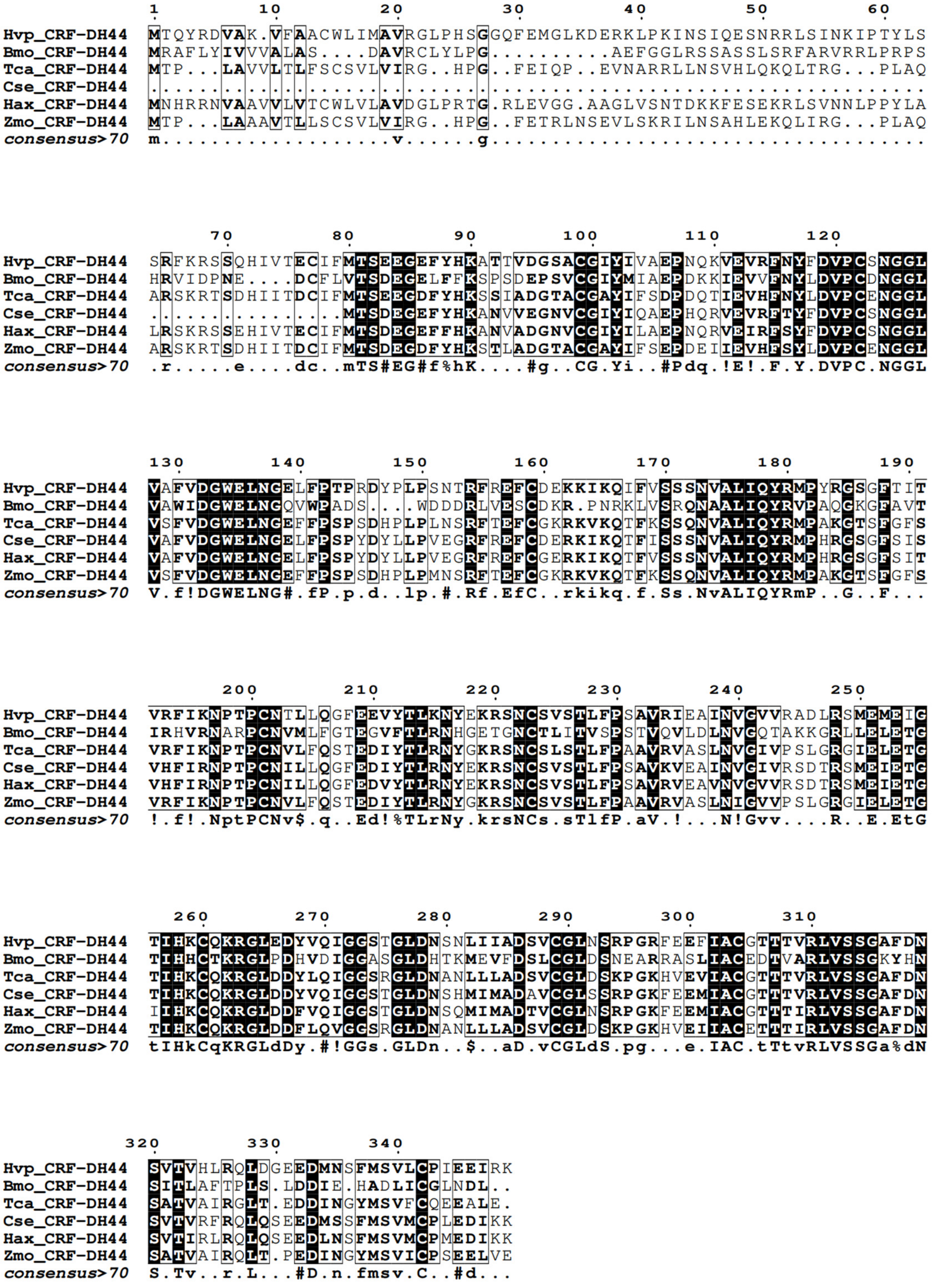

Figure S24. Sequence alignment of Corticotropin-releasing factor-like-diuretic hormone 44 (CRF-DH 44) precursors.

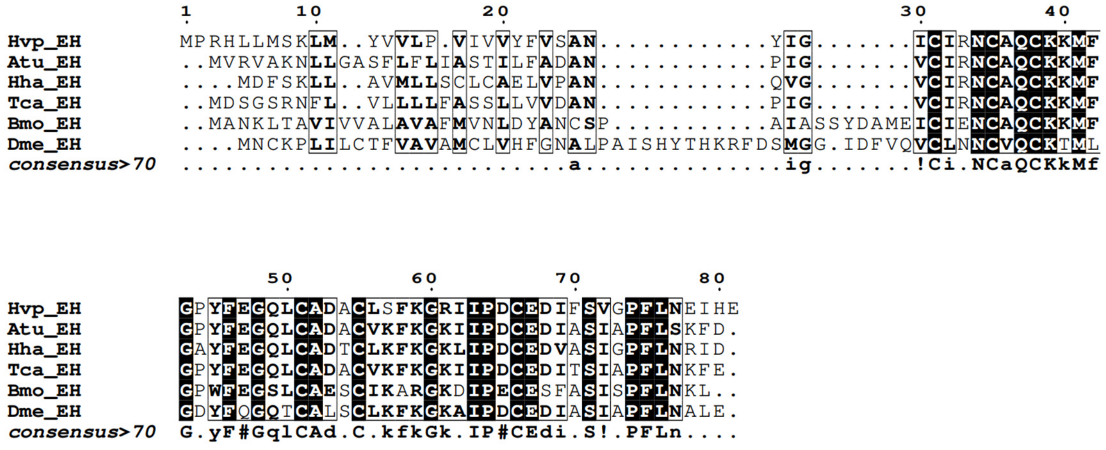

Figure S25. Sequence alignment of Eclosion hormone (EH) precursors.

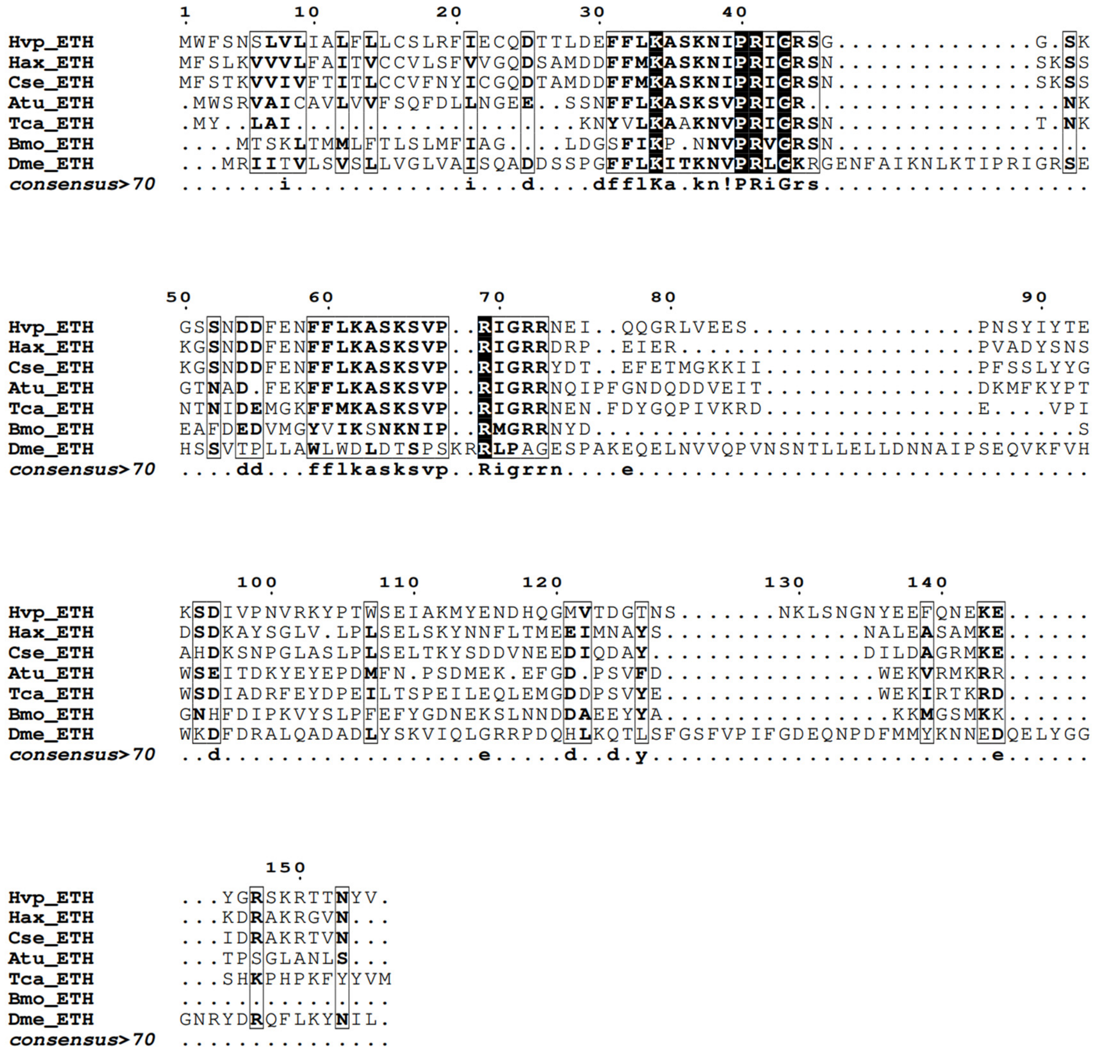

Figure S26. Sequence alignment of Ecdysis triggering hormone (ETH) precursors.

```
                                                                      1
Hvp_FMRF        ......................................................MFIQTLLVL.
Hax_FMRF        MGALAPPLQALCGRLQRIKSYPDKSYPETLVDQRCSVFCTISRYRVCEPSRDSPMYISGLLVL.
Cse_FMRF        ......................................................MYISALLVL.
Atu_FMRF        ......................................................MFFFPLVIL.
Tca_FMRF        ......................................................MVPFAILILT
Dme_FMRF        ........MGIALMFLLALYQMQSAIHSEIIDTPNYAGNSLQDADSEVSPPQDNDLVDALLGND
consensus>70    ......................................................m....llvl.

                10        20        30        40              50        60
Hvp_FMRF        VVVHFTWGYNEDNYPYPDGFDANMNIPEDYDE......QFNDFEPVEKRSH..NFIRFGRAPSE
Hax_FMRF        VLTQYTWAYNDDNYPFSDNYDPNIIPFEDYTD......QEDIFEPAEKRSE..NLGRYEQRRSS
Cse_FMRF        VLTQYTWAYNDDNYPFSDSYDPTLISAEDYAD......QEDIFEPAEKRAN..NIPRYEHRRSS
Atu_FMRF        FLVQSTWGYVDET.YYPGENVDNYLYSEEVTE......NENPNPPQRRNNNNNNFLRFGRSEKK
Tca_FMRF        LIIQLASGYNNED.FYS....DNFDGFEEPSE......EVSDMEVRRRNS...NFLRFGRSGPN
Dme_FMRF        QTERAELEFRHPISVIGIDYSKNAVVLHFQKHGRKPRYKYDPELEAKRRSVQDNFMHFGKRQAE
consensus>70    ........%.dd...y......n....ed..e......q.d...........N..r%......

                                  70        80        90        100
Hvp_FMRF        Q.............EPEPRPAR.........RNDYFVRFGR.SKSDYLRFGRDMAHQRYGRSKDN.
Hax_FMRF        N.............DPDPRPTRGAKM..LRSDYFVRFGR.SKQDFLRFGRDPPHQRLSRSRD..
Cse_FMRF        N.............DPDPRPTRGAKM..LRSDYFVRFGR.SKQDFLRFGRDPNQQRFSRSRD..
Atu_FMRF        YDT.....DYEDYIEDFARPTRSGRT..DKNDHFIRFGR.AGQDFLRFGRS..QVRLHRDRDT.
Tca_FMRF        Y.......EYEDYGEDFARPTRSGKI..EKNDHFIRFGR.SKQDFLRFGRN..QPKATTN....
Dme_FMRF        QLPPEGSYAESDELEGMAKRAAMDRYGRDPKQDFMRFGRDPKQDFMRFGRDPKQDFMRFGRDPK
consensus>70    ..............#...rp.r.........#.FvRFGR..kqD%$RFGRd..........d..

                  110       120       130       140       150       160
Hvp_FMRF        ..YVRFGRSVPEVASGTRSKREATSEAETKRN.SNYLRFGRGNSDFLRYGRSRDEQRMTGD...
Hax_FMRF        ..YLRFGRSLPTPGEHERSKREATFEPDTKRN.SNFLRFGR.NSNFLRFGRTKEDHSQH.....
Cse_FMRF        ..YLRFGRSPPPATENGRYKRDAVFDPEVKRN.SNFLRFGR.NSNFLRFGRNKEDPMQG.....
Atu_FMRF        ..HLRFGRSAPVQEQKSRAKRDTAMQEEYKRGGSNFLRFGR.NSNFLRFGRNPDGVLAMPT...
Tca_FMRF        ..YLRFGR.........RNKRDT...........SNFLRFGR.NSNFLRFGRN............
Dme_FMRF        QDFMRFGRDPKQDFMRFGRTPAEDFMRFGRTPAEDFMRFGR.SDNFMRFGRSPHEELRSPKQDF
consensus>70    ..ylRFGR.........r.krd...........s#%$RFGR.ns#F$R%GR.............

                                                        170       180
Hvp_FMRF        .......................................LQMKLTPEDI.KLL.KFQQLYDSPL
Hax_FMRF        .........................................LELTQEDL.KLLSQLRRFYESPL
Cse_FMRF        .........................................VELTQEDL.KMLGKLRQFYESPL
Atu_FMRF        .......................................SETEITHAQINELLGKIKQMHDSPL
Tca_FMRF        .......................................NESS................YESPL
Dme_FMRF        MRFGRPDNFMRFGRSAPQDFVRSGKMDSNFIRFGKSLKPAAPESKPVKSNQGNPGERSPVDKAM
consensus>70    ............................................................#sp$

                190       200
Hvp_FMRF        IRLLAQLMSSDREKCKNNL.................
Hax_FMRF        SRLFAQLEFQGQRRCESDV.................
Cse_FMRF        WRLFVQLRLEGQERWKSDV.................
Atu_FMRF        LRLLTDLAINARQDGKKFSS................
Tca_FMRF        VQLLSSLLK..KEENKQRIV................
Dme_FMRF        TELFKKQELQDQQVKNGAQATTTQDGSVEQDQFFGQ
consensus>70    ..L...l.....e.......................
```

Figure S27. Sequence alignment of FMRFamide (FMRF) precursors.

```
              1        10        20        30        40        50        60
Hvp_GPA2      MLACWLVLGLLSISHAFFVSSVVAKNVWQQAGCHKVGHSRKISIPDCVEFNMTTNACRGFCESW
Tca_GPA2      MLACWLLFTLLSLSDAFMVKAVTARDAWQKPGCHKVGHTRKISIPECVEFHMTTNACRGFCESW
Tmo_GPA2      MLACWLLFTVLSLSDAFMVTTVNARDAWQKPGCHKVGHTRKISIPECVEFHMTTNACRGFCESW
Ave_GPA2      MLACWLLFTVLSLSNAFMVTTVNARDAWQKPGCHKVGHTRKISIPECVEFHMTTNACRGFCESW
Bmo_GPA2      .....MLLRFIVTLMFLC..QIFAAESWRKPGCHRIGHTRNISIPDCVEFKITTNACRGYCESW
Dme_GPA2      .MGSSQLLVLICCIPWLCDSNSMGKDAWLRPGCHKVGNTRKITIPDCVEFTITTNACRGFCESF
consensus>70  .l...ll..............v.a.#.W..pGCHk!GhtRkIsIP#CVEF..TTNACRG%CESw

                   70        80        90       100       110       120
Hvp_GPA2      AVPSHPIT........NPLQPVTSVGQCCNMMESEPVEARVLCVDGLRTLTFKSALSCACYHCK
Tca_GPA2      AVPSGPKA........TPTQPVTSVGQCCNIMETEPVEARVLCVDGVRTLTFKSAVSCSCYHCK
Tmo_GPA2      AVPSGPKA........TPTQPVTSVGQCCNIMETEPVEARVLCVDGVRTLTFKSAVSCSCYHCK
Ave_GPA2      AVPSGPKA........TPTQPVTSVGQCCNIMDTEPVEARVLCVDGVRTLTFKSAVSCSCYHCK
Bmo_GPA2      SLPSIMLG........FKRHPVTSLGQCCNIMEAEDVPVKVLCLDGERNLIFKSAVSCACYHCQ
Dme_GPA2      SVPSIPMMGSSLSVLFKPPKPVVSVGQCCNMMKSEEIQRRVLCIEGIRNVTFNSALSCSCYHCK
consensus>70  .vPS.p...........p..PVtSvGQCCN.Me.E.!e.rVLCv#G.R.ltFkSA.SC.CYHCk

Hvp_GPA2      KD
Tca_GPA2      KD
Tmo_GPA2      KD
Ave_GPA2      KD
Bmo_GPA2      KE
Dme_GPA2      KD
consensus>70  K#
```

Figure S28. Sequence alignment of Glycoprotein hormone alpha 2 (GPA2) precursors.

```
              1        10        20        30         40        50        60
Hvp_GPB5      MKQLISLQIYIIVISTTVRSQSIIDVESLIPQEP.ETLSCEEMPYTFSVVQTDANGKQCWGAVT
Hax_GPB5      MKYLISWNICFFIISTSVDCQSIIDVESLIPPEEPESLECRERIYTFQVTQTDINGKQCWDALS
Cse_GPB5      MQYLISWNICLFMITTSVVSQ..IDVDSLIPPEE..DLECRERVYTFQVTQTDENGKQCWDALS
Tca_GPB5      ..MLGVQVWLFVGLSALVRCQSIIEAG.LEPLDASGTIECHRRMYTYRVTQTDDNGKQCWDTLS
Ave_GPB5      ............SLSALVNCQSIIEAG.LEPLDASGTIECHRRVYTYRVTQTDENGKQCWDTLS
Bmo_GPB5      ......MLNKINRLSMMTVGLMACICWAFFCVDASMSVRCKLKRHSHKVMQTDLNSRRCWDDVK
Dme_GPB5      .......................MNNGHIVTP.....LGCHRRVYTYKVTQSDLQGHECWDYVS
consensus>70  ..............s..v..q..id...l.p.e......C..r.ytf.VtQtD.#gkqCWd..s

                    70        80        90       100       110       120
Hvp_GPB5      TNACKGRCDSNEISDWRFPFKKSNHPVCVHYGRNRNVVTLRHCEEGALPEAARYEYLEAVGCKC
Hax_GPB5      TIACWGRCDSNEISDWRFPYKKSNHPVCIHYGRNRNVVTLRHCEEGALPEAAHYEYLEAAGCKC
Cse_GPB5      TVACWGRCDSNEISDWRFPFKKSNHPVCIHYGRNRNVVTLRHCEEGALPEAARYEYLEAAGCKC
Tca_GPB5      VMACWGRCDSNEISDWRFPYKKSNHPVCVHYGRNRSVVTLRHCEEGANPSAARYEYLEAAGCKC
Ave_GPB5      VMACWGRCDSNEISDWRFPYKKSNHPVCVHYGRNRSVVTLRHCEEGANPSAARYEYLEAAGCKC
Bmo_GPB5      IVSCWGYCLSYEISDWQFPYKESHHPVCVHGERRHASVKLRNCDPGVEPGTEIYHYVEAVNCRC
Dme_GPB5      VWSCWGRCDSSEISDWKFPYKRSFHPVCVHAQRQLVVAILKNCHPKAEDSVSKYQYMEAVNCHC
consensus>70  ..aCwGrCdSnEISDWrFP%KkSnHPVC!HygRnr.vvtLrhCeega.p.aa.YeYlEA.gCkC

              130       140       150
Hvp_GPB5      QICSSSDTSCEGITYRPHR..SYPVSWGY.......
Hax_GPB5      QICSSSDTSCEGLRYRPQR..SHPQSLGFQLS....
Cse_GPB5      QICSSSDTSCEGLRYRPQR..SHPQSLGFQLI....
Tca_GPB5      QQCSSSDTSCEGLRYRPQR..SHPASLGFRIN....
Ave_GPB5      QQCSSSDTSCEGLRYRPQR..SHPDSLGFRIN....
Bmo_GPB5      QVCSSEDTSCEWLPPDSSLLGGLILKEELEEELE..
Dme_GPB5      QTCSTQDTSCEAPANNEMAGGSRAIMVGADTKNLDY
consensus>70  Q.CSssDTSCEgl.yrp.r..s.p.s.gf.......
```

Figure S29. Sequence alignment of Glycoprotein hormone beta 5 (GPB5) precursors.

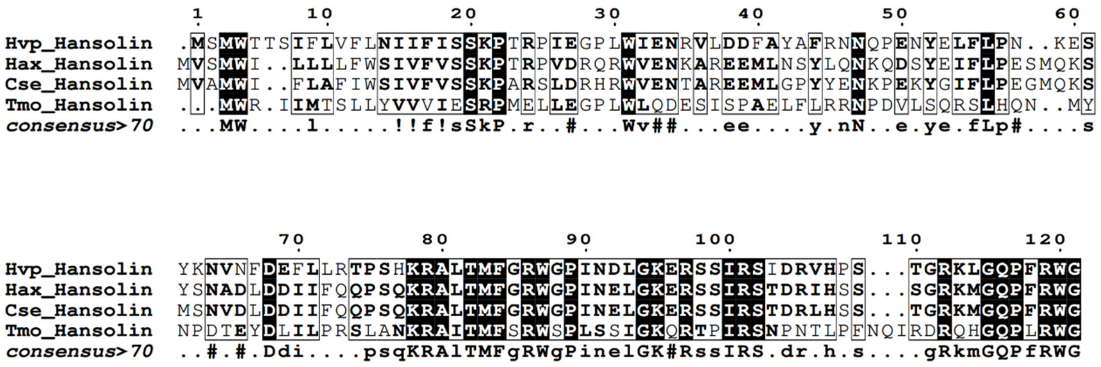

Figure S30. Sequence alignment of Hansolin precursors.

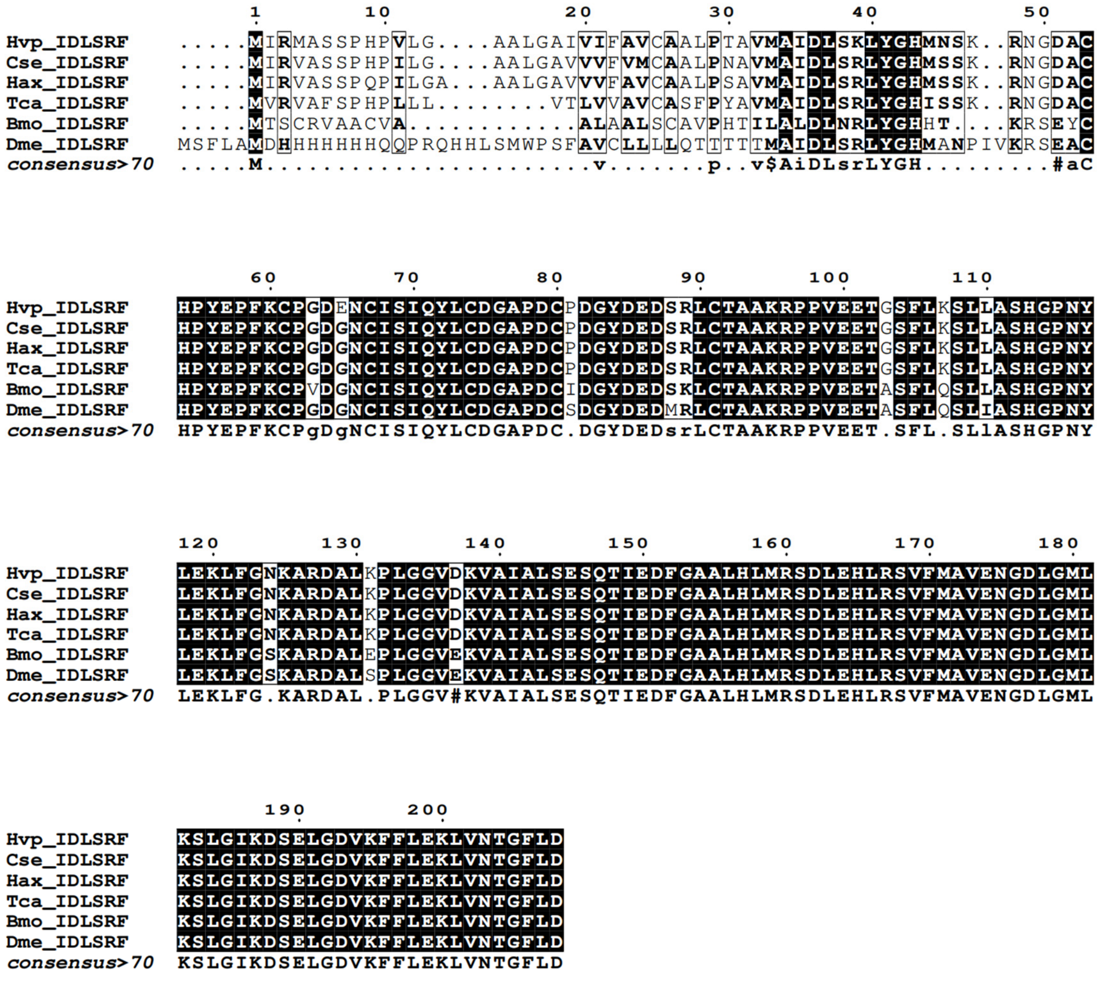

Figure S31. Sequence alignment of IDLSRF-like peptide (IDLSRF) precursors.

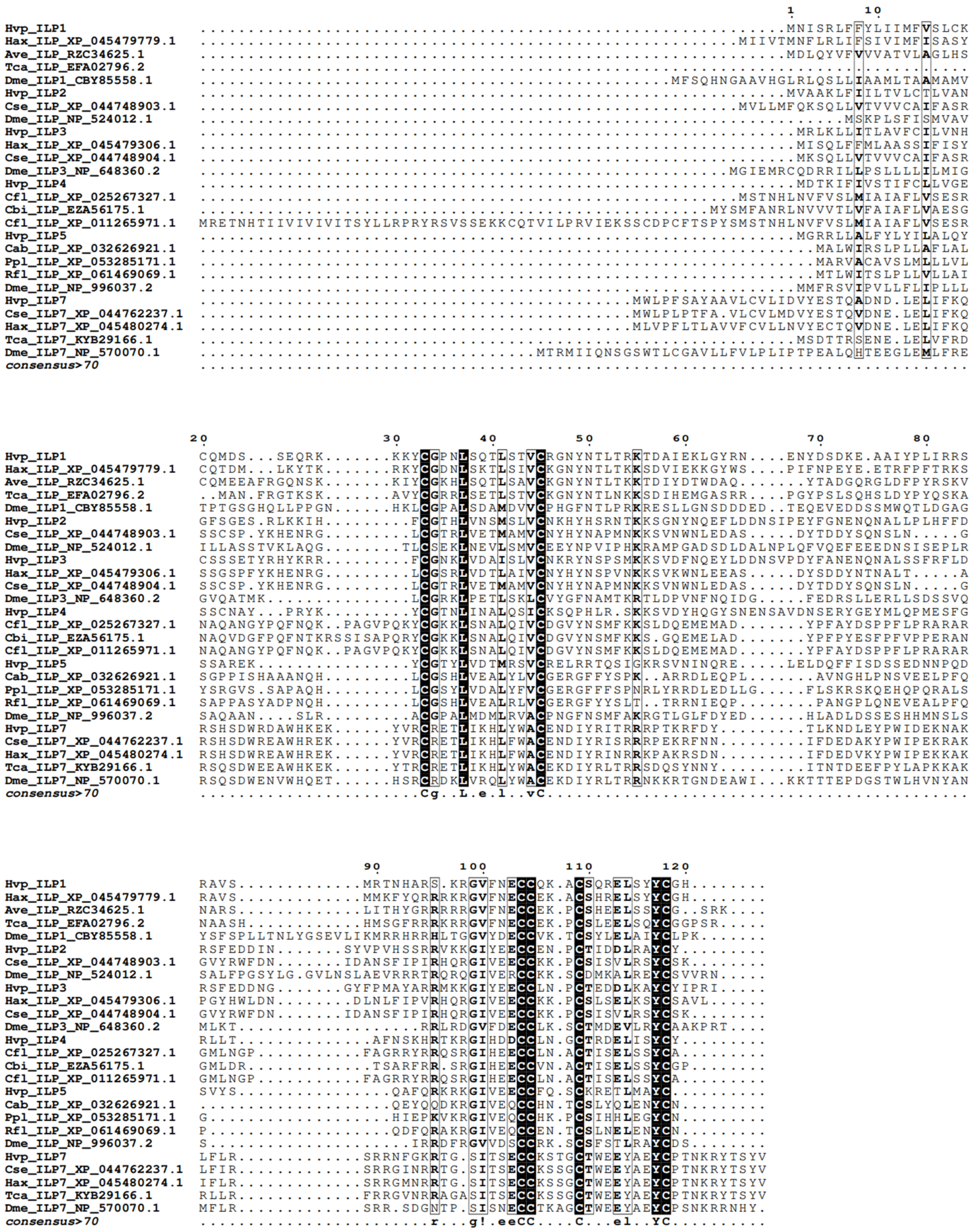

Figure S32. Sequence alignment of Insulin-like peptide (ILP) precursors.

```
                 1        10        20        30        40        50        60
                 .        .         .         .         .         .         .
Hvp_ITP          MNYLSSK.VFTTLLWVCLALILFVSKSLASPADRSPSLVSHRVAKRS.FFDIQCKGVYDRSIFA
Cse_ITP          MNYLSSK.VSTTLLWVCMALVLFLSGSFGSPANRSSDLLSHHLAKRS.FFDIQCKGVYDKSIFA
Hax_ITP          MNYLSSK.VSTTLLWVCMAFIFFFGSSLGNPTNRSSGLLSHHLAKRS.FFDIQCKGVYDKSIFA
Ave_ITP          MNYRSSKNINTQVVWVCMALTLVLQVVNCSPPNRSLMLLSHHLTKRS.FFDIQCKGVYDKSIFA
Tca_ITP          MNYRSSKSISTQAVWVCMVLAVVFQEITSSPAGRSPAFLPHHFTKRS.FFDIQCKGVYDKSIFA
Dme_ITP          ........MCSRNIKISVVLFLVLIPIFAALPHN.....HNLSKRSNFFDLECKGIFNKTMFF
consensus>70     mny.ssk.v.t...w!cm.l.........p..rs.....H...KRS.FFDi#CKG!%#ksiFa

                        70        80        90       100       110       120
                         .         .         .         .         .         .
Hvp_ITP          RLDRICEDCYNLFREPQVHSLCRKNCFTTDYFKGCLDTLQLSDELEQIQTWIKQLHGAEV....
Cse_ITP          RLDRICEDCYNLFREPQVHSLCRRNCFTTDYFKGCLDTLQLSDELQQIQTWIKQLHGADPGV..
Hax_ITP          RLDRICEDCYNLFREPQVHSLCRKNCFTTDYFKGCLDTLQLSDELQQIQTWIKQLHGADPGV..
Ave_ITP          RLDRICEDCYNLFREPQLHSLCRKNCFTTDYFKGCLETLQLSDEETQIQTWIKQLHGAEPGV..
Tca_ITP          KLDSICEDCYMLFREPQLHNLCRKNCFTTDYFKGCIDTLQRSDEEAQIQLWIKQIRGAELGGLG
Dme_ITP          RLDRICEDCYQLFRETSIHRLCKQECFGSPFFNACIEALQLHEEMDKYNEWRDTLGRK......
consensus>70     rLDrICEDCYnLFREpq.H.LCr.#CFttd%FkgC.#tLQls#E..qi#.Wikql.gae.....

Hvp_ITP          .........
Cse_ITP          .........
Hax_ITP          .........
Ave_ITP          .........
Tca_ITP          PSASPPNTS
Dme_ITP          .........
consensus>70     .........
```

Figure S33. Sequence alignment of ion transport peptide (ITP) precursors.

```
                 1        10        20        30        40        50        60
                 .        .         .         .         .         .         .
Hvp_ITG          MKVLLSLLAICLVGQHKVDAWGGLFNRFSPEMLANMGYGG....HGGFIQRGGGEGDEGILEEY
Hax_ITG          MKSLITLLALCLVGQQTAYAWGGLFNRFSPEMLANMGYGG....HGGYMQRSG.EGDEGILEEY
Cse_ITG          MRSLISLLAICLVGQQTAHAWGGLFNRFSPEMLANMGYGG....HGGFIQRAG.EGDEGILEEY
Zmo_ITG          MRSLIILFMACLLG.HKAHAWGGLFNRFSPEMLANMGYGG....HGGFIQRTG.EGDEGILEEY
Bmo_ITG          MTATAILVLGCLSG...AHAWGGLFNRFSSDMLANLGYGRSPYRHYPYGQVEPDEAYEALENNR
consensus>70     M..l..L...CL.G...a.AWGGLFNRFSp#MLAN$GYGg....Hgg%.Qr.g.EgdEgil##y

                        70        80        90       100       110       120
                         .         .         .         .         .         .
Hvp_ITG          TNDVEDEP..CYGKRCTANEHCCPGSVCVDVDGVIGSCIFAYG.RRVGELCRRDTDCESGLVCA
Hax_ITG          TNDVEDEP..CYGKRCTANEHCCPGSVCVDVDG.VGSCLFAYG.RRVGELCRRDTDCESGLVCA
Cse_ITG          SSDVDEEP..CYGKRCTANEHCCPGSVCVDVDG.VGSCLFAYG.RRVGELCRRDTDCESGLVCA
Zmo_ITG          ASEGDEEP..CYGKRCTANEHCCPGSVCVDVDG.VGSCLFAYG.RRVGELCRRDSDCESGLVCA
Bmo_ITG          ISNVIDEPAHCYSSPCVTNGDCCGGLLCLETDD.GGRCLSAFAGRKLGEICNRENQCDAGLICE
consensus>70     ..#ve#EP..CYgkrCtaNehCCpGsvCv#vDg.vGsClfA%g.RrvGElCrR#.#C#sGL!Ca

                       130       140       150       160       170       180
                         .         .         .         .         .         .
Hvp_ITG          EAEPGTLTRVCRPPVHQDKQYSEVCNMSNECDISRGLCCQLQRRHRQAPRKVCSYFKDPLVCIG
Hax_ITG          EAEPGTMTRVCRPPVHQEKQYSELCNMSSECDISRGLCCQLQRRHRQAPRKVCSYFKDPLVCIG
Cse_ITG          EAEPGTMTRVCRPPVHQEKQYSELCNMSSECDISRGLCCQLQRRHRQAPRKVCSYFKDPLVCIG
Zmo_ITG          EAEPVVSTRVCRPPVHQDKQYSEPCNMSSECDISRGLCCQLQRRHRQAPRKVCSYFKDPLVCIG
Bmo_ITG          EAAPGEMH.ICRPPSTGRKQYNEDCTTSSECEITRGLCCIMQRRHRQKSRKSCGYFKEPLVCIG
consensus>70     EAePg.mtr!CRPPvhqdKQYsE.CnmSsEC#IsRGLCCq$QRRHRQapRKvCsYFK#PLVCIG

                    190       200       210
                      .         .         .
Hvp_ITG          PVATDQIKSSVQHTAGEKRLTGFSNFKR..PMH
Hax_ITG          PVATDQIKNSIQHTAGEKRLTGFTSFKRPLPMH
Cse_ITG          PVATDQIKNSVQHTAGEKRLTGFSSFKRPLPMH
Zmo_ITG          PVASDQIKSTIQHTAGEKRLTGLATFKR..PMH
Bmo_ITG          PVAIDQIREYVEHTTGEKRIGAYR.......LH
consensus>70     PVA.DQIk..!#HTaGEKRltgf..fkr..p$H
```

Figure S34. Sequence alignment of ITG-like (ITG) precursors.

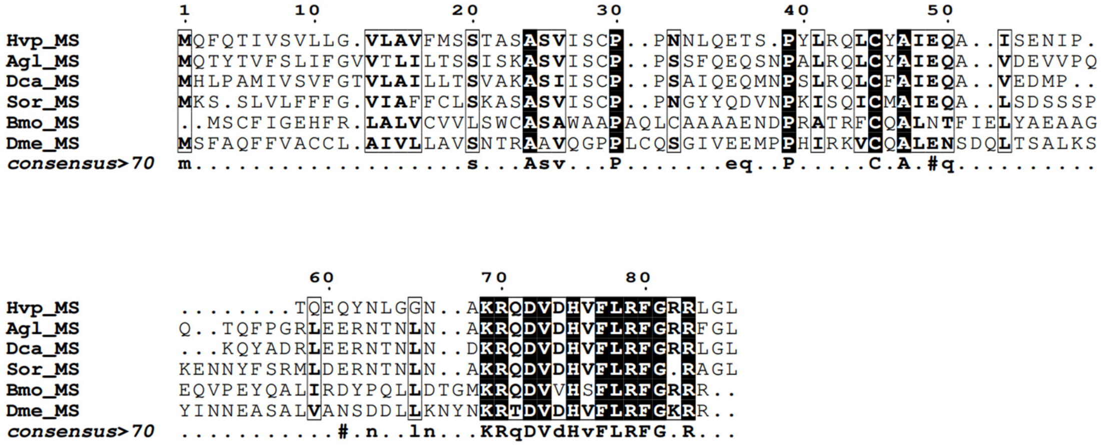

Figure S35. Sequence alignment of Myosuppressin (MS) precursors.

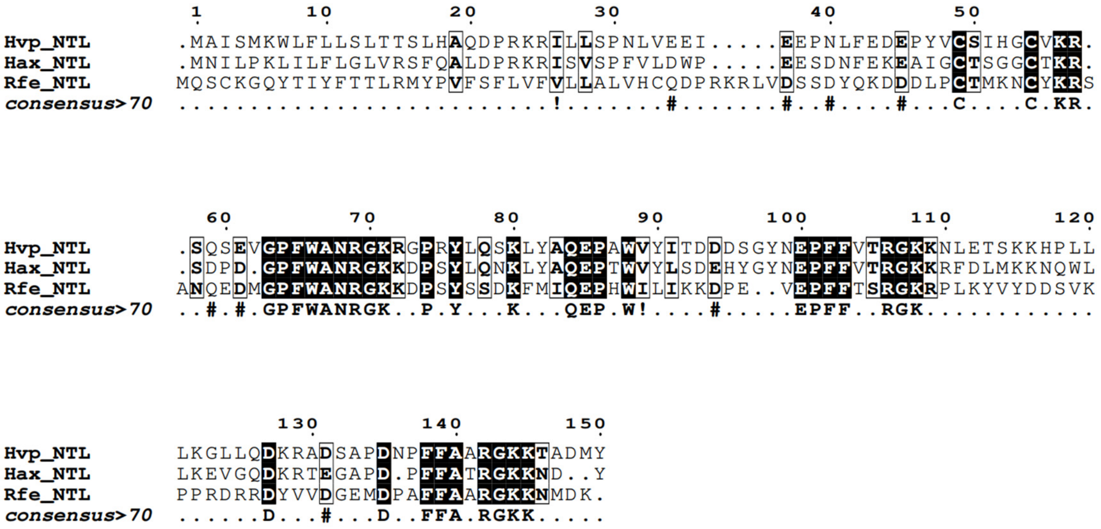

Figure S36. Sequence alignment of Natalisin (NTL) precursors.

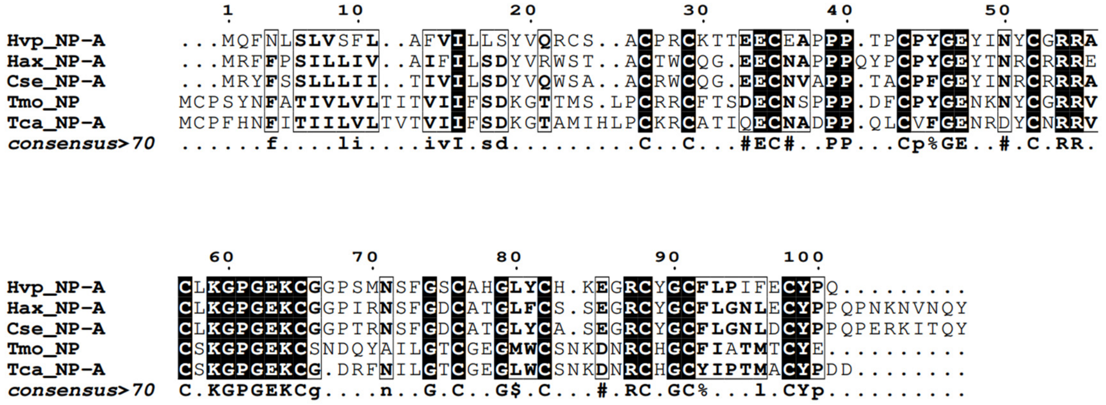

Figure S37. Sequence alignment of Neuroparsin A (NPA) precursors.

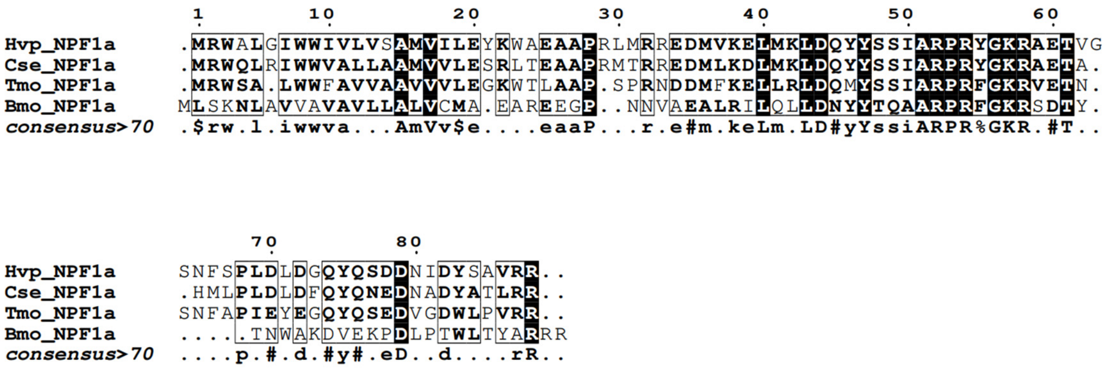

Figure S38. Sequence alignment of Neuropeptide F 1a (NPF1a) precursors.

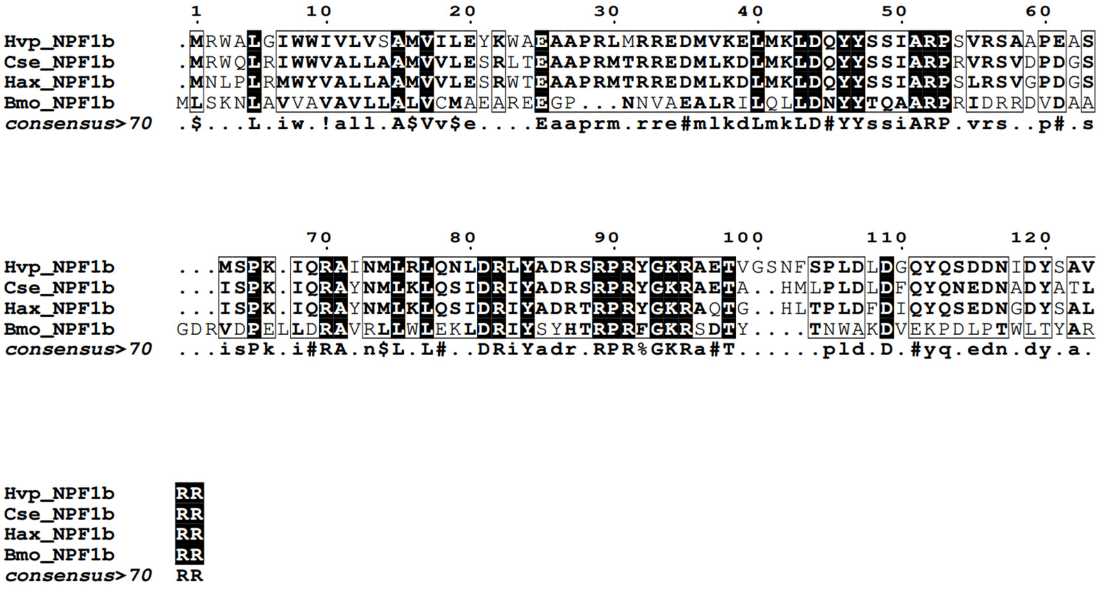

Figure S39. Sequence alignment of Neuropeptide F 1b (NPF1b) precursors.

```
                        1         10         20         30         40
Hvp_NPLP1_X1   ......MAFHHFRLRFAI.GFFLAIL.....LMVQSDESCNL...DFEKTISTLFAPHEPPSLQ
Hvp_NPLP1_X2   ......MNSQTISMMVFR.IFIFTML.....SAVQSDESCNL...DFEKTISTLFAPHEPPSLQ
Cse_NPLP1      ......MMVSGFRKPFAV.VFLSVFI.....IMVQSEGTCNM...DFERTLEILFAPQEQPSPQ
Hax_NPLP1      .......MVSRFPLKLLL.VFLSVYV.....CMVQSDEPCNL...DFERTLQTLFAPQEQPSPQ
Tma_NPLP1      ......MVFGTPKFLFGTGVLMFALF.....FVVKSDESCDI...EIENTLKTLLKPQEYPSMQ
Bmo_NPLP1      ...MKGRSPTGKRRGCFLLIVLFAVI....................FSAYVEQVLSLPTEP...
Dme_NPLP1      MQAVLQSAHSSRRLMLLLSMLLNAAIQPRSIIVSATDDVANVSPCEMESLINQLMSPSPEYQLH
consensus>70   ...................v.l...........v.sde.cn....dfe.t...l..p.e.ps.q

               50        60        70                  80        90        100
Hvp_NPLP1_X1   ILTLRRYLLKILQNMIVKAEEIEGWN...........GNTYPKRNIEALARAGYLHTLPEDEED
Hvp_NPLP1_X2   ILTLRRYLLKILQNMIVKAEEIEGWN...........GNTYPKRNIEALARAGYLHTLPEDEED
Cse_NPLP1      IRSLRRYLISIFHNMVARAEEIEGWN...........TYVYPKRSLEALARAGYLHTLP.DDED
Hax_NPLP1      IRSLRRYFISILHSMVARAEEAEGWN...........TYVYPKRSLEALARAGYLHTLP.DEED
Tma_NPLP1      NLALRKDLLRRLQQALDRVVEDDDMNYKRSISSLAQWGNLPGKRNLEALARAGYIRTLPNPDEE
Bmo_NPLP1      .....................ENQWP.............TFPRRNIATLAKNGYLRNSG.....
Dme_NPLP1      ASALRNQLKNLLRERQLAVGEEQPLG.......EYPDYLEEDKRSVAALAAQGLLNAPK.....
consensus>70   ...lr..l...l.n......e.#.wn..............ypkR..eaLAraGyl.tlp..eed

                   110       120       130       140        150
Hvp_NPLP1_X1   NQTEIHNKRSLESLVKNGQLPAHHYTEEEGIKRSIESLARNGELK..RDIQRMLEELYN.....
Hvp_NPLP1_X2   NQTEIHNKRSLESLVKNGQLPAHHYTEEEGIKRSIESLARNGELK..RDIQRMLEELYN.....
Cse_NPLP1      NQTDSNDKRSLSALAKNGQFPLHQFRDEESYKRSSGPPN.SGDIS..KEIQKMLDDLYN.....
Hax_NPLP1      NQTDSNDKRSLSALAKNGQLPVHRLRDEESFKRSGTPVS.TNDIN..KEMQKMLDDLYN.....
Tma_NPLP1      ...DPNNKRSLSTLAKNDQLPTFQNNES...KRGIESLARNGELHNRRDIQELLDELYDKRNIG
Bmo_NPLP1      ...ANSYKRSISILAKNGMLPTYRSPYVGTDKQEHEDESQE.......................
Dme_NPLP1      ........RSLATLAKNGQLPTAEPGEDYGDADSGEPSEQKRYIGSLARAGGLMTYGKRNVG..
consensus>70   ...d..nkRSl..LaKNgqlP.....ee...krs.e......e.....d.q.mldelyn.....

                                160       170       180       190
Hvp_NPLP1_X1   .................DMTYAQ......DKRNLASIARDGGFAG.....KRNAAALLKNDRYL
Hvp_NPLP1_X2   .................DMTVAQ......DKRNLASIARDGGFAG.....KRNAAALLKNDRYL
Cse_NPLP1      .................NIARDGN.....TKRNLASIARDGGFAG.....KRNAAALLKNDRYL
Hax_NPLP1      .................NLAQVET.....TKRNIASIARDGGFLG.....KRNVAALLKNDRYL
Tma_NPLP1      SLARNFNFPSYGKRYLGSLVRNGESQY.SGKRNIASIARDGGFVG.....KRNVAALLRQDDYL
Bmo_NPLP1      ..............................KRNLASIARLRSYSA....MKRNIQALARDGYRM
Dme_NPLP1      .................TLARDFQLPIPNGKRNIATMARLQSAPSTHRDPKRNVAAVARYNSQH
consensus>70   ..................l...........KRN.AsiARdggf.g.....KRN.aAll.nd.yl

                   200        210        220        230        240
Hvp_NPLP1_X1   NRMLGQERD......DKRNIASLKAN.....YKPKYKREITKR.QADYSGNEL.......EYPV
Hvp_NPLP1_X2   NRMLGQERD......DKRNIASLKAN.....YKPKYKREITKR.QADYSGNEL.......EYPV
Cse_NPLP1      SHMLNQV.D......SKRNIASLKAS.....YKPRYKRELSN...ADDYEDE........EYQN
Hax_NPLP1      SNMLN..........GKRNIASVKAS.....YKPRYKREMND...LDYYEDE........DYQN
Tma_NPLP1      NEQNNDEKDDPQVNSEKRNIASIKAQ.....YPGQFTRAVRSKRQTTYYEGENG....EFSLPV
Bmo_NPLP1      GRGQYNTQN......NKRNIAALARNG....LLHKKDEINGDEYYFPFYQNPIP.....PLSEI
Dme_NPLP1      GHIQRAGAE.......KRNLGALKSSPVHGVQQKREDEEMLLPAAAPDYADPMQSYWWYPSYAG
consensus>70   .....q..d.......KRNiaslka......y...y.re........yy.ne.........y..
```

Figure S40. Sequence alignment of Neuropeptide-like precursor 1 (NPLP1) precursors.

```
              1         10        20                        30        40        50        60
Hvp_OK_A      MLPTFALTILAVSAVTSTAIP...RKGEYLRDLALFLQPAMERETASSLKKRTYGSLSLGGSYG
Hax_OK_A      MMIHVTFTLLALMVVFSSGVPMFNRKEEYLRELAAIVQPAKD...NTLLKKRTYGSLSLGGSYG
Cse_OK_A      ..............................................................
consensus>70  ..............................................................

                    70        80        90        100       110       120
Hvp_OK_A      TAKRGDLSLLRLGSSFGRLARS.DIRLDDYGILPHGYDP.LINSYSGNKYSPPRILEDTSVEDY
Hax_OK_A      TSKRGDLSLLRLGSGFGRISRGGDIRNDDLEVVPRHYEAMIPETYSRNKNIIPHIFVESGNLDD
Cse_OK_A      ......MSLLRLGSGFGRTSRG.VVRTDDIEVVPRKFEE.IPESYLRNRNNVPRIFVEPASLNE
consensus>70  ......$SLLRLGS.FGR..R...!R.DD..!.P..%#....#.Y..N....P.I..#....#.

                      130        140       150
Hvp_OK_A      PIGKN....GRS.RNSGDSFMKQFPSEEFNKED
Hax_OK_A      LFESDKFLSGSGNRRASEYSSKTFPSEDFNQAD
Cse_OK_A      LFDSDKFVSGSSNGRTSEYGSRTFPSEEFNQAD
consensus>70  ....#....G.......#.....FPSE#FN..D
```

Figure S41. Sequence alignment of Orcokinin A (OK A) precursors.

```
             1        10        20        30                  40
Hvp_OK_B     MLPTFALTILAVSAVTSTAIP...RKGEYLRDLALFY......QPSPLLCNSLSN.........
Hax_OK_B     MMIHVTFTLLALMVVFSSGVPMFNRKEEYLRELAAIDKNGESNQPQNFVEHHFSDDEDEGQREK
Cse_OK_B     MMINVTFTLLALLAVYTSCVPMYNRREEYLRELAAID......KTDAEHSKNMKKD........
consensus>70 M$.....T.LA...V....!P...R..EYLR#LA.............................

                50        60        70        80        90
Hvp_OK_B     .DYEILLNDIDNE.......NED.KIYRTADNSMDLS.......YPDMNNLFEKKSLDRIGGAG
Hax_OK_B     HDSRVFIITIDNDDILRTLLKEDIQLHRFLEEESDLNGFVDKRTEPSSGDLLRKRALDSIGGGA
Cse_OK_B     ......VLDSDNE.......NGNGRIFVIN.....LN...........NDLLLKKIIDDEE...
consensus>70 .........DN#.........#...........L.............#L..K...D......

                              100       110       120       130       140
Hvp_OK_B     LLG...........KRALDGIGGGALLGKRALDRIGGGALLGKRALDRIGGGALLGKRALDGIG
Hax_OK_B     LLGKRSSDGEENLRKRALDSIGGGALLGKRSLDSIGGGALLGKRALDSIGGGALLGKRALDSIG
Cse_OK_B     ...............DAILHAISDGDIPRNDIVDELSVGVMTKKDSSS..SNMDMLRKRALDSIG
consensus>70 ................L..I..G........D....G.$..K.........$L.KRALD.IG

             150       160       170       180       190       200       210
Hvp_OK_B     GGALLGKRALDRIGGGALLGKRALDGIGGGALLGKRALDGIGGGALLGKRALDGIGGGALLGKR
Hax_OK_B     GGALLGKRALDSIGGGALLGKRALDSIGGGALLGKRALDSIGGGALLGKRALDSIGGGALLGKR
Cse_OK_B     GGALLGKRALDSIGGGALLGKRALDRIGGGALLGKRALDSIGGGALLGKRSQE......FLEK.
consensus>70 GGALLGKRALD.IGGGALLGKRALD.IGGGALLGKRALD.IGGGALLGKR..#.......L.K.

                                 220                230       240       250
Hvp_OK_B     SYD...............NEPTMVR..RALDSIGGALLGKRALDSIGGGLIGKRRLDNFD.E
Hax_OK_B     ALDSIGGGALLGKRDPDSNERVALSEKRALDSING...EEKRSLDGIGGGALIGKRALDSIGGG
Cse_OK_B     ...........................RALDSIGGALLGKRALDSIGGGALLGKRSSQNSN..
consensus>70 ...........................RALDSI.G.....KR.LD.IGGG.L.GKR..#.....

             260       270         280
Hvp_OK_B     NIFTEREFEEDDG..SRISQENDKNTSN...
Hax_OK_B     ALLGKRASHSRFGGHSRVYPEIDERELLFIE
Cse_OK_B     .MFLV..........................
consensus>70 ...............................
```

Figure S42. Sequence alignment of Orcokinin B (OK B) precursors.

```
                                           1      10        20        30
Hvp_PDF      ..............................MRSITA.EHPISLIQILALGVILGCALAYPSPA
Cse_PDF      MNQGVFKRISGLFSFSSASKTAIIQLHLFVSMWWLSVKEWRLSFICPLVLVILFGCTIAYPSPG
Hax_PDF      ..............................MRSFLLKENRIAMTCLLVLVVISSSALAYPSPG
consensus>70 ..............................M......E........L.L.!......AYPSP.

                  40        50        60        70        80        90
Hvp_PDF      DDYLISEKEYSSPGAHQLASWIASQMRPKEITVIKELPIIPYRLPLQGKRNSEVTNAIIGSEET
Cse_PDF      EDYVLTEKEYSSPGAHQLASWLTAQRRPKEITVIKDLPVIPYRLPIQGKRNSEVTNAIIRSEET
Hax_PDF      EDYILTEKEYSSPGAHQLASWLTAQRRPKEITVIKDLPVIPYRLPIQGKRNSEVTNAIIRSEET
consensus>70 #DY...EKEYSSPGAHQLASW...Q.RPKEITVIK#LP!IPYRLP.QGKRNSEVTNAII.SEET

               100
Hvp_PDF      QKMYRDGRK
Cse_PDF      QRMYREGRK
Hax_PDF      QRMYREGRK
consensus>70 Q.MYR#GRK
```

Figure S43. Sequence alignment of Pigment-dispersing factor (PDF) precursors.

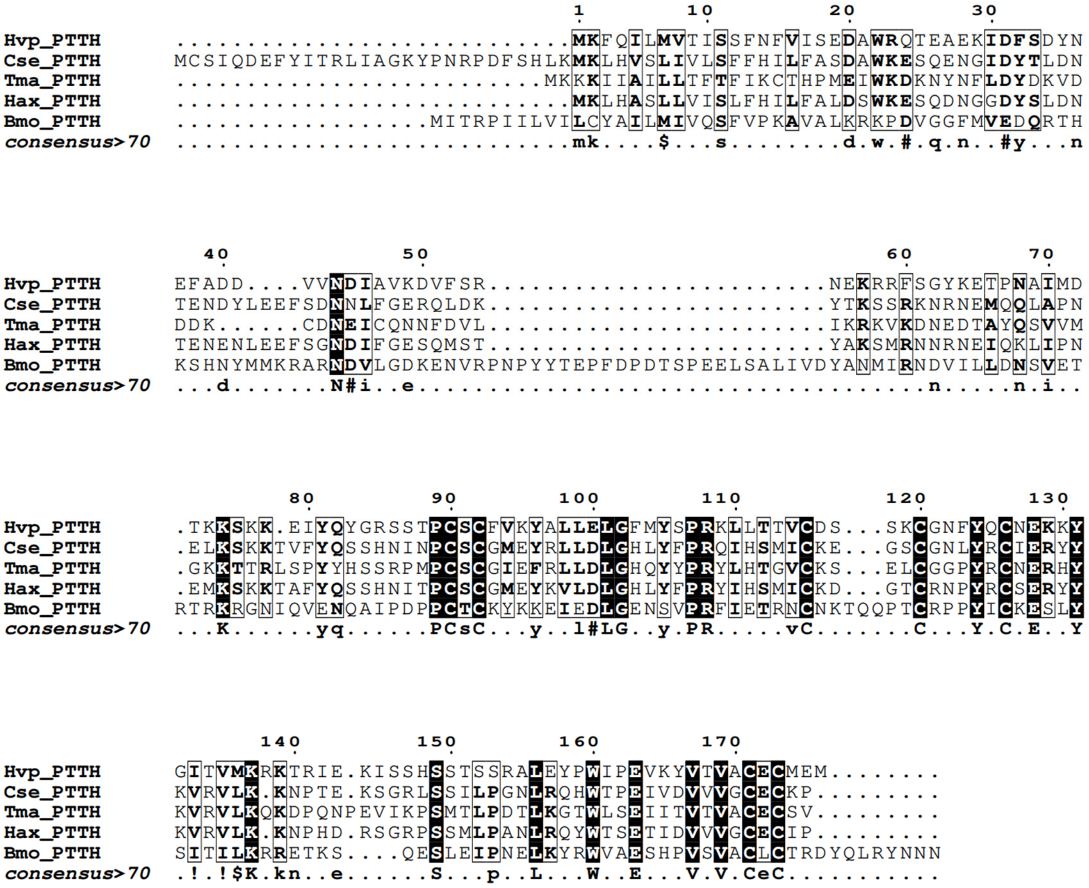

Figure S44. Sequence alignment of Prothoracicotropic hormone (PTTH) precursors.

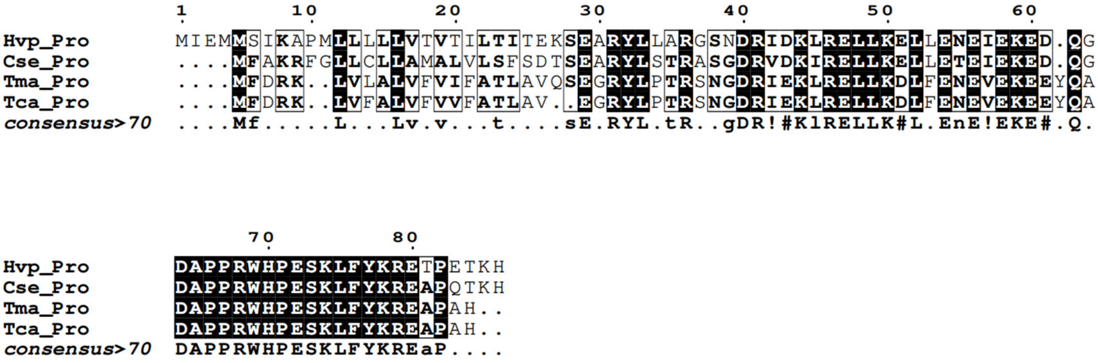

Figure S45. Sequence alignment of Proctolin (Pro) precursors.

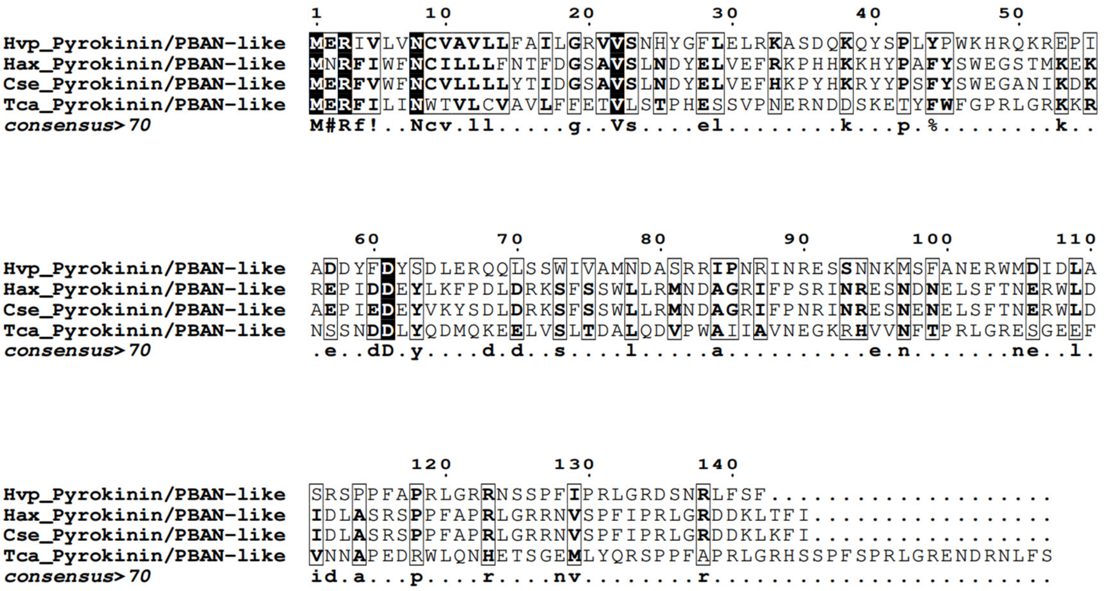

Figure S46. Sequence alignment of Pyrokinin (PK)/phermone biosynthesis activating neuropeptide like (PBAN-like) precursors.

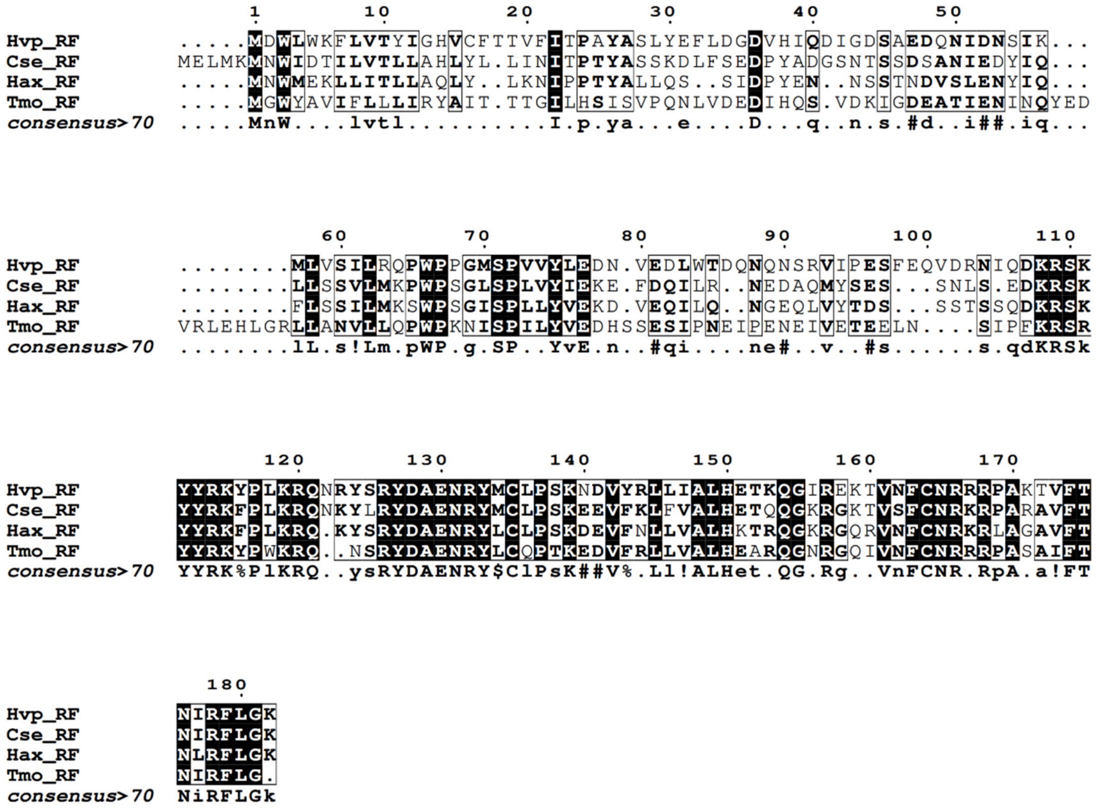

Figure S47. Sequence alignment of RFLamide (RF) precursors.

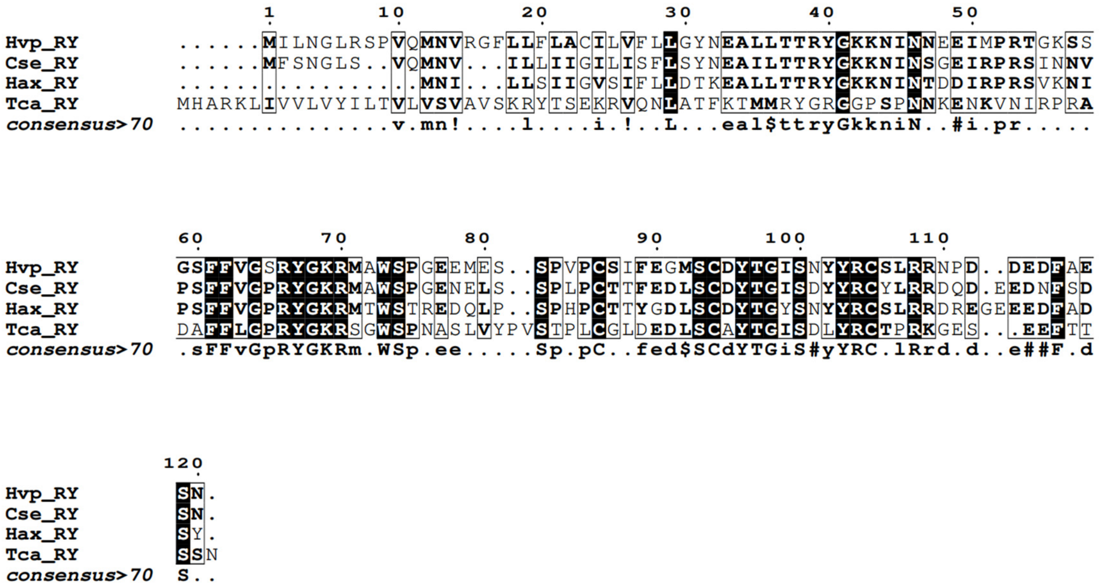

Figure S48. Sequence alignment of Ryamide (RY) precursors.

```
                  1        10        20        30         40        50
Hvp_sNPF       ...MRSSHKMTYLCGIIWVILIVATITTSAPTYGEMDNNIQ..ELLDALYQQDLLNDRFS..GV
Hax_sNPF       ...MCSQLKMKYICSIACIFFILAALANSAPAYGDMNNNIR..ELLDALYQRDMLYDRFN..GI
Cse_sNPF       ...MQFPVKMRYFFSISCIFLILAALANTAPAYGDIDNNIR..DLLDALYQRDMLYDRFN..GV
Tca_sNPF       ...MQRYSAMKCLCAVTCIMIVVATVTSAAPSYADYDNNIR..DLWEILLQKEAMDDKFAPGGP
Bmo_sNPF       ..MSRSFVFALALFGMVAVLPLPAANAQALSNYDASPATFESRNNWDALGGLYALLAQHDALGG
Dme_sNPF       MFHLKRELSQGCALALICLVSLQMQQPAQAEVSSAQGTPLS..NLYDNLLQREYAGPVVF..PN
consensus>70   ...m..............i....a.....a..y..........#l.#.L.q.d.l.......g.

                60        70                                         80
Hvp_sNPF       HQVVRKAGRSPSLRLRFGR.................................RSDPALS.....
Hax_sNPF       HQVERKAGRSPSLRLRFGR.................................RSDPELS.....
Cse_sNPF       HQVVRKAGRSPSLRLRFGR.................................RSDPELS.....
Tca_sNPF       HQMVRKSGRSPSLRLRFGR.................................RSDASMT.....
Bmo_sNPF       HALARKSVRSPSRRLRFGR.................................RSDPDMP.....
Dme_sNPF       HQVERKAQRSPSLRLRFGRSDPDMLNSIVEKRWFGDVNQKPIRSPSLRLRFGRRDPSLPQMRRT
consensus>70   Hq..RK..RSPSlRLRFGR.................................RsDp.$......

                        90
Hvp_sNPF       .....PGAAYLMAQQAQA....DEN.......................................
Hax_sNPF       .....PGVAFLMAQQP......DDN.......................................
Cse_sNPF       .....PGEAFLMAQQA......EEN.......................................
Tca_sNPF       .....PEAAFMMAQAVDH....ETN.......................................
Bmo_sNPF       .....PQAPIDEMDELLSLREVRTPVRLRFG.........RRSDERAVPHIFPQEEQDRAVRAP
Dme_sNPF       AYDDLPERELTLNSQQQQQLGSEPNFDLGADYDGLYERVVRKPQRLRWGRSVPQFESNNADNEQ
consensus>70   .....P.....m.qq.......e.n.......................................

Hvp_sNPF       ................................................................
Hax_sNPF       ................................................................
Cse_sNPF       ................................................................
Tca_sNPF       ................................................................
Bmo_sNPF       SMRLRFGRRSDNNMFLMPYESALPKEVKASGSVEDDRQQE........................
Dme_sNPF       IERSQWYNSLLNSDKMRRMLVALQQQYEIPENVASYANDEDTDADLNNDTSEFQREVRKPMRLR
consensus>70   ................................................................

Hvp_sNPF       ............................
Hax_sNPF       ............................
Cse_sNPF       ............................
Tca_sNPF       ............................
Bmo_sNPF       ............................
Dme_sNPF       WGRSTGKAPSEQKHTPEETSSIPPKTQN
consensus>70   ............................
```

Figure S49. Sequence alignment of short neuropeptide F (sNPF) precursors.

```
                    1        10        20        30        40        50        60
Hvp_SIF        ....MMSSKSATLFIGVLFISLLLFNVGASYRKPPFNGSIFGKRGNTLEYDTAGKTLSAMCEIA
Cse_SIF        MKTLTMFSKSFITFF.VLIVAFLVMTADATYRKPPFNGSIFGKRGTAIEYE.ASKALSAMCEIA
Atu_SIF        MN...SKATAIVIFA.LAIICCLTFS.EATYRKPPFNGSIFGKRGSTNEYDSASKALSAMCEIA
Tca_SIF        MQLALAKVFSVCIVV.IILTSWIEMT.EATYRKPPFNGSIFGKRGATIEYDSASKALSAMCEIA
Bmo_SIF        .......MRADLIYFMFLVIILTLATIEATYRKPPFNGSIFGKR.NNVENDSSGRAIAALCEIT
Dme_SIF        ...MALRFTLTLLLVTILVAAILLGSSEAAYRKPPFNGSIFGKR.NSLDYD..SAKMSAVCEVA
consensus>70   ...........................eA.YRKPPFNGSIFGKR....#y#.....lsAmCE!a

                     70
Hvp_SIF        SEACQAWFPNQEKK
Cse_SIF        SEACQAWFPNQERK
Atu_SIF        SEACQTWFPTQEK.
Tca_SIF        SEACQTWFPSQEK.
Bmo_SIF        TETCQAWYQALESQ
Dme_SIF        MEACPMWFPQNDSK
consensus>70   .EaCq.W%p.q#..
```

Figure S50. Sequence alignment of SIFamide (SIF) precursors.

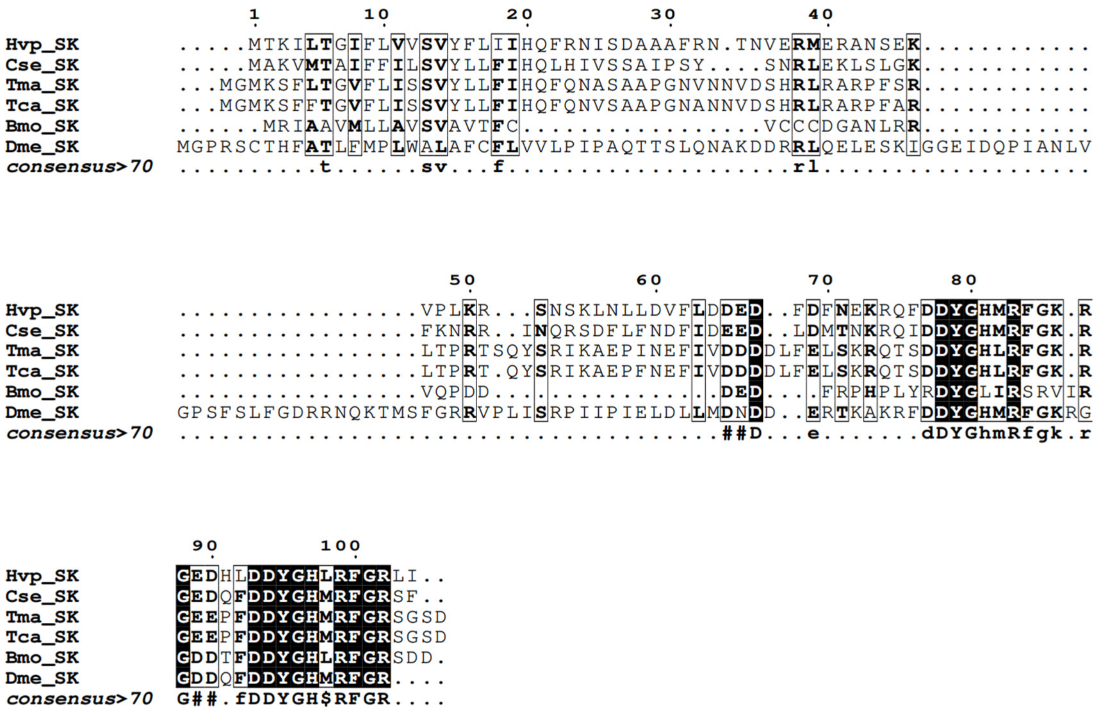

Figure S51. Sequence alignment of Sulfakinin (SK) precursors.

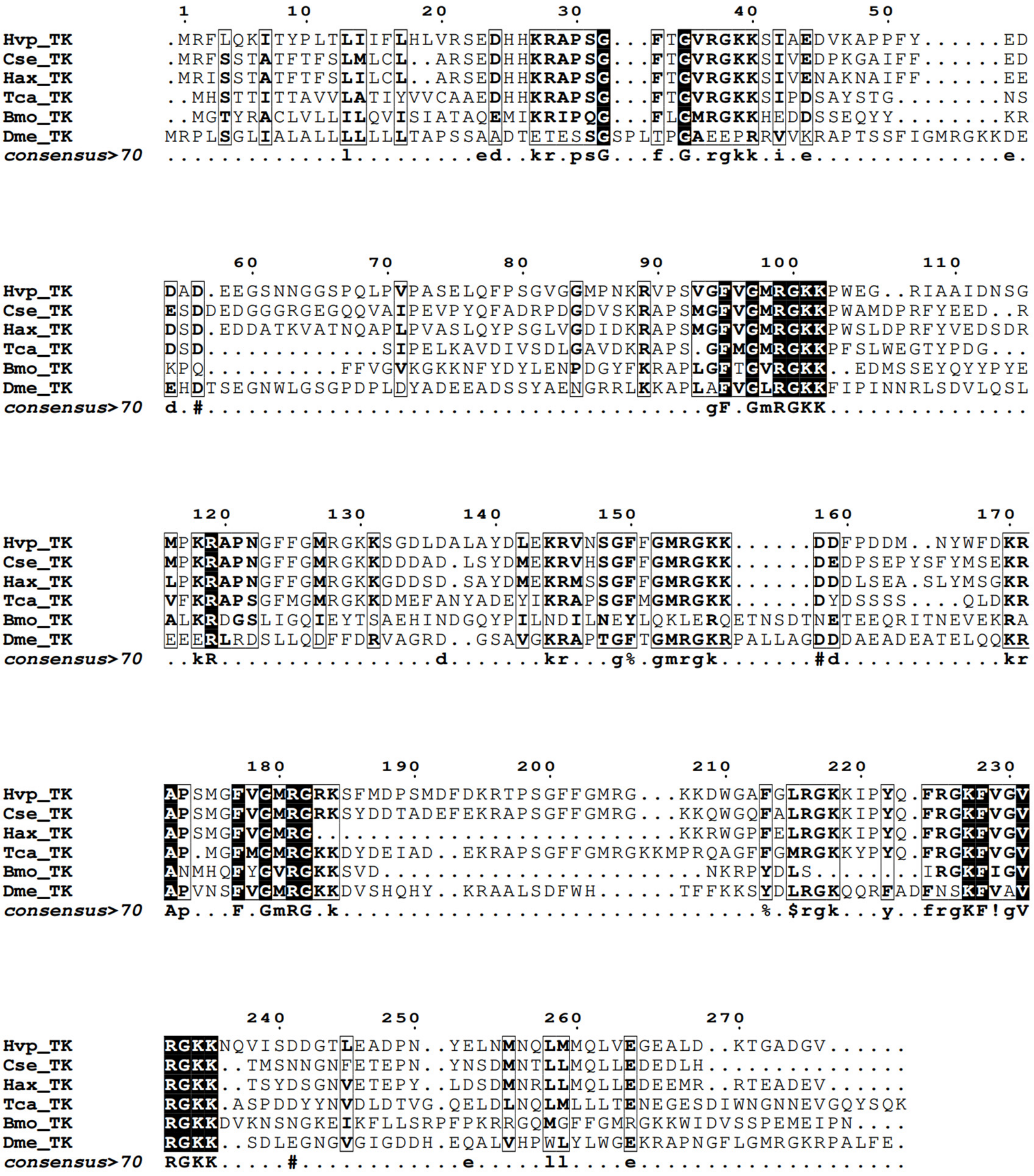

Figure S52. Sequence alignment of Tachykinin (TK) precursors.

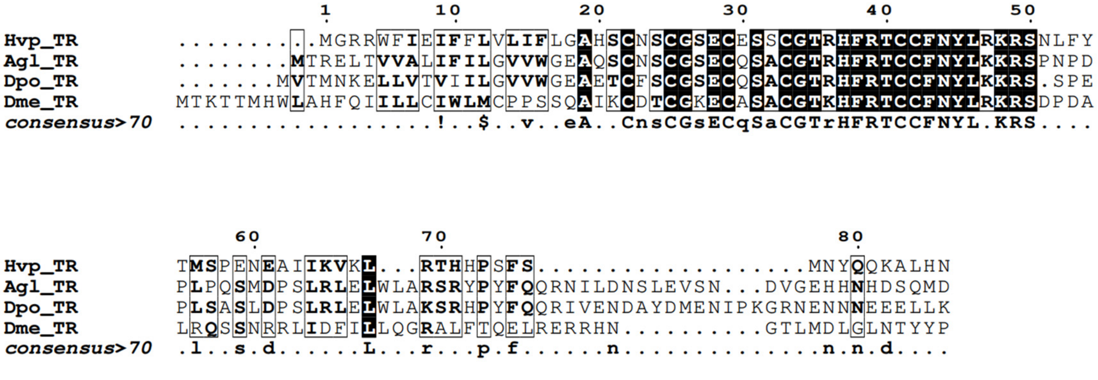

Figure S53. Sequence alignment of Trissin (TR) precursors.

References

Almagro Armenteros, J. J., Tsirigos, K. D., Sønderby, C. K., Petersen, T. N., Winther, O., Brunak, S., von Heijne, G., & Nielsen, H. (2019). SignalP 5.0 improves signal peptide predictions using deep neural networks. *Nat Biotechnol, 37*(4), 420-423. https://doi.org/10.1038/s41587-019-0036-z

Han, S., Chen, J., Liu, Z., Zhang, M., Guo, P., Liu, X., Wang, L., Shen, Z., & Zhang, L. (2024). Identification and expression profiling of neuropeptides and neuropeptide receptor genes in a natural enemy, Coccinella septempunctata. *Front Physiol, 15*, 1464989. https://doi.org/10.3389/fphys.2024.1464989

Hummon, A. B., Richmond, T. A., Verleyen, P., Baggerman, G., Huybrechts, J., Ewing, M. A., Vierstraete, E., Rodriguez-Zas, S. L., Schoofs, L., Robinson, G. E., & Sweedler, J. V. (2006). From the genome to the proteome: uncovering peptides in the Apis brain. *Science, 314*(5799), 647-649. https://doi.org/10.1126/science.1124128

Li, B., Predel, R., Neupert, S., Hauser, F., Tanaka, Y., Cazzamali, G., Williamson, M., Arakane, Y., Verleyen, P., Schoofs, L., Schachtner, J., Grimmelikhuijzen, C. J., & Park, Y. (2008). Genomics, transcriptomics, and peptidomics of neuropeptides and protein hormones in the red flour beetle Tribolium castaneum. *Genome Res, 18*(1), 113-122. https://doi.org/10.1101/gr.6714008

Marciniak, P., Pacholska-Bogalska, J., & Ragionieri, L. (2022). Neuropeptidomes of Tenebrio molitor L. and Zophobas atratus Fab. (Coleoptera, Polyphaga: Tenebrionidae). *J Proteome Res, 21*(10), 2247-2260. https://doi.org/10.1021/acs.jproteome.1c00694

Nässel, D. R., & Zandawala, M. (2019). Recent advances in neuropeptide signaling in Drosophila, from genes to physiology and behavior. *Prog Neurobiol, 179*, 101607. https://doi.org/10.1016/j.pneurobio.2019.02.003

Roller, L., Yamanaka, N., Watanabe, K., Daubnerová, I., Zitnan, D., Kataoka, H., & Tanaka, Y. (2008). The unique evolution of neuropeptide genes in the silkworm Bombyx mori. *Insect Biochem Mol Biol, 38*(12), 1147-1157. https://doi.org/10.1016/j.ibmb.2008.04.009

Tanaka, Y., Suetsugu, Y., Yamamoto, K., Noda, H., & Shinoda, T. (2014). Transcriptome analysis of neuropeptides and G-protein coupled receptors (GPCRs) for neuropeptides in the brown planthopper Nilaparvata lugens. *Peptides, 53*, 125-133. https://doi.org/10.1016/j.peptides.2013.07.027

Veenstra, J. A. (2019). Coleoptera genome and transcriptome sequences reveal numerous differences in neuropeptide signaling between species. *PeerJ, 7*, e7144. https://doi.org/10.7717/peerj.7144
